# Inferring RNA-binding protein target preferences using adversarial domain adaptation

**DOI:** 10.1371/journal.pcbi.1009863

**Published:** 2022-02-24

**Authors:** Ying Liu, Ruihui Li, Jiawei Luo, Zhaolei Zhang

**Affiliations:** 1 College of Computer Science and Electronic Engineering, Hunan University, Changsha, Hunan, China; 2 Donnelly Centre for Cellular and Biomolecular Research, University of Toronto, Toronto, Ontario, Canada; 3 Department of Computer Science and Engineering, The Chinese University of Hong Kong, Hong Kong, China; 4 Department of Computer Science, University of Toronto, Toronto, Ontario, Canada; 5 Department of Molecular Genetics, University of Toronto, Toronto, Ontario, Canada; Det Medisinske Fakultet, NTNU, NORWAY

## Abstract

Precise identification of target sites of RNA-binding proteins (RBP) is important to understand their biochemical and cellular functions. A large amount of experimental data is generated by in vivo and in vitro approaches. The binding preferences determined from these platforms share similar patterns but there are discernable differences between these datasets. Computational methods trained on one dataset do not always work well on another dataset. To address this problem which resembles the classic “domain shift” in deep learning, we adopted the adversarial domain adaptation (ADDA) technique and developed a framework (RBP-ADDA) that can extract RBP binding preferences from an integration of in vivo and vitro datasets. Compared with conventional methods, ADDA has the advantage of working with two input datasets, as it trains the initial neural network for each dataset individually, projects the two datasets onto a feature space, and uses an adversarial framework to derive an optimal network that achieves an optimal discriminative predictive power. In the first step, for each RBP, we include only the in vitro data to pre-train a source network and a task predictor. Next, for the same RBP, we initiate the target network by using the source network and use adversarial domain adaptation to update the target network using both in vitro and in vivo data. These two steps help leverage the in vitro data to improve the prediction on in vivo data, which is typically challenging with a lower signal-to-noise ratio. Finally, to further take the advantage of the fused source and target data, we fine-tune the task predictor using both data. We showed that RBP-ADDA achieved better performance in modeling in vivo RBP binding data than other existing methods as judged by Pearson correlations. It also improved predictive performance on in vitro datasets. We further applied augmentation operations on RBPs with less in vivo data to expand the input data and showed that it can improve prediction performances. Lastly, we explored the predictive interpretability of RBP-ADDA, where we quantified the contribution of the input features by Integrated Gradients and identified nucleotide positions that are important for RBP recognition.

## Author summary

RNA binding proteins (RBPs) regulate every aspect of RNA biology, including splicing, translation, transportation, and degradation. To fully understand the function of RBPs and the mechanism of RBP-RNA recognition, an accurate knowledge of the RBP-RNA binding preferences is essential. There are in vitro and in vivo experimental approaches such as RNAcompete or eCLIP that can determine RBP-RNA binding preferences in a high-through manner. However, because of the intrinsic differences between in vitro and in vivo experimental conditions, the binding preferences determined from in vitro and in vivo do not always agree with each other. To solve this problem and best utilize both types of data, we have adopted the adversarial domain adaptation (ADDA) technique into the analysis of RNA binding proteins and developed a framework (RBP-ADDA) that can extract RBP binding preferences from an integration of in vivo and vitro datasets. We showed that RBP-ADDA outperforms other contemporary methods in predicting RBA binding preferences on both in vivo and in vitro data. To the best of our knowledge, this is the first time that adversarial domain adaptation has been applied to the computational study of gene regulations.

## 1. Introduction

RNA-binding proteins (RBPs) have important roles in all aspects of post-transcriptional gene regulation including splicing, polyadenylation, transport, translation, and degradation of RNA transcripts [1]. Dysregulation of RBPs as well as mutations in their protein sequences or their RNA target sites can often result in diseases such as cancer [2,3]. Therefore, capturing the intrinsic binding preferences of RBPs and identifying their binding targets in a precise and high-throughput manner is essential to understand the regulatory roles of RBPs and reveal their connections to pathogenesis of human diseases.

Several experimental and computational platforms had been developed over the years to determine and model the binding preferences between RBPs and RNAs [4]. CLIP-seq and related techniques can identify in vivo binding events by immunoprecipitating RBPs and bound RNA molecules and identifying these bound RNAs through sequencing [5–9]. On the other hand, in vitro methods such as RNAcompete incubate protein with synthesized RNA fragments (typically 30–41 nucleotides long) and determine the identity of bound RNA sequence motifs by sequencing or microarray [10–12]. With the success of these experimental approaches, several computational methods had been developed with the goals of helping understand the binding preference from a structural and sequence perspective and building an accurate predictive model to infer binding affinities of other RBPs [13–19]. For example, MEMERIS uses an expectation maximization (EM) algorithm to look for sequence motifs in RNA regions that are more likely to be unpaired, and thus available for binding [13]. RNAcontext, an accompanying method with the RNAcompete technology, assigns secondary structures to RNA and learns a model simultaneously with sequence and structure features [14]. GraphProt encodes nucleotide sequence and RNA secondary structure by using graph encoding, which is then fed into support vector machines (SVMs) to classify bound sites from unbound sites [15]. Notably, the developers of GraphProt have constructed a representative dataset by extending 150 nucleotides in both directions on the binding sites determined in CLIP-seq; this positive dataset has been widely used to train deep learning (DL) based models such as iDeepE [20]. Ghanbari and Ohler recently proposed a multi-task and multimodal deep neural network to infer RBP binding sites by considering region types of the binding sites [19]. One of the earlier methods, DeepBind, learns a CNN model to predict protein-DNA and protein-RNA binding from several datasets, including RNAcompete and CLIP-seq[17]. Another deep learning-based method, DLPRB, performs joint analysis on both RNA sequence and structure by leveraging CNN and RNN [18].

There are intrinsic differences between in vivo and in vitro experimental approaches. Binding events determined by CLIP-seq and other in vivo methods tend to have lower signal-to-noise ratios and are influenced by cell-type specific effect, cooperation or competition between RBPs and other trans regulators [21]. RNA secondary structure and the choice of CLIP-seq peak callers are also known to introduce complexity and confounding effects [22,23]. Unlike in vivo methods, in vitro platforms measure protein-RNA binding affinities in a controlled setting thus the results typically have higher signal-to-noise ratio. However, it is often not clear whether the in vitro experimental conditions can mimic the complex conditions inside a cell and whether the in vitro determined binding affinities and sequence motifs can be readily extrapolated to in vivo situations [8,9,18]. It results in overall similarity but discernable differences between in vivo and in vitro data, thus existing methods designed on one dataset often do not perform well on other datasets. We note that this problem closely resembles the classic “domain shift” problem in deep learning, therefore we adapted the domain adaption principle onto the RBP recognition problem and describe our approach below.

In the realm of deep learning, domain adaptation methods attempt to mitigate the negative effect of domain shift when attempting to learn from multiple domains [24,25]. Domain adaptation methods learn deep neural transformations after mapping both domains onto a common feature space. This is generally achieved by optimizing representation of two domains in order to minimize a specific measure of domain shift such as maximum mean discrepancy [26,27] or correlation distances [28,29]. In addition to optimizing representation in these domains individually, an alternative approach is to reconstruct the target domain from the source representation [30], which can encode useful information from both domains and preserve discriminability. Commonly referred to as “adversarial adaptation methods” (ADDA), these methods seek to minimize an approximate domain discrepancy distance through an adversarial objective with respect to a domain discriminator [31]. These methods have been increasingly implemented in situations where information generated from two distinct domains share similarities, yet direct pooling of these data often introduce noises and contaminations. In the biological realm, adaptive approaches have been successfully implemented in biomedical image processing [32,33], gene expression analysis [34,35], and biological network reconstruction [36].

**Fig 1:**
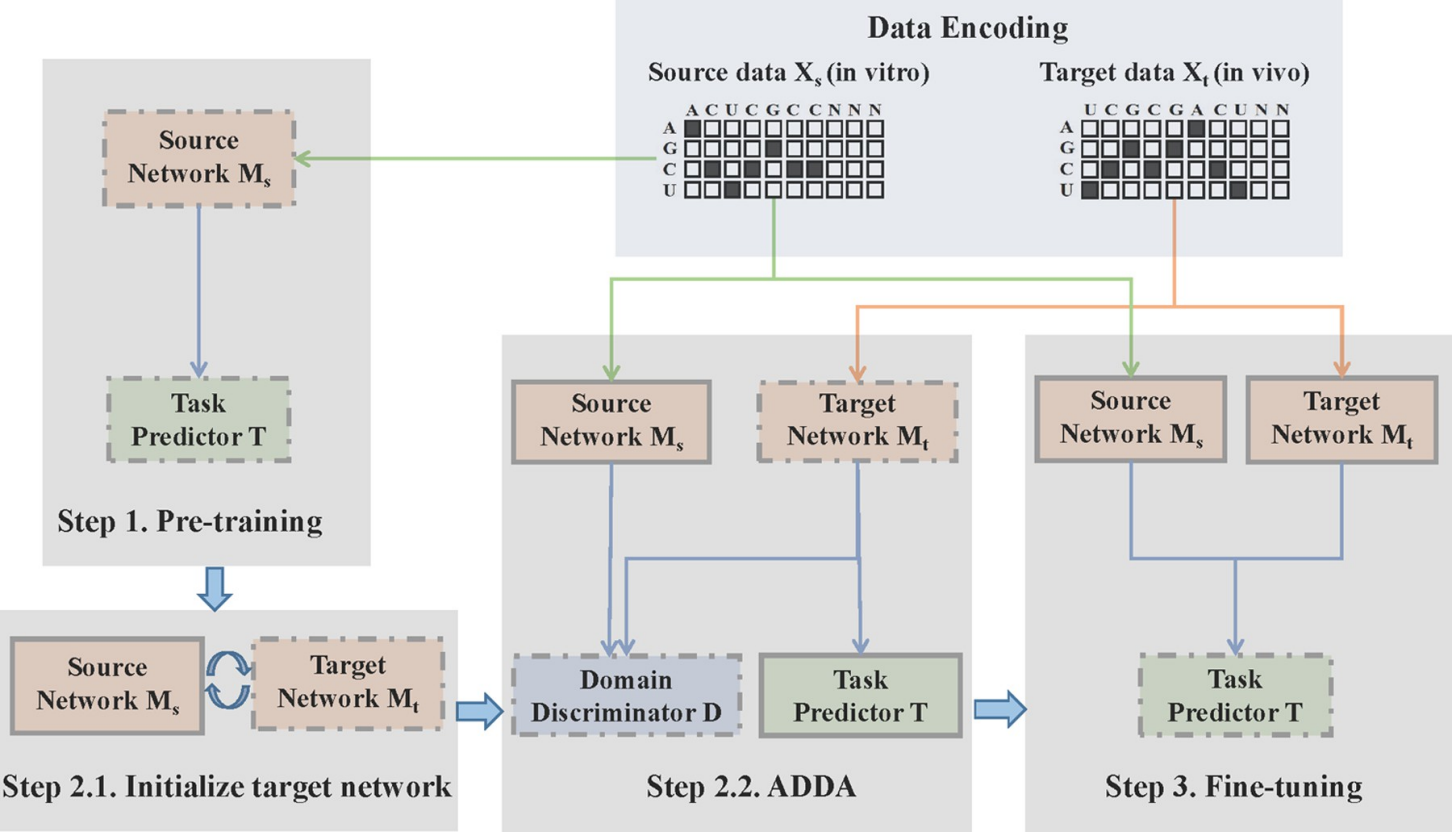
Flowchart of the RBP-ADDA method. During Data Encoding, each sequence in the sample (in vitro and in vivo) is represented as a concatenation of a one-hot encoding vector representing the nucleotides. **Step 1**. Pre-training. We use in vitro data to pre-train a source network and task predictor. **Step 2.1.** Initialize the target network. Target network is initialized by sharing the same parameters and architecture with source network. **Step 2.2**. ADDA. We apply adversarial learning to train the target network on in vivo data and train the domain discriminator. **Step 3**. Fine-tuning. We use both the source and target network to fine-tune the task predictor. Solid lines indicate steps in which the network parameters are fixed.

In this work, we describe RBP-ADDA, a deep neural network approach based on **A**dversarial **D**iscriminative **D**omain **A**daptation for learning RBP binding preferences. RBP-ADDA consists of three steps (see **Fig 1**). In Step 1, we use in vitro RBP binding data to pre-train a source network model and a task predictor model. In Step 2, we perform adversarial domain adaptation by learning a target network from in vivo data. In Step 3, we fine-tune the task predictor model based on both source data and target data. Since the in vitro RBP binding data has higher signal-to-noise ratio, a quality feature space can be learned from them. Projecting in vivo data into this space can help learn a better representation to improve the prediction performance of in vivo data. The fine-tuning step can make use of the complementarity between in vitro and in vivo data to improve their performance. Our experimental results demonstrated that the RBP-ADDA model not only improves the performance on modeling in vivo data but also improves the performance on in vitro data.

Furthermore, motivated by the success of data augmentation techniques in natural language processing (NLP), we applied three augmentation operators to improve the general performance of our model. Finally, to further explore the biological interpretability of our RBP-ADDA model, we used Integrated Gradients (IG) [37] to quantify the contribution of the input features to the predictive score of each RBP. We found that these critical features obtained from our model are consistent with the reported motifs and motifs derived from eCLIP and RNAcompete experiments. This illustrates the biological interpretability of our RBP-ADDA model.

To the best of our knowledge, we believe the RBP-ADDA model is the first method to apply adversarial domain adaptation to the analysis of interactions between RBPs and RNAs. This approach has the advantage of being able to learn from both in vitro and in vivo data which is otherwise not easy to achieve. We expect the adversarial domain adaptation approach can be extended to other relevant biological realms where both in vivo and in vitro datasets are available.

## 2. Materials and methods

### 2.1. Data collection and processing

We downloaded the vitro binding affinity data as determined by RNAcompete in July 2020 [12]. This dataset included 244 experiments, each experiment generating binding affinities of an RBP and more than 240,000 RNA fragments (30–41 nt in length). Among them, there were 102 experiments on 80 human RBPs. In RNAcompete experiments, the enrichment of each unique RNA fragment in the pulldown portion is calculated against the entire pool and the binding affinities are calculated as log ratios. We downloaded the in vivo eCLIP data generated by the ENCODE project [38], which had data from 150 RBPs (120 RBPs in K562 cells, 103 in HepG2 cells and 73 in both cell types). The signal value for each binding site was calculated as log2 (fold-enrichment).

In summary, there were 25 RBPs that had both in vivo and in vitro datasets. Among the RBPs that were studied by both RNAcompete and eCLIP via ENCODE, 19 RBPs had eCLIP data in K562 cell line and 19 RBPs had eCLIP data in HepG2 cell lines. The detail of each RBP dataset is shown in **S1 Table.** For each RBP, we randomly took an 80%/20% split as the training and testing sets without overlap. Further, to investigate whether there is significant sequence overlap between in vivo data and in vitro data, we conducted an all against all blastn search between in vivo data (10,921,666 sequences, 26~41nt in length) and in vitro data (241,357 sequences, 30~41nt in length). The detailed results are shown in **S3 Table**. Not surprisingly, there were noticeable overlap between the sequences from the in vivo and in vitro datasets since after all sequence motifs are the major determinant of the recognition process between RBP and RNAs. However, it is also clear that that the overlap is minimal since only a very small fraction of the sequences has any blastn matches (the last column in **S3 Table**).

To facilitate representation learning, we standardized the input data by scaling the labeled values to the range of [–1,1]. Since the in vivo CLIP-seq peaks are of various lengths, we processed them according to the following rules. If the peak fragments are shorter than 26 nt, we expand the fragment in both directions to the full-length of 26 nt; if the fragment is between 26 and 41 nt long, the original sequence is kept. If the fragment is longer than 41 nt, we evenly split this fragment into equal length with the overlap of 10 nt. The RNA fragments in the RNAcompete experiments have length ranging between 30 and 41, which was the reason we chose 41 nt as the cutoff.

### 2.2. Comparison with other methods

#### DeepBind

DeepBind combines multiple types of high-throughput data, including Protein Binding Array (PBM), RNAcompete, ChIP-Seq and HT-SELEX experiments, to derive binding affinities of RBPs and transcription factors [17]. It applies a convolutional neural network (CNN) to capture features from raw sequences and uses the trained CNN model to predict sequence binding preferences. We downloaded the trained model from http://tools.genes.toronto.edu/deepbind/ and applied it to both RNAcompete and eCLIP datasets using the default parameter to test the data and compare with our method.

#### DLPRB

DLPRB describes a novel deep neural network approach for learning intrinsic RBP binding preferences [18]. It integrates sequence and structural features of RBP binding sites to train a CNN model and applies the CNN model to predict sequence binding preferences. We downloaded the code from https://github.com/ilanbb/dlprb and trained the model using our own data. The RNA sequence structure information was measured by an adaptation of RNAplfold[39].

### 2.3. Sequence encoding

An RNA sequence is a string of nucleotides over the alphabet R (A; G; C; U). We encode each nucleotide as a one-hot vector of dimension 4. To standardize the input data, the length of each sequence is set as 41, which is also the maximum length of RNA fragments in the RNAcompete experiments. For those sequences with short length, we use ‘N’ to extend it to the same length, where ‘N’ is encoded by (0,0,0,0).

### 2.4. RBP-ADDA model

As shown in **Fig 1**, we take in vitro data (derived by RNAcompete) as source domain and in vivo data (from eCLIP) as target domain. Similar to DeepBind, DLPRB and other methods, we train a model individually for each RBP. Given the sequences *X*_*s*_ and labels *Y*_*s*_ drawn from a source domain distribution *p*_*s*_ *(x*,*y*), and sequences *X*_*t*_ and labels *Y*_*t*_ drawn from a target domain distribution *p*_*t*_*(x*,*y)*, our described model has the following two objectives. (i) Since the target in vivo data is usually more complex, we aim to employ the learned source representation to improve the performance on the target data learning via an adversarial domain adaptation process. (ii) Considering the complementarity between the data in the source and target domains, we aim to enhance the representation on both sides by further fine-tuning on these two data sets simultaneously.

**Fig 1** shows the overall architecture of our proposed RBP-ADDA algorithm, which consists of the following steps. In Step 1, we train the source network and the task predictor by using the labeled RNA sequences in the (in vitro) source domain. In Step 2, we perform adversarial adaptation and learn the target network and a discriminator. The discriminator is used to distinguish the representations derived from the source domain and from the target domain, respectively; the target network aims to fool the discriminator by producing target features that resembles the source representation. By doing so, we can take advantage of the well-learned source domain to improve the learning on the target domain. In Step 3, after mixing the source and target domains, we further fine-tune the task predictor on the source samples and target samples simultaneously to further extract complementary information from these two domains. The parameters of the source and target networks are fixed throughout this process. Such adversarial domain adaptation techniques have been shown to work well in problems such as biomedical image processing [32,33], gene expression analysis [34,35], and biological network reconstruction [36]. In the following, we describe in more details the individual steps in the algorithm.

#### 2.4.1 Source pre-training

As shown in **Fig 1**, after encoding the source (in vitro) data and the target (in vivo) data by using one-hot vectors to represent the nucleotides, we conduct pre-training on the sequences in the source domain. The pre-training involves a source network M_s_ and a task predictor T. The source network contains two convolution layers and two fully connected layers (S1A Fig). The convolution layers first apply a series of filters on the feature representation of the nucleotides to capture local patterns at the sequence level. A rectified linear unit (ReLU) is next applied to restrict to only positive matches [40]. A max pooling operation is next applied to reduce the dimensionality by selecting the maximum value over a window. A fully connected layer computes a weighted sum of the neurons from the previous layer. The task predictor contains two fully-connect layers and one output layer (S1B Fig). We used the following mean square error to regularize the whole network and update the parameters based on back propagation.


$$\underset{M_{s},T}{\min}L(X_{s},Y_{s})={\left(T(M_{s}(X_{s}))-Y_{s}\right)}^{2}$$ (1)

#### 2.4.2 Domain adaptation

Domain adaptation is an area in machine learning that deals with scenarios in which a model trained on a source distribution is applied in the context of a different but related target distribution [18,41]. The objective is to mitigate the harmful effect of domain shift. Adversarial adaptation method is a recent extension of the classic domain adaptation technique, which seeks to minimize an approximate domain discrepancy distance metric through a domain discriminator. These innovative methods have demonstrated to be very effective in biomedical image processing [32,33], gene expression analysis [34,35], and biological network reconstruction [36].

In our framework, we aim to employ the adversarial adaptation approach to minimize the domain distance between the source and target domains, i.e., the bound RNA sequences identified in the in vitro and in vivo experiments. Specifically, we train a target network M_t_ to share the same architecture as the source network so that it can produce the representation close to the source domain. We train the discriminator to maximize the domain difference between these two representations learned from source data and target data. Such an adversarial learning process can mix the target representation with the source representation. By doing so, we can use the well-trained source representation to improve the learning in the target domain. The detailed algorithm is described below in pseudocode.

**Algorithm 1** Training Strategy of RBP-ADDA model

**Input:** Source data samples and labels: X_s_, Y_s_

    Target data samples and labels: X_t_, Y_t_

Training iterations: n_1_,n_2_,n_3_; Batch size: m

**Output:** Source network: M_s_

    Target network: M_t_

    Discriminator: D

    Task predictor: T

        //Step 1: Source pre-training

1: **for** n_1_ training iterations **do**

2: Sample minibatch of m source samples from X_s_

3: Calculate the Mean Square Loss of source data using Eq (1)

4: Update the learnable parameters of Ms and T

5: **end for**

        // Step 2: Domain adaptation

6: Initial the parameters of M_t_ with M_s_

7: **for** n_2_ training iterations **do**

8: Sample minibatch of m source samples from X_s_

9: Sample minibatch of m target samples from X_t_

10: Calculate the discriminator loss using Eq (2) and update the parameters of D

11: Calculate the loss of target network using Eq (3) and update the parameters of M_t_

12: **end for**

        //Step 3: Model fine-tuning

13: **for** n_3_ training iterations **do**

14: Sample minibatch of m source samples from X_s_

15: Sample minibatch of m target samples from X_t_

16: Calculate the loss of task predictor using Eq (4) and update the parameters of T

17: **end for**

First, a domain discriminator D, which classifies whether a data point is originated from the source or the target domain, is optimized according to a loss function, L(X_s_,X_t_,M_s_,M_t_,D). The loss function is defined below:

$$\underset{D}{\min}L(X_{s},X_{t},M_{s},M_{t},D)=\frac{1}{2}({\left(D(M_{s}(X_{s}))-1\right)}^{2}+{\left(D(M_{t}(X_{t}))-0\right)}^{2})$$ (2)

Second, the source and target mappings are optimized according to a constrained adversarial objective. We select the optimization for the generator as follows, one part from the loss of discriminator and one part from the loss of target network:

$$\underset{M_{t}}{\min}L(X_{t},Y_{t},D)={\left(D(M_{t}(X_{t}))-1\right)}^{2}+{\left(T(M_{t}(X_{t}))-Y_{t}\right)}^{2}$$ (3)

#### 2.4.3 Model fine-tuning

Considering the complementary information contained in the source (in vitro) data and target (in vivo) data, we further fine-tuned the parameters of the task predictor on the source and target samples simultaneously by using the following loss function (Eq 4). We keep the parameters of source and target networks unchanged and alternate between the input source and target data. We used a smaller learning rate, lr = 0.00005, to fine-tune the parameters of the task predictor since we found the parameters trained on the source in vitro data were already performing well.


$$\underset{T}{\min}L(X_{s},X_{t},Y_{s},Y_{t})={\left(T(M_{s}(X_{s}))-Y_{s}\right)}^{2}+{\left(T(M_{t}(X_{t}))-Y_{t}\right)}^{2}$$ (4)

For testing, the RNA sequences from the target domain are fed into the target network, and subsequently mapped to the shared feature space together with the data from the source domain. The final prediction is made by the updated task predictor. Similarly, the source network takes the source sequences as input and the results are also predicted by the new updated predictor. We used Pearson correlation coefficients (PCC) between the predicted and actual probe intensities as metrics to evaluate the model performance. The Pearson correlations are also used to evaluate the prediction performance of RBPs in DeepBind and DLPRB.

### 2.5. Data augmentation

Data augmentation is a common strategy in machine learning in situations where the labeled training data is scarce, and the input data is artificially manipulated to enlarge the quantity and the diversity of the training samples [42]. Data augmentation has been effectively used in the areas of image recognition where Gaussian noises are added into the training images, and in natural language processing which used data noising as smoothing, and in predictive language models for synonym replacement. Data augmentation has also found success in biomedical research, for example, Chaudhari et al used generative adversarial networks to augment gene expression data for cancer classification [43]. Given the relative scarcity of the RBP binding data and the complexity of the experimental design, data augmentation could be a useful approach in generating additional data for model training.

In this work, we experimented with the following augmentation operations to enlarge the training dataset, i.e., the RNA fragments bound by RBPs. (i) Replacement: randomly choosing a nucleotide from the sequence and replacing it with its neighboring nucleotide. (ii) Swap: randomly choosing two nucleotides in the sequence and swap their positions. (iii) Gap: randomly choosing a nucleotide from the sequence and use the vector [0.3, 0.2, 0.2, 0.3] to replace one-hot encoder vector of this nucleotide. As a preliminary attempt, we only conducted data augmentation for RBP with a small amount of in vivo data, since it is hard to train a model with fewer samples. Also, the data augmentation was only conducted in the training samples and the modification on each sample was limited in a narrow range on a temporary basis, thus preventing permanent changes. We also experimented with two different hyper parameters: the number of nucleotides being replaced, swapped, or removed per sequence (0, 1, 2, 3, 4, or 5 nucleotides), and the percentage of RNA sequences in the training dataset that underwent augmentation (0%, 20%, 50%, 100%). The results of the fine-tuning showed that single nucleotide augmentation and 100% augmentation rate achieved the best performance.

### 2.6. Interpretation of RBP-ADDA predictions

Here we provide relevant biological intuition and interpretation of the RBP-ADDA model and its predictions. The overall objective of the model is to use adversarial domain adaptation to learn the RBP binding affinities from both in vitro and in vivo domains, which offers advantages over learning from only in vivo or in vitro. In addition, we also explored interpretation of the model, which can help us identify the structural or sequence features that contributed the most to the discriminative power of the model. Traditionally, in a deep neural network, the gradient (partial derivatives) of a neuron can be taken to approximate how much t the input features contribute to the output [44,45].

Given a set of RBP bound RNA sequences, we aim to ascertain which nucleotides of the input sequences are responsible for the positive prediction. Taking this concept, we employed an attribution-based method, i.e., integrated gradients (IG) [37]. As the input of the network traverses along a linear path from a baseline, IG computes the average gradients of the output to assign an attribution score to each input feature. The attribution score of each nucleotide indicates the importance of the nucleotide to the result we predict. Noted that the baseline is defined based on the application and we used a reference input that had the expected frequencies of A,C,G,U at each position (i.e., we set the ACGU channel axis to [0.3; 0.2; 0.2; 0.3]).

For a given RNA sequence, we calculated the attribution scores of every position and visualized the attribution scores as a sequence logo. The height of each position in the logo indicates the importance of each nucleotide position. For those locations with large positive attribution scores, the corresponding features can be interpreted as more informative for predicting RBP binding. There are some subtle differences between the attribution scores and the traditional positional specific weight matrices (PWM) which are often used to represent protein binding motifs. Attribution scores aim to identify the most discriminative position in the binding site while PWM indicate relative normalized frequency of each nucleotide at each position.

### 2.7. Implementation of RBP-ADDA

The RBP-ADDA is implemented in Python by using Tensorflow 1.15.0. We set the maximum number of epochs to 1000, and the batch size to 256. In the pre-training step, the learning rate was set at 0.001. In the domain adaptation step, the learning rates for target network and the discriminator were set at 0.001 and 0.00005, respectively. We set a small learning rate as 0.00005 for fine-tuning the task predictor. We evaluate all comparison models by using 5-fold cross-validation. The train and test time for different models are reported in **S3 Table**.

For the source and target networks (shown in **S1A Fig**), the number of filters for the two-convolution layers was set to 32. The filter sizes were set as 4x4 in the first convolution layer and 4x1 in the second convolution layer. The two fully connected layers have 128 hidden unis and 64 hidden units, respectively. The task predictor (shown in **S1A Fig**) consists of two fully connected layers of 64 and 32 hidden units, respectively, in addition to the prediction output. The discriminator consists of one fully connected layer of 64 hidden units and the adversarial discriminator output (**S1C Fig**). The hyper-parameters were optimized by a grid search process with the number of filters set at (16, 32, 64 and 128), the lengths of filter set at (4, 6, 8), the source and target network learning rate set at (0.01, 0.001 and 0.0001), and the discriminator learning rate (0.0001, 0.00005 and 0.00001).

## 3. Results

### 3.1. RBP-ADDA model achieves good performance on in vitro and in vivo data

**Fig 2:**
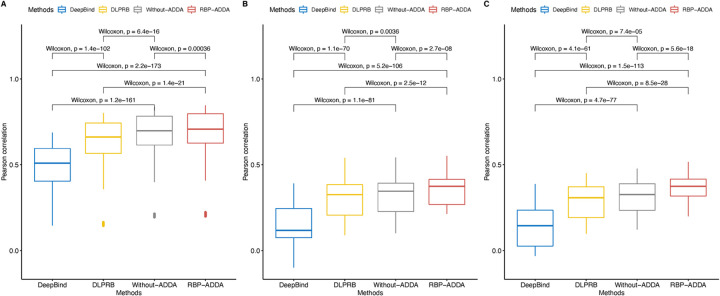
Comparison of performances between RBP-ADDA and other methods. **(A)** Comparison on 25 in vitro RNAcompete datasets; **(B)** Comparison on 19 eCLIP datasets from HepG2 cell line; **(C)** Comparison on 19 eCLIP datasets from K562 cell line. P-values are computed using unpaired Wilcoxon rank sum one-tailed test with p.adjust.

To evaluate the performance of our RBP-ADDA model, we compiled 25 in vitro datasets from RNAcompete, and 38 in vivo datasets generated by eCLIP, including 19 from HepG2 cell line and 19 from K562 cell line. Details about these datasets can be found in **Materials and Methods** (Section 2.1). In the following, we first discuss the prediction performance on the in vitro data. We conducted 5-fold cross-validation for each RBP dataset and quantified and compared the performance of the models via Pearson correlation of predicted and actual probe intensities. For each dataset of individual RBP, we withheld 20% of the bound RNA sequences and trained the RBP-ADDA and other models on the remaining 80% of the data. The models were then applied to the withheld data and Pearson correlation coefficients were calculated between the predicted and the observed probe intensities. We compared the performance of RBP-ADDA model with two other state-of-the-art approaches for predicting RBP binding sites, i.e. DeepBind [17] and DLPRB[16]. We note that it was not straightforward to compare with other prediction methods, either because the source code was not available, or it was difficult to train the models. As explained in **Materials and Methods**, we implemented DeepBind with a well-trained model provided by the author. For DLPRB, we downloaded the code and trained the model on our data. In addition, to evaluate the contribution of domain adaptation step, we also removed the domain adaptation step in RBP-ADDA and trained a spared-down version, shown as Without-ADDA in **Fig 2**.

As shown in **Fig 2,** our RBP-ADDA model significantly outperformed DeepBind and had marginally better performance than DLPRB and Without-ADDA. The detailed comparisons between RBP-ADDA and other methods on each RBP are shown in **S2 Table**. Across 25 in vitro experiments, our RBP-ADDA model achieved better performance than DeepBind, DLPRB and Without-ADDA, having Pearson correlation values ranging from 0.211 to 0.84 and a median value of 0.708 (**Fig 2A** and Table A in **S2 Table**). In contrast, DLPRB had Pearson correlation values between 0.155 and 0.796 with a median value of 0.658; DeepBind had Pearson correlation values between 0.154 and 0.684 with a median value of 0.508; Without-ADDA had Pearson correlation values between 0.205 and 0.828 with a median value of 0.698.

For in vivo data determined by eCLIP, as shown in Tables B and C in **S2 Table**, RBP-ADDA achieved the best Pearson correlation for 19 RBPs in HepG2 cell line and the best Pearson correlation of 19 RBPs in K562 cell line. The performance of DeepBind lags behind RBP-ADDA and DLPRB on in vivo data (**Fig 2B** and **2C**). We note that DeepBind was developed and trained primarily on in vitro RBP binding data, which explained why it did not perform well on the in vivo eCLIP data. **Fig 2** and **S2 Table** show that DLPRB achieved comparable performance as our RBP-ADDA model. Note that DLPRB method integrates both RNA sequence and structural information into the prediction framework, while our RBP-ADDA model requires RNA sequence information only.

As shown in **Fig 2B** and **2C**, the RBP-ADDA model significantly outperformed the Without-ADDA model on in vivo data, validating the effectiveness of the domain adaptation approach. On the other hand, on in vitro data (**Fig 2A**), Without-ADDA also significantly outperformed other methods, while the performance improvement on in vivo data was marginal. This further confirmed our rationale to leverage domain adaption technique, where we use in vitro data as the source data to achieve a better prediction on in vivo data.

As explained in Introduction, in vitro RBP-RNA binding datasets intrinsically have higher signal-to-noise ratio than in vivo datasets, thus it is understandable that the prediction performance on in vitro data was generally better than that on in vivo data. Therefore, when conducting domain adaptation, it is preferable to take in vitro data as source data, providing a better latent representation. To further explore this idea, we trained a “reversed” model using in vivo data as source and in vitro data as target. Indeed, the comparison results (shown in **S5 Table**) confirmed that the RBP-ADDA model with in vitro data as source data generally performed better the “reversed” models on both in vitro and in vivo.

### 3.2. The role of individual steps in RBP-ADDA

**Fig 3:**
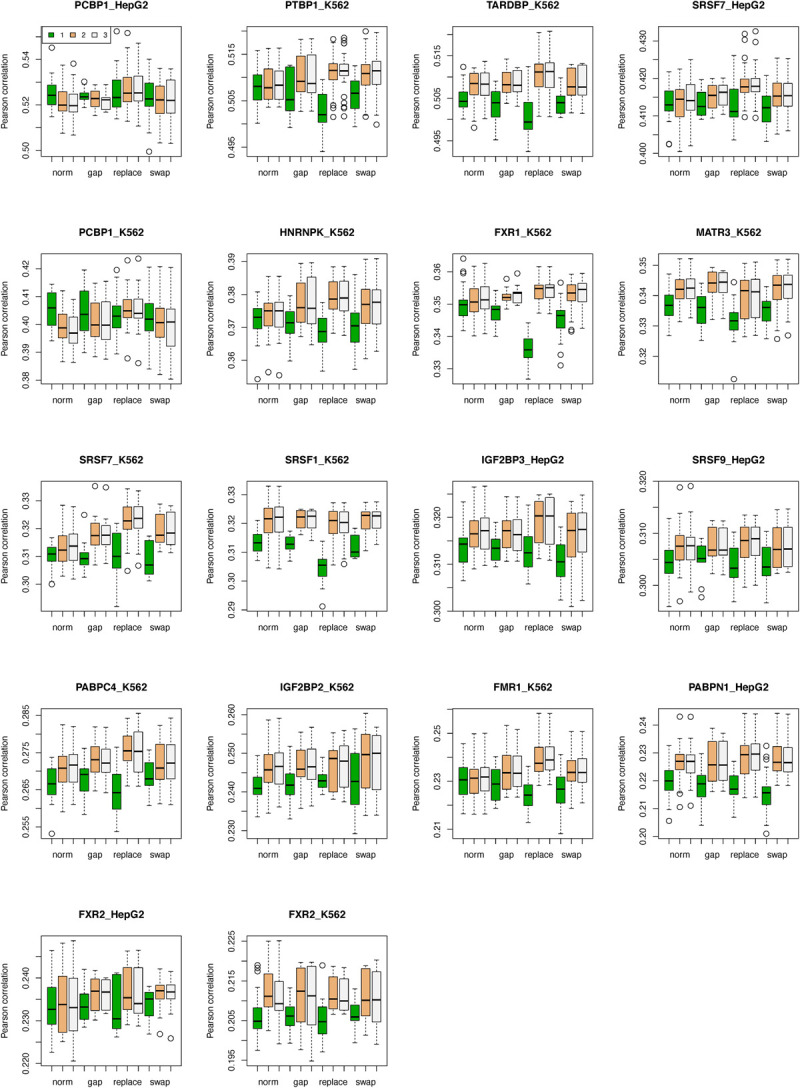
Performance of RBP-ADDA model after data augmentation operations. In each panel, the predictive performances on an RBP are grouped and shown as norm (non-augment), gap, replacement, and swap. Within each group, the performances after pre-training step, domain adaptation step and fine-tuning step are indicated as “1”, “2” and “3”.

The RBP-ADDA model consists of three steps: pre-training, domain adaptation and fine-tuning. As shown in **S2 Fig**, the prediction performance of most RBPs has improved after domain adaptation was applied. For example, the Pearson correlation of PABPN1 in HepG2 cell line increased from 0.218 to 0.227 (**S2A Fig**), and the Pearson correlation of SRSF1 in K562 cell line increased from 0.315 to 0.322 (**S2B Fig**). It is likely that the primary reason for such improved performance on in vivo data was the relatively good prediction results on the source (in vitro) data, with Pearson correlation at 0.613 and 0.723 respectively, which provided better feature representation for these two RBPs. Hence, when conducting domain adaptation, the in vitro source data can provide a well-initialized learning space for the corresponding RBPs on in vivo data, improving the predictive performance. This also validated the effectiveness of our domain adaptation approach, as it reduces the domain shift between source data and target data and improves the prediction performance on target data. We notice that domain adaptation has slightly negative effect on a few RBPs, where there is a large difference on the size of training samples between source data and target data, such as PCBP1(**Fig 3** and **S1 Table**). Such an imbalanced distribution may be a barrier to integrate these two kinds of data. Lastly, we validated the effect of the fine-tuning step, which can further take the complementary advantage from pooled features in two domains. These results showed that there was moderate increase for almost all RBPs on in vitro data (**S2C Fig**), while most RBPs also have slight improvements on in vivo data, particularly for those RBPs with more in vivo samples (**S2A** and **S2B Fig**).

### 3.3. Effectiveness of the augmentation operations

As described in Section 2.5, we applied three augmentation operators, including Gap, Replacement and Swap, to improve the generalization of our RBP-ADDA model. We experimented with two hyper parameters in the augmentation step and evaluated how the predictive performance has improved. **S3 Fig** shows the results after we replaced, swapped, or removed 1, 2, 3, 4, or 5 nucleotides in each RNA sequence. Since RNA secondary structures are sensitive to nucleotide mutations, it is likely that mutations or replacement of two or more nucleotides would disrupt RNA secondary structure and introduce noises to the training data. In this case, single nucleotide operations generally were preferred.

**S4 Fig** shows the results after we augmented 0%, 20%, 50%, or 100% of the sequences in the training dataset; in this case only the single nucleotide augmentation per sequence is shown. The predictive performances fluctuated with increasing percentage of augmented sequences; for the majority of the RBPs, the 100% augmentation rate gave rise to the highest predictive performance, which was the parameter we chose to use in this study.

As shown in **Fig 3**, in most cases, the RBP-ADDA model trained with these techniques performed better than the original model. Among these three techniques, the Replacement operator, which randomly chose a nucleotide and replaced it with its neighboring nucleotide, significantly improved the prediction performance, especially in FRM1_K562, SRSF7_K562, PABPC4_K562, PABPN1_HepG2 with a 4.1%, 4.2%, 3.1%, 4.1% increase in the Pearson correlation, respectively. We noticed that the augmentation techniques may have a slightly negative effect on the performance of pre-training network, since the augmentation process may introduce some noisy samples. Nevertheless, the augmented samples introduce more internal variations, thus contributing to a better generalization capability and preventing overfitting.

### 3.4 Interpretation of RBP-ADDA model

**Fig 4:**
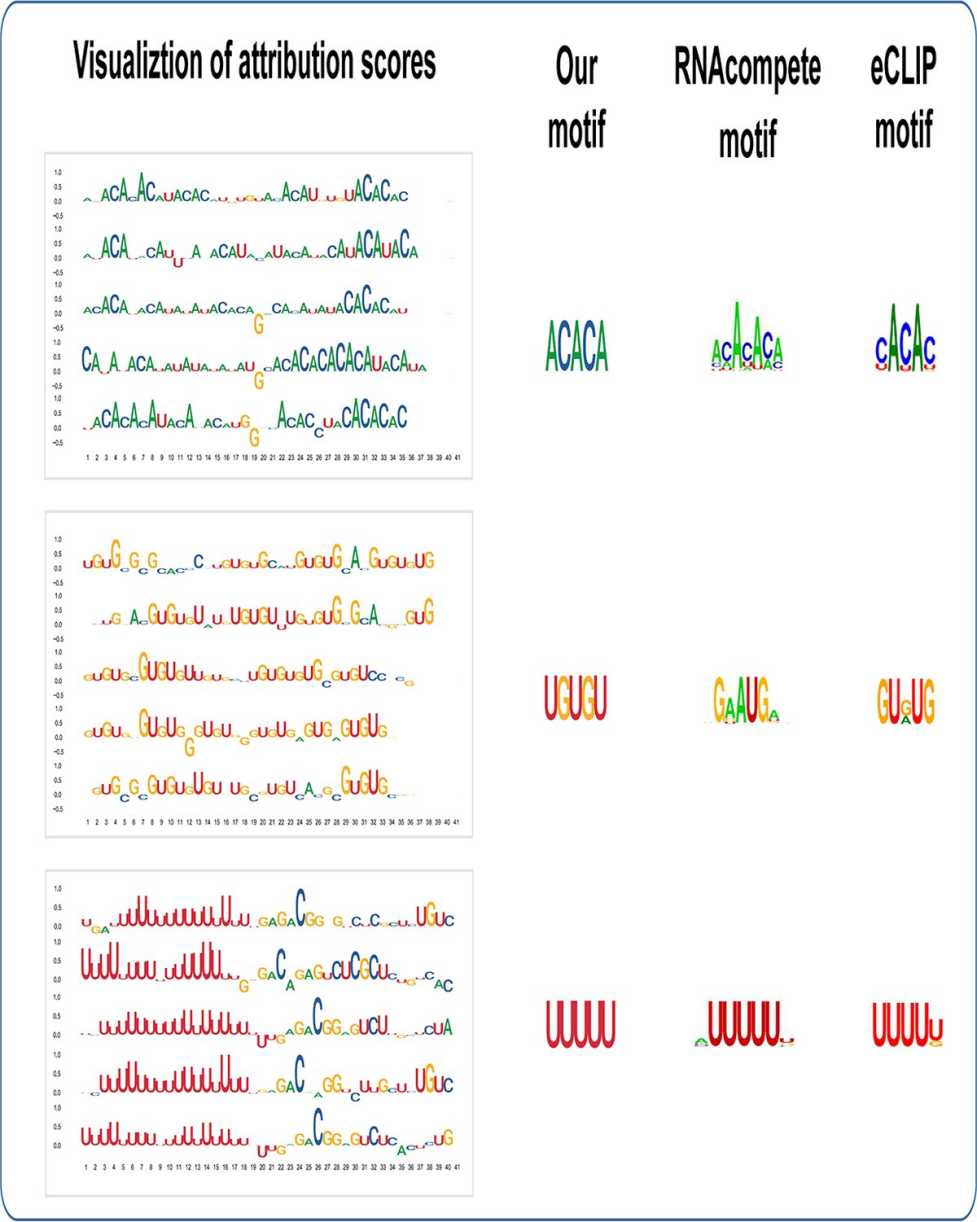
Visualization of attribution scores, consensus motif, motifs obtained from in vitro (RNAcompete) and in vivo (eCLIP) experiments.

Here, we explore the interpretation of the RBP-ADDA model to better understand which input features contribute the most to the improvement in predictive performance. To do so, as described in Section 2.6, we employed an attribution score based method, integrated gradients (IG) [37]. Specifically, for a given RBP, we calculated an attribution score for each nucleotide in the input sequence. The attribution scores quantified the contribution of each nucleotide to the discriminative performance in separating the positive and negative binding sites. For each RBP, we selected sequences with the top 5 highest prediction scores (when higher than 0.5) and visualized those attribution scores corresponding to the sequence, as shown on the left side in **Figs 4** and **S5**. We plotted **Fig 4** with R package “ggseqlogo” [46]. The heights of nucleotides represent the magnitude of attribution scores; the positive or negative scores are plotted above or below the horizontal axis. Positive attribution scores contribute to be a binding site and negative attribution scores have a negative influenced to be a binding site. The nucleotides with small attribution scores have neutral contributions.

We further explored the consensus representations of the binding sites of each RBP. We retained those positive RNA fragments with prediction scores higher than 0.5 and derived 5-mers with the highest attribution scores from these fragments and derived consensus motifs (**Fig 4** middle panel, and **S6 Fig).** We note that these consensus 5-mers largely agree with the known motifs derived from RNAcompete and eCLIP. For example, HNRNPL exhibits a binding preference for CA-rich elements [47]; TARDBP preferentially binds to GU-repeats [48] and HNRNPC is known to bind poly-U tracts [49].

In summary, **Figs 4** and **S1–S6** showed that the ADDA approach can successfully capture the binding preference of each RBP and identify the nucleotide positions on the RNA that are important in the recognition process.

## 4. Discussion

In this paper, we present RBP-ADDA, a deep neural network approach based on **A**dversarial **D**iscriminative **D**omain **A**daptation to learn RBP binding preferences by integrating in vivo and vitro datasets. Motivated by the observation that in vitro and in vivo RBP binding data share similar patterns, we employed adversarial discriminative domain adaptation to mitigate the difference between in vitro and in vivo domains. Our model projects the two datasets onto a shared feature space and uses an adversarial framework to derive an optimal network that achieves optimal discriminative predictive power. Compared to other recently published methods such as DeepBind and DLPRB, our RBP-ADDA can achieve better prediction performances on 38 eCLIP datasets and 25 RNAcompete experiments.

To the best of our knowledge, this is the first reported application of adversarial domain adaptation approach in the realm of DNA or RNA sequence motifs. We demonstrated the effectiveness of this approach in integrating multiple datasets and maximizing the value of heterogenous datasets. It is worth mentioning that Cohn and colleagues also applied adversarial learning to generate negative samples for transcriptional enhancer motifs identifying [50].

To further improve the generalization of our RBP-ADDA model, we introduced three augmentation operators, including Gap, Replacement, and Swap. These operators can enrich the quantity and the diversity of the training samples. As shown in **Fig 3**, the RBP-ADDA model with these operators can achieve better prediction performance on RBPs that have small amount of training samples, especially by using Replacement operator. As in other data augmentation approaches, it is important to fine-tune the hyper parameters to achieve the best performance. In the case of RBP-RNA recognition, we explored the optimal number of replaced nucleotides per sequence and the fraction of sequences augmented in the input training data set (**S3** and **S4 Figs**). We recommend researchers always evaluate these parameters when applying data augmentation in the biological domain and always be mindful that the augmentation operations are biologically meaningful. Since single nucleotide mutations and natural variations are often tolerated in RBP binding sites [38,51], we are confident that the single nucleotide replacement operations did not drastically disrupt RNA structure elements and introduce unnecessary noises to the model.

Finally, we explored the interpretability of our RBP-ADDA model by ascertaining the influence of each nucleotide in the input sequence on their contribution to the discriminative power of the model. We showed that the attribution scores calculated for each nucleotide position are consistent with previously reported motifs as determined by in vivo or in vitro approaches. We like to note that in the context of this work, the term “motif” strictly refers to short, contiguous, linear RNA sequences. A majority of the RBPs that have been experimentally studied are thought to recognize these linear and single stranded RNA motifs. In fact, the local accessibility of RNA motifs has been widely adapted in previously published software tools [14,20,23,52]. Despite such an attractive framework, recent advances in RBP studies showed that certain RBPs break such simple rules and can recognize other RNA secondary structure elements such as folded hairpins [53]. New approaches such as icSHAPE, which can measure in vivo RNA accessibility [54], and more advanced computational tools that can extract enriched RNA secondary structure motifs [55,56].

By definition, the RBP-ADDA model is designed only for RBPs that have both in vitro and in vivo data, which is a pre-requisite for the concept of domain adaptation. There are many RBPs that only have experimentally determined in vitro or in vivo binding data but not both. It is interesting to explore whether it is feasible or effective to first infer the binding data for the missing domain, and then apply the domain adaptation approach. It is relatively feasible to infer the in vitro binding affinities for a new RBP if binding affinities are known for a large number of evolutionarily related homologous RBPs [57]. Additional protein structure information on these RBPs would also be useful to improve the prediction accuracy, i.e., the prior knowledge on which amino acid residues are involved in the RBP-RNA binding process. Mutation data on the RBP sequence, either derived from population cohorts, or from high-throughput cell based functional assays, are also helpful in finding important RBPs or important amino acid residues on these RBPs in a disease context [58,59].

## Supporting information

S1 Table Detailed statistics of the pre-processed datasets. (DOCX)

S2 Table Detailed comparisons between RBP-ADDA and other methods. (DOCX)

S3 Table The overlap of in vivo data and in vitro data. (DOCX)

S4 Table Runtime of different methods. (DOCX)

S5 Table Detailed comparisons between RBP-ADDA and the “reversed” method. (DOCX)

S1 Fig Detailed structure of RBP-ADDA model. (A). The architecture of Source and Target Network. (B). The architecture of the Task Predictor. (C). The architecture of the Discriminator.
(PDF)

S2 Fig Prediction performance of RBP-ADDA model at different steps of the pipeline. “1”, “2”, and “3” represent the pre-training, domain adaptation, and model fine-tuning steps respectively. For source data, we update the source model only in pre-training and fine-tuning. (A) Performance at different steps on target in vivo data tested in HepG2 cell lines. (B) Performance on target in vivo data tested in K562 cell line. (C) Performance of tested on source in vitro data.
(PDF)

S3 Fig Impact of data augmentation on predictive performance when different number of nucleotides are swapped, replaced, or deleted. (PDF)

S4 Fig Impact of data augmentation on predictive performance when different fraction of the training data is altered. Only single nucleotide augmentation is applied and shown here.
(PDF)

S5 Fig Attribution scores of top-5 RNA fragment that have the highest prediction scores. For each RBP, the top 5 RNA fragments are visualized with attribution scores calculated following integrated gradients (IG) method.
(PDF)

S6 Fig Consensus motifs of each RBP. For each RBP, we extracted 5-mers with the highest average attribution scores and derived two consensus motifs.
(PDF)
