# Supplementary material for: Inferring RNA-binding protein target preferences using adversarial domain adaptation
Source: PLoS Comput Biol. 2022 Feb 24;18(2):e1009863. doi: 10.1371/journal.pcbi.1009863 (PMC8870515; doi:10.1371/journal.pcbi.1009863)
Supplement: S1 Table — (DOCX) [file pcbi.1009863.s001.docx]

**Table S1**. Detailed statistics of pre-processed datasets**.**

A. Data sets from K562 cell line (sorted by size of in vivo data)

| **RBP** | **In vitro** | **In vivo** | **RNAcompete_ID** | **ENCODE_ID** |
| --- | --- | --- | --- | --- |
| PCBP1_K562 | 241357 | 42888 | RNCMPT00186 | ENCSR922WJV |
| FMR1_K562 | 241357 | 105981 | RNCMPT00016 | ENCSR331VNX |
| HNRNPK_K562 | 241357 | 109986 | RNCMPT00026 | ENCSR268ETU |
| PTBP1_K562 | 241357 | 114442 | RNCMPT00268 | ENCSR981WKN |
| SRSF7_K562 | 241357 | 117511 | RNCMPT00073 | ENCSR468FSW |
| PABPC4_K562 | 241357 | 117724 | RNCMPT00043 | ENCSR958FKZ |
| FXR2_K562 | 241357 | 120852 | RNCMPT00020 | ENCSR224QWC |
| TARDBP_K562 | 241357 | 131282 | RNCMPT00076 | ENCSR584TCR |
| MATR3_K562 | 241357 | 135402 | RNCMPT00037 | ENCSR440SUX |
| IGF2BP2_K562 | 241357 | 148409 | RNCMPT00033 | ENCSR062NNB |
| SRSF1_K562 | 241357 | 187378 | RNCMPT00106 | ENCSR432XUP |
| FXR1_K562 | 241357 | 203795 | RNCMPT00161 | ENCSR774RFN |
| U2AF2_K562 | 241357 | 248496 | RNCMPT00079 | ENCSR893RAV |
| HNRNPC_K562 | 241357 | 268695 | RNCMPT00025 | ENCSR249ROI |
| FUS_K562 | 241357 | 344795 | RNCMPT00018 | ENCSR069EVH |
| TIA1_K562 | 241357 | 412568 | RNCMPT00077 | ENCSR057DWB |
| HNRNPL_K562 | 241357 | 468389 | RNCMPT00027 | ENCSR795CAI |
| HNRNPA1_K562 | 241357 | 495784 | RNCMPT00022 | ENCSR154HRN |
| KHDRBS1_K562 | 241357 | 496712 | RNCMPT00169 | ENCSR628IDK |

B. Data sets from HepG2 cell line (sorted by size of in vivo data)

| **RBP** | **In vitro** | **In vivo** | **RNAcompete_ID** | **ENCODE_ID** |
| --- | --- | --- | --- | --- |
| PCBP1_HepG2 | 241357 | 46234 | RNCMPT00186 | ENCSR256CHX |
| FXR2_HepG2 | 241357 | 115749 | RNCMPT00020 | ENCSR973HOJ |
| SRSF7_HepG2 | 241357 | 133186 | RNCMPT00073 | ENCSR513NDD |
| PABPN1_HepG2 | 241357 | 146089 | RNCMPT00157 | ENCSR820UYE |
| SRSF9_HepG2 | 241357 | 207812 | RNCMPT00067 | ENCSR773KRC |
| IGF2BP3_HepG2 | 241357 | 237152 | RNCMPT00172 | ENCSR993OLA |
| PCBP2_HepG2 | 241357 | 241448 | RNCMPT00044 | ENCSR339FUY |
| HNRNPK_HepG2 | 241357 | 248288 | RNCMPT00026 | ENCSR828ZID |
| TIA1_HepG2 | 241357 | 252147 | RNCMPT00077 | ENCSR623VEQ |
| RBM5_HepG2 | 241357 | 285175 | RNCMPT00055 | ENCSR489ABS |
| SRSF1_HepG2 | 241357 | 321790 | RNCMPT00106 | ENCSR989VIY |
| FUS_HepG2 | 241357 | 352546 | RNCMPT00018 | ENCSR464OSH |
| MATR3_HepG2 | 241357 | 353017 | RNCMPT00037 | ENCSR290VLT |
| PTBP1_HepG2 | 241357 | 357421 | RNCMPT00268 | ENCSR384KAN |
| SFPQ_HepG2 | 241357 | 388397 | RNCMPT00177 | ENCSR965DLL |
| U2AF2_HepG2 | 241357 | 410461 | RNCMPT00079 | ENCSR202BFN |
| HNRNPL_HepG2 | 241357 | 635269 | RNCMPT00027 | ENCSR724RDN |
| HNRNPA1_HepG2 | 241357 | 897409 | RNCMPT00022 | ENCSR769UEW |
| HNRNPC_HepG2 | 241357 | 1020987 | RNCMPT00025 | ENCSR550DVK |
