# Supplementary material for: Inferring RNA-binding protein target preferences using adversarial domain adaptation
Source: PLoS Comput Biol. 2022 Feb 24;18(2):e1009863. doi: 10.1371/journal.pcbi.1009863 (PMC8870515; doi:10.1371/journal.pcbi.1009863)
Supplement: S2 Table — (DOCX) [file pcbi.1009863.s002.docx]

**Supplemental Table S2.**

Performance comparisons between RBP-ADDA and other methods. We applied random 5-fold cross-validation to evaluate each method. The performances were evaluated by Pearson Correlation; the averaged PCC (mean) and standard deviations are shown for each method. Table S2A shows the comparison for in vitro RNAcompete data while Table S2B and Table S2C show the comparison for in vivo data derived from different cell lines.

1. **25 RBPs from RNAcompete**

| **RBP** | **Deepbind** | **DLPRB** | **Without_ADDA** | **RBP-ADDA** |
| --- | --- | --- | --- | --- |
| **PCBP1** | 0.311 ± 0.0046 | 0.366 ± 0.0071 | 0.411 ± 0.0044 | **0.418 ± 0.0038** |
| **FMR1** | 0.62 ± 0.0034 | 0.676 ± 0.0046 | 0.74 ± 0.0026 | **0.752 ± 0.002** |
| **HNRNPK** | 0.514 ± 0.0039 | 0.61 ± 0.0028 | 0.645 ± 0.0036 | **0.652 ± 0.0042** |
| **PTBP1** | 0.637 ± 0.0025 | 0.796 ± 0.0024 | 0.828 ± 0.0022 | **0.837 ± 0.002** |
| **SRSF7** | 0.574 ± 0.0023 | 0.658 ± 0.0053 | 0.698 ± 0.0041 | **0.708 ± 0.0037** |
| **PABPC4** | 0.609 ± 0.0033 | 0.777 ± 0.0045 | 0.821 ± 0.0028 | **0.832 ± 0.0021** |
| **FXR2** | 0.406 ± 0.0033 | 0.4 ± 0.004 | 0.458 ± 0.0042 | **0.469 ± 0.0033** |
| **TARDBP** | 0.576 ± 0.0037 | 0.715 ± 0.0047 | 0.752 ± 0.0023 | **0.763 ± 0.0028** |
| **MATR3** | 0.513 ± 0.0027 | 0.707 ± 0.0049 | 0.752 ± 0.0031 | **0.764 ± 0.0025** |
| **IGF2BP2** | 0.52 ± 0.0026 | 0.532 ± 0.0048 | 0.601 ± 0.0041 | **0.613 ± 0.0037** |
| **SRSF1** | 0.446 ± 0.0045 | 0.686 ± 0.0027 | 0.716 ± 0.003 | **0.723 ± 0.0034** |
| **FXR1** | 0.463 ± 0.0027 | 0.575 ± 0.004 | 0.635 ± 0.0028 | **0.649 ± 0.0048** |
| **U2AF2** | 0.345 ± 0.0051 | 0.567 ± 0.0038 | 0.613 ± 0.0042 | **0.625 ± 0.0036** |
| **HNRNPC** | 0.425 ± 0.0041 | 0.645 ± 0.0031 | 0.67 ± 0.0043 | **0.679 ± 0.0037** |
| **FUS** | 0.364 ± 0.0039 | 0.567 ± 0.008 | 0.623 ± 0.0035 | **0.639 ± 0.0023** |
| **TIA1** | 0.611 ± 0.0021 | 0.756 ± 0.0051 | 0.815 ± 0.0028 | **0.828 ± 0.0024** |
| **HNRNPL** | 0.592 ± 0.0028 | 0.747 ± 0.0031 | 0.785 ± 0.0032 | **0.798 ± 0.0026** |
| **HNRNPA1** | 0.684 ± 0.0023 | 0.791 ± 0.0032 | 0.828 ± 0.0038 | **0.84 ± 0.0024** |
| **KHDRBS1** | 0.508 ± 0.0033 | 0.731 ± 0.003 | 0.766 ± 0.0033 | **0.775 ± 0.0021** |
| **PABPN1** | 0.443 ± 0.0056 | 0.572 ± 0.0046 | 0.604 ± 0.0056 | **0.613 ± 0.0031** |
| **SRSF9** | 0.434 ± 0.0045 | 0.482 ± 0.0033 | 0.518 ± 0.0043 | **0.527 ± 0.0037** |
| **IGF2BP3** | 0.387 ± 0.0028 | 0.58 ± 0.0057 | 0.616 ± 0.005 | **0.626 ± 0.0049** |
| **PCBP2** | 0.154 ± 0.0037 | 0.155 ± 0.0054 | 0.205 ± 0.0047 | **0.211 ± 0.0059** |
| **RBM5** | 0.605 ± 0.0031 | 0.675 ± 0.0028 | 0.702 ± 0.0027 | **0.71 ± 0.0027** |
| **SFPQ** | 0.335 ± 0.0035 | 0.754 ± 0.0044 | 0.813 ± 0.0025 | **0.828 ± 0.0022** |

**B. 19 RBPs on eCLIP data in HepG2 cell line**

| **RBP** | **Deepbind** | **DLPRB** | **Without_ADDA** | **RBP-ADDA** |
| --- | --- | --- | --- | --- |
| **PCBP1_HepG2** | 0.249 ± 0.0075 | 0.436 ± 0.0043 | 0.452 ± 0.015 | **0.521** ± 0.0075 |
| **FXR2_HepG2** | 0.109 ± 0.0048 | 0.1 ± 0.0066 | 0.136 ± 0.0173 | **0.234** ± 0.0074 |
| **SRSF7_HepG2** | -0.085 ± 0.0053 | 0.328 ± 0.0064 | 0.365 ± 0.0108 | **0.414** ± 0.0055 |
| **PABPN1_HepG2** | 0.046 ± 0.0055 | 0.135 ± 0.0063 | 0.148 ± 0.0101 | **0.226** ± 0.0079 |
| **SRSF9_HepG2** | 0.1 ± 0.0063 | 0.252 ± 0.0043 | 0.266 ± 0.0091 | **0.307** ± 0.0041 |
| **IGF2BP3_HepG2** | 0.056 ± 0.0048 | 0.272 ± 0.0044 | 0.278 ± 0.0062 | **0.317** ± 0.0046 |
| **PCBP2_HepG2** | 0.166 ± 0.0043 | 0.362 ± 0.0037 | 0.387 ± 0.0075 | **0.412** ± 0.0028 |
| **HNRNPK_HepG2** | 0.315 ± 0.0036 | 0.457 ± 0.0042 | 0.477 ± 0.0044 | **0.495** ± 0.0036 |
| **TIA1_HepG2** | 0.257 ± 0.0037 | 0.315 ± 0.0039 | 0.346 ± 0.0067 | **0.383** ± 0.0045 |
| **RBM5_HepG2** | 0.093 ± 0.0041 | 0.203 ± 0.005 | 0.215 ± 0.009 | **0.259** ± 0.0061 |
| **SRSF1_HepG2** | 0.119 ± 0.003 | 0.334 ± 0.0046 | 0.335 ± 0.0082 | **0.373** ± 0.0026 |
| **FUS_HepG2** | 0.008 ± 0.0034 | 0.148 ± 0.0097 | 0.172 ± 0.011 | **0.25** ± 0.0051 |
| **MATR3_HepG2** | 0.187 ± 0.0044 | 0.348 ± 0.0043 | 0.36 ± 0.0057 | **0.39** ± 0.0035 |
| **PTBP1_HepG2** | 0.386 ± 0.0026 | 0.508 ± 0.0028 | 0.523 ± 0.0041 | **0.541** ± 0.0028 |
| **SFPQ_HepG2** | 0.072 ± 0.0035 | 0.198 ± 0.0046 | 0.222 ± 0.0076 | **0.266** ± 0.0035 |
| **U2AF2_HepG2** | 0.12 ± 0.0026 | 0.275 ± 0.0066 | 0.286 ± 0.0056 | **0.326** ± 0.0039 |
| **HNRNPL_HepG2** | 0.373 ± 0.0018 | 0.532 ± 0.0035 | 0.539 ± 0.002 | **0.549** ± 0.0013 |
| **HNRNPA1_HepG2** | 0.102 ± 0.0019 | 0.342 ± 0.0029 | 0.349 ± 0.0065 | **0.373** ± 0.0022 |
| **HNRNPC_HepG2** | 0.13 ± 0.0021 | 0.386 ± 0.0023 | 0.394 ± 0.0028 | **0.4** ± 0.0036 |

**C. 19 RBPs on eCLIP data in K562 cell line**

| **RBP** | **Deepbind** | **DLPRB** | **Without_ADDA** | **RBP-ADDA** |
| --- | --- | --- | --- | --- |
| **PCBP1_K562** | 0.222 ± 0.0059 | 0.319 ± 0.0098 | 0.316 ± 0.0149 | **0.399 ± 0.0109** |
| **FMR1_K562** | 0.012 ± 0.0077 | 0.145 ± 0.0068 | 0.167 ± 0.0121 | **0.23 ± 0.0082** |
| **HNRNPK_K562** | 0.199 ± 0.0053 | 0.306 ± 0.0112 | 0.325 ± 0.012 | **0.374 ± 0.005** |
| **PTBP1_K562** | 0.382 ± 0.0029 | 0.443 ± 0.0031 | 0.464 ± 0.0063 | **0.509 ± 0.0041** |
| **SRSF7_K562** | 0.018 ± 0.0057 | 0.182 ± 0.0116 | 0.229 ± 0.0127 | **0.315 ± 0.0079** |
| **PABPC4_K562** | -0.019 ± 0.0057 | 0.109 ± 0.0087 | 0.161 ± 0.012 | **0.272 ± 0.0059** |
| **FXR2_K562** | 0.016 ± 0.0052 | 0.128 ± 0.0062 | 0.142 ± 0.0089 | **0.212 ± 0.0072** |
| **TARDBP_K562** | 0.338 ± 0.005 | 0.441 ± 0.0051 | 0.465 ± 0.0069 | **0.507 ± 0.0041** |
| **MATR3_K562** | 0.158 ± 0.0068 | 0.236 ± 0.0132 | 0.258 ± 0.0146 | **0.342 ± 0.005** |
| **IGF2BP2_K562** | 0.057 ± 0.0046 | 0.139 ± 0.0088 | 0.179 ± 0.0098 | **0.246 ± 0.0061** |
| **SRSF1_K562** | 0.15 ± 0.0037 | 0.242 ± 0.0068 | 0.25 ± 0.0079 | **0.322 ± 0.0047** |
| **FXR1_K562** | 0.095 ± 0.0038 | 0.302 ± 0.0069 | 0.303 ± 0.0066 | **0.352 ± 0.0058** |
| **U2AF2_K562** | 0.096 ± 0.0029 | 0.336 ± 0.0056 | 0.363 ± 0.0084 | **0.398 ± 0.0034** |
| **HNRNPC_K562** | 0.145 ± 0.0036 | 0.296 ± 0.0056 | 0.342 ± 0.0105 | **0.391 ± 0.0086** |
| **FUS_K562** | 0.019 ± 0.0048 | 0.372 ± 0.0071 | 0.39 ± 0.0076 | **0.436 ± 0.0036** |
| **TIA1_K562** | 0.278 ± 0.0029 | 0.368 ± 0.005 | 0.389 ± 0.0056 | **0.416 ± 0.0038** |
| **HNRNPL_K562** | 0.272 ± 0.0028 | 0.386 ± 0.0043 | 0.397 ± 0.0041 | **0.418 ± 0.0029** |
| **HNRNPA1_K562** | 0.09 ± 0.0029 | 0.326 ± 0.0031 | 0.339 ± 0.0062 | **0.369 ± 0.0032** |
| **KHDRBS1_K562** | 0.237 ± 0.0025 | 0.387 ± 0.0027 | 0.397 ± 0.0049 | **0.416 ± 0.0035** |
