# Supplementary material for: Inferring RNA-binding protein target preferences using adversarial domain adaptation
Source: PLoS Comput Biol. 2022 Feb 24;18(2):e1009863. doi: 10.1371/journal.pcbi.1009863 (PMC8870515; doi:10.1371/journal.pcbi.1009863)
Supplement: S3 Table — (DOCX) [file pcbi.1009863.s003.docx]

**Supplemental Table S3.** Summary of results of blastn search between in vivo and in vitro datasets.

We conducted blastn search between the two data sets using Word_Size 16. The number in each cell represents the number of in vitro / in vivo sequence pairs that share a substring of the given length (row) and given range of sequence identity (column). The numbers in the “Sum” column represent the total number of the sequence pairs sharing a matched substring of a specific length and over 80% sequence identity. The numbers in the “Ratio” column correspond to the “Sum” divided by the product of total number of sequences in the in vivo dataset (10,921,666) and in the in vitro dataset (241,357).

| **ID%**  **Length (nt)** | **[80%,85%)** | **[85%,90%)** | **[90%,95%)** | **[95%,100%)** | **100%** | **Sum** | **Ratio** |
| --- | --- | --- | --- | --- | --- | --- | --- |
| **16** | 0 | 0 | 0 | 0 | 245,927 | 245,927 | 9.33E-08 |
| **17** | 0 | 0 | 0 | 0 | 55325 | 55,325 | 2.10E-08 |
| **18** | 0 | 0 | 0 | 0 | 12683 | 12,683 | 4.81E-09 |
| **19** | 0 | 0 | 7,914 | 0 | 2874 | 10,788 | 4.09E-09 |
| **20** | 0 | 0 | 0 | 12,627 | 605 | 13,232 | 5.02E-09 |
| **21** | 0 | 0 | 0 | 5,186 | 117 | 5,303 | 2.01E-09 |
| **22** | 0 | 0 | 743 | 1,776 | 35 | 2,545 | 9.65E-10 |
| **23** | 0 | 0 | 2,291 | 469 | 14 | 2,774 | 1.05E-09 |
| **24** | 0 | 0 | 1,408 | 119 | 0 | 1,527 | 5.79E-10 |
| **25** | 0 | 87 | 572 | 34 | 0 | 693 | 2.63E-10 |
| **26** | 0 | 419 | 164 | 4 | 0 | 587 | 2.23E-10 |
| **27** | 0 | 279 | 56 | 4 | 0 | 339 | 1.29E-10 |
| **28** | 0 | 130 | 25 | 0 | 0 | 155 | 5.88E-11 |
| **29** | 0 | 113 | 2 | 0 | 0 | 115 | 4.36E-11 |
| **30** | 0 | 59 | 9 | 0 | 0 | 68 | 2.58E-11 |
| **31** | 0 | 33 | 1 | 0 | 0 | 34 | 1.29E-11 |
| **32** | 12 | 15 | 2 | 0 | 0 | 29 | 1.10E-11 |
| **33** | 18 | 4 | 0 | 0 | 0 | 22 | 8.35E-12 |
| **34** | 0 | 13 | 0 | 0 | 0 | 13 | 4.93E-12 |
| **35** | 1 | 4 | 0 | 0 | 0 | 5 | 1.90E-12 |
| **36** | 1 | 2 | 0 | 0 | 0 | 3 | 1.14E-12 |
| **37** | 1 | 0 | 0 | 0 | 0 | 1 | 3.79E-13 |
| **38** | 0 | 0 | 0 | 0 | 0 | 0 | 0.00E+00 |
| **39** | 0 | 0 | 0 | 0 | 0 | 0 | 0.00E+00 |
| **40** | 1 | 0 | 0 | 0 | 0 | 1 | 3.79E-13 |
