# Supplementary material for: Inferring RNA-binding protein target preferences using adversarial domain adaptation
Source: PLoS Comput Biol. 2022 Feb 24;18(2):e1009863. doi: 10.1371/journal.pcbi.1009863 (PMC8870515; doi:10.1371/journal.pcbi.1009863)
Supplement: S4 Table — (DOCX) [file pcbi.1009863.s004.docx]

**Supplemental Table S4.** Runtime of different methods. We trained and tested each method on a PC with Intel Core i5-6200 8G RAM. For RBP-ADDA, we further report the training time for each step. For DLPRB, we report the train and test time. Since Deepbind only provides a well-trained model, we only report the test time.

|  | **RBP-ADDA** | | **DLPRB** | **Deepbind** |
| --- | --- | --- | --- | --- |
| Training time  (each epoch) | Source Pre-training | 9.2 s | 47.7 s | - |
|  | Domain Adaptation | 22.7 s |  |  |
|  | Model Fine-tuneing | 8.2 s |  |  |
| Testing time | 1.7 s | | 6.5 s | 25.5 s |
