# Supplementary material for: Inferring RNA-binding protein target preferences using adversarial domain adaptation
Source: PLoS Comput Biol. 2022 Feb 24;18(2):e1009863. doi: 10.1371/journal.pcbi.1009863 (PMC8870515; doi:10.1371/journal.pcbi.1009863)
Supplement: S5 Table — (DOCX) [file pcbi.1009863.s005.docx]

**Supplemental S5**

Detailed comparisons between RBP-ADDA and the “Reversed model”. The Reversed model is trained with in vivo data as source domain and in vitro data as target domain. Table S5A compares the results for in vitro RNAcompete data while Table S5B and Table S5C compare the results for in vivo data derived from different cell lines.

| 1. **In vitro data (RNAcompete)** | | | 1. **In vivo data (HepG2 cell line)** | | | 1. **In vivo data (K562 cell line)** | | |
| --- | --- | --- | --- | --- | --- | --- | --- | --- |
| **RBP** | **RBP-ADDA** | **Reverse** | **RBP** | **RBP-ADDA** | **Reverse** | **RBP** | **RBP-ADDA** | **Reverse** |
| PCBP1 | 0.418 | **0.422** | PCBP1_HepG2 | 0.521 | **0.536** | PCBP1_K562 | 0.399 | **0.405** |
| FMR1 | **0.752** | 0.748 | FXR2_HepG2 | **0.234** | 0.231 | FMR1_K562 | 0.23 | **0.233** |
| HNRNPK | **0.652** | 0.651 | SRSF7_HepG2 | 0.414 | 0.414 | HNRNPK_K562 | **0.374** | 0.368 |
| PTBP1 | **0.837** | 0.835 | PABPN1_HepG2 | **0.226** | 0.216 | PTBP1_K562 | **0.509** | 0.505 |
| SRSF7 | **0.708** | 0.707 | SRSF9_HepG2 | **0.307** | 0.303 | SRSF7_K562 | **0.315** | 0.309 |
| PABPC4 | **0.832** | 0.824 | IGF2BP3_HepG2 | **0.317** | 0.311 | PABPC4_K562 | **0.272** | 0.263 |
| FXR2 | 0.469 | **0.471** | PCBP2_HepG2 | **0.412** | 0.406 | FXR2_K562 | **0.212** | 0.209 |
| TARDBP | **0.763** | 0.756 | HNRNPK_HepG2 | **0.495** | 0.493 | TARDBP_K562 | **0.507** | 0.502 |
| MATR3 | **0.764** | 0.761 | TIA1_HepG2 | **0.383** | 0.38 | MATR3_K562 | **0.342** | 0.34 |
| IGF2BP2 | **0.613** | 0.612 | RBM5_HepG2 | 0.259 | **0.261** | IGF2BP2_K562 | **0.246** | 0.241 |
| SRSF1 | **0.723** | 0.718 | SRSF1_HepG2 | **0.373** | 0.371 | SRSF1_K562 | **0.322** | 0.314 |
| FXR1 | 0.649 | 0.649 | FUS_HepG2 | 0.25 | **0.253** | FXR1_K562 | 0.352 | **0.353** |
| U2AF2 | **0.625** | 0.621 | MATR3_HepG2 | 0.39 | 0.39 | U2AF2_K562 | **0.398** | 0.395 |
| HNRNPC | **0.679** | 0.677 | PTBP1_HepG2 | 0.541 | **0.542** | HNRNPC_K562 | 0.391 | **0.396** |
| FUS | **0.639** | 0.63 | SFPQ_HepG2 | **0.266** | 0.265 | FUS_K562 | 0.436 | **0.438** |
| TIA1 | **0.828** | 0.821 | U2AF2_HepG2 | **0.326** | 0.323 | TIA1_K562 | 0.416 | **0.417** |
| HNRNPL | **0.798** | 0.794 | HNRNPL_HepG2 | 0.549 | **0.551** | HNRNPL_K562 | 0.418 | 0.418 |
| HNRNPA1 | **0.84** | 0.835 | HNRNPA1_HepG2 | **0.373** | 0.371 | HNRNPA1_K562 | 0.369 | 0.369 |
| KHDRBS1 | 0.775 | **0.776** | HNRNPC_HepG2 | 0.4 | **0.404** | KHDRBS1_K562 | **0.416** | 0.414 |
| PABPN1 | **0.613** | 0.607 |  |  |  |  |  |  |
| SRSF9 | **0.527** | 0.525 |  |  |  |  |  |  |
| IGF2BP3 | **0.626** | 0.62 |  |  |  |  |  |  |
| PCBP2 | 0.211 | **0.22** |  |  |  |  |  |  |
| RBM5 | **0.71** | 0.709 |  |  |  |  |  |  |
| SFPQ | **0.828** | 0.822 |  |  |  |  |  |  |
