# Supplementary figures and images for: Inferring RNA-binding protein target preferences using adversarial domain adaptation
Source: PLoS Comput Biol. 2022 Feb 24;18(2):e1009863. doi: 10.1371/journal.pcbi.1009863 (PMC8870515; doi:10.1371/journal.pcbi.1009863)

Fig S1

**A. Source/Target Network**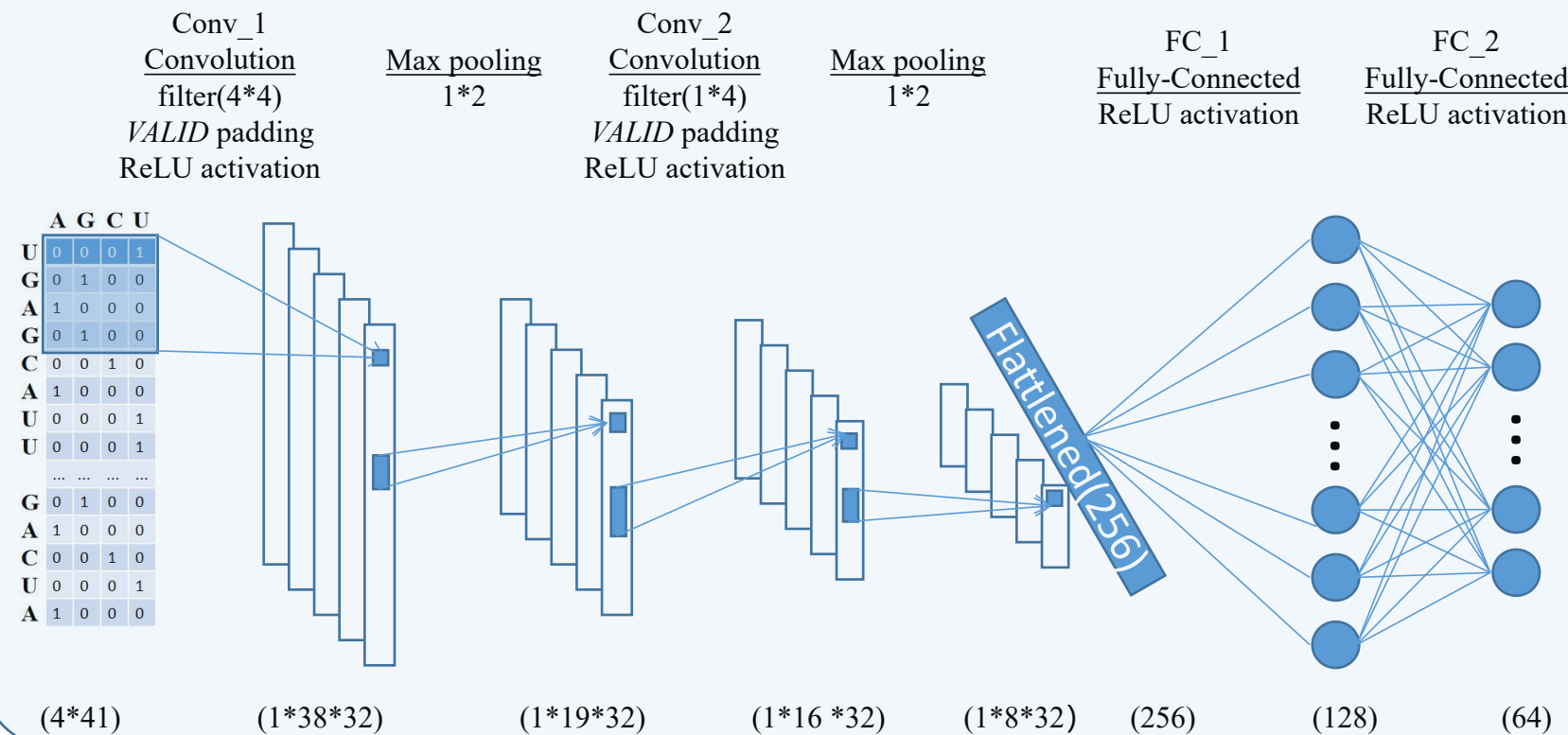**B. Task Predictor**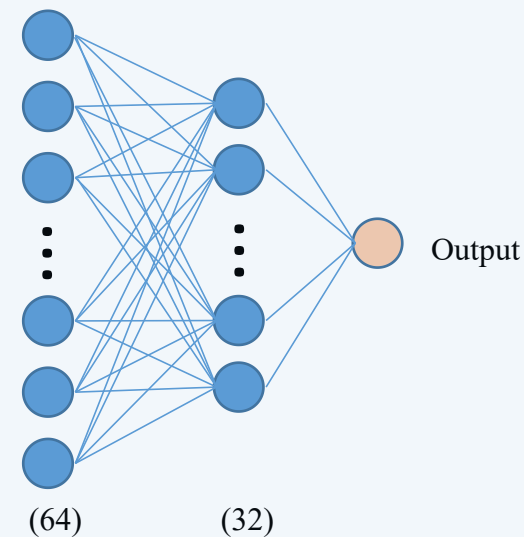**C. Discriminator**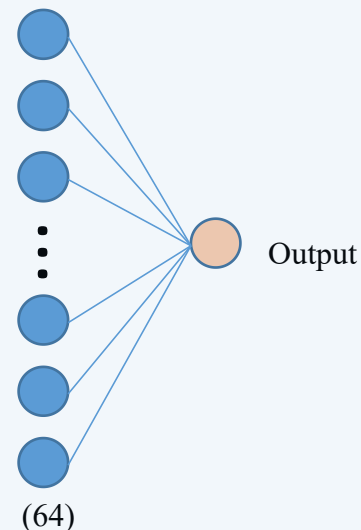

Supplement: S1 Fig — (A). The architecture of Source and Target Network. (B). The architecture of the Task Predictor. (C). The architecture of the Discriminator. (PDF) [file pcbi.1009863.s006.pdf]

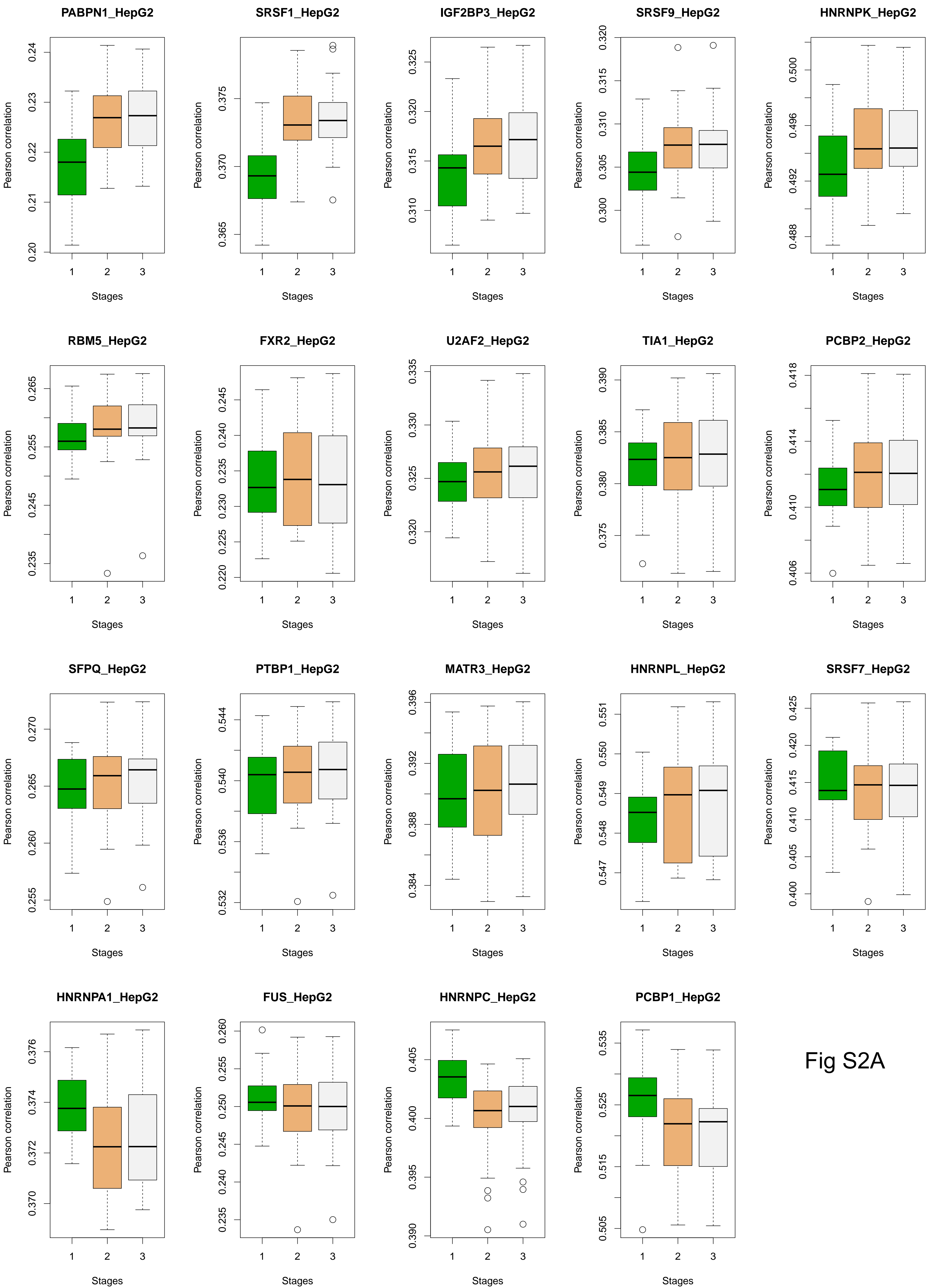

Fig S2A

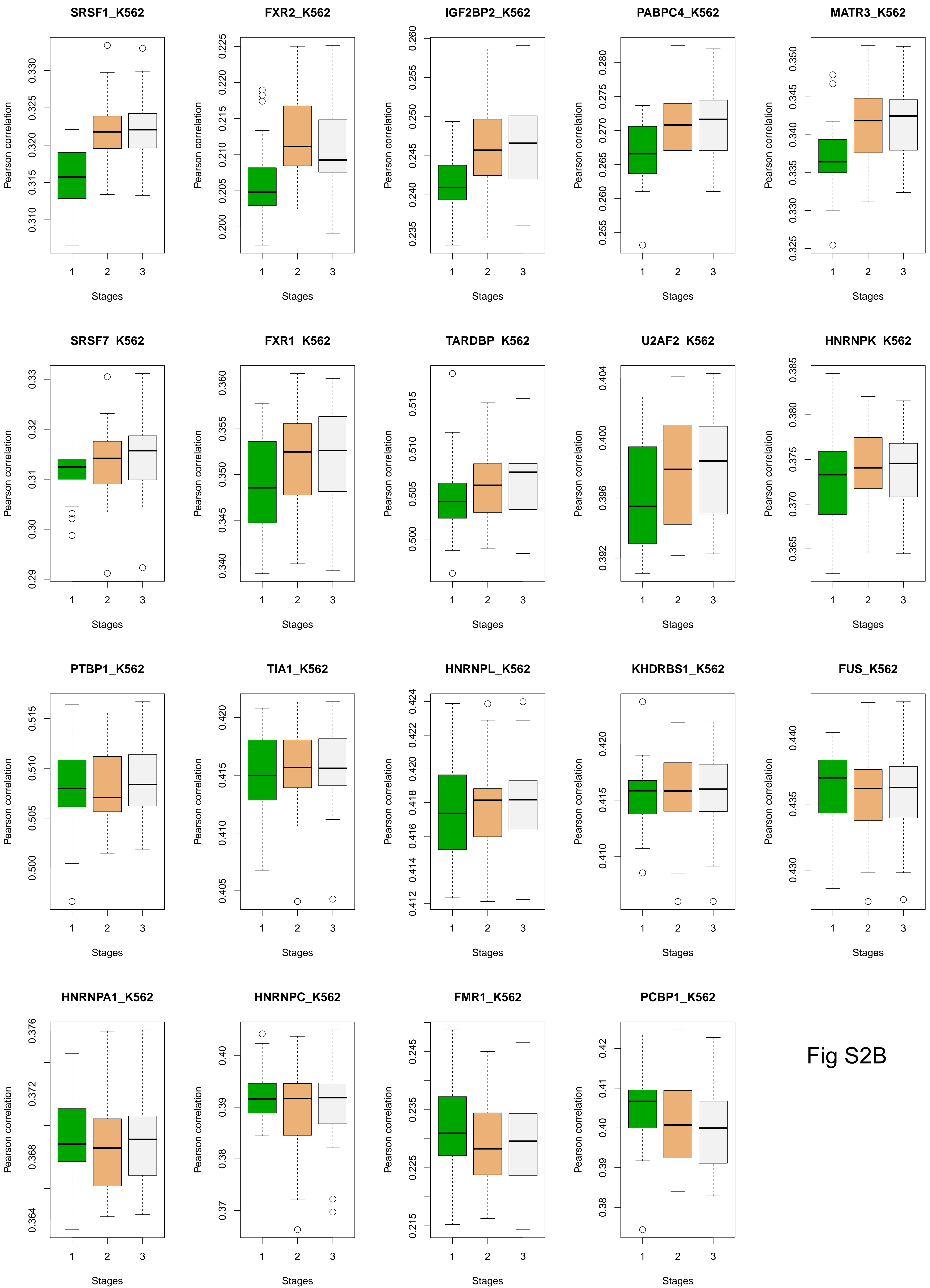

Fig S2B

Fig S2C

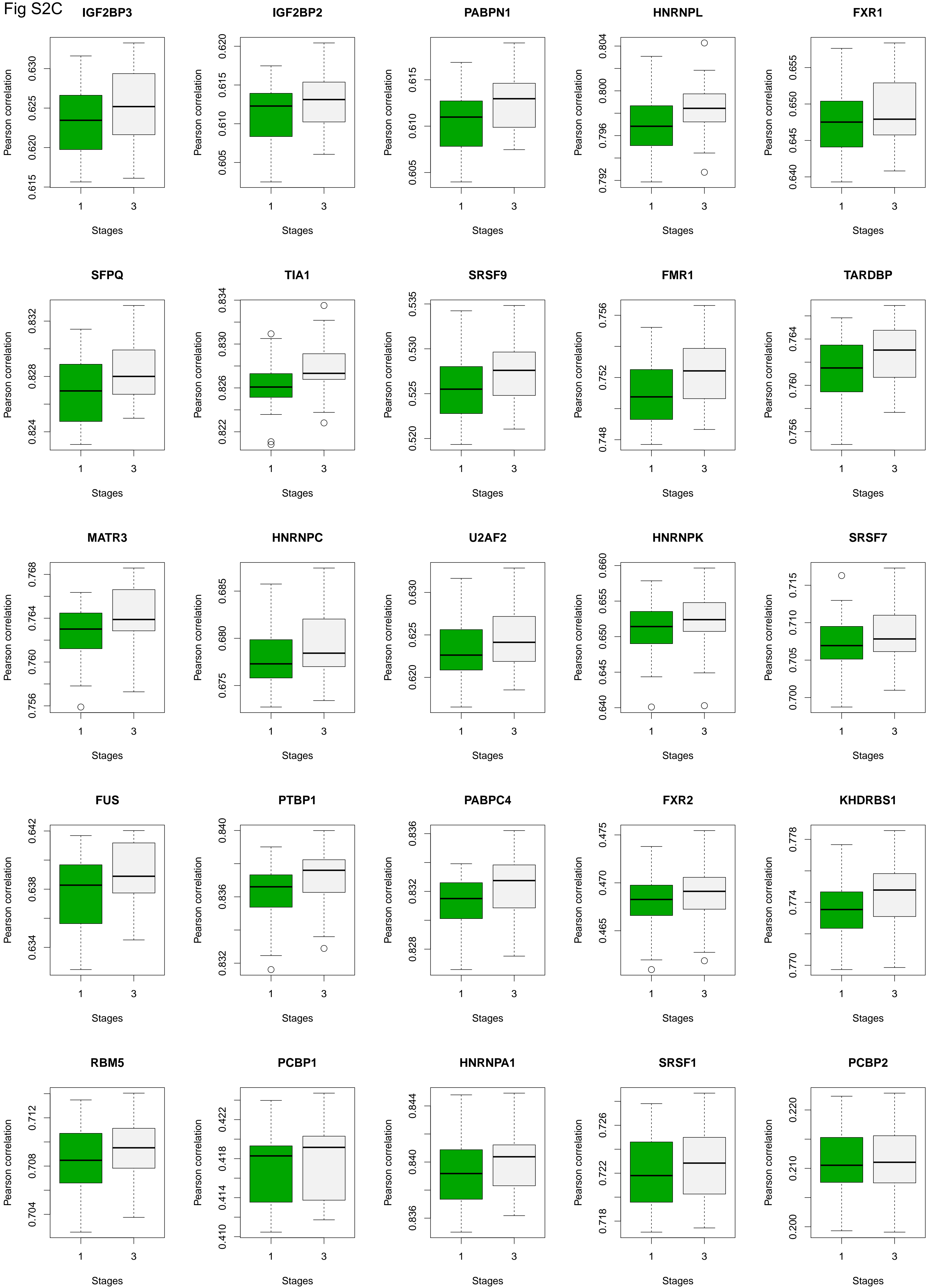

Supplement: S2 Fig — “1”, “2”, and “3” represent the pre-training, domain adaptation, and model fine-tuning steps respectively. For source data, we update the source model only in pre-training and fine-tuning. (A) Performance at different steps on target in vivo data tested in HepG2 cell lines. (B) Performance on target in vivo data tested in K562 cell line. (C) Performance of tested on source in vitro data. (PDF) [file pcbi.1009863.s007.pdf]

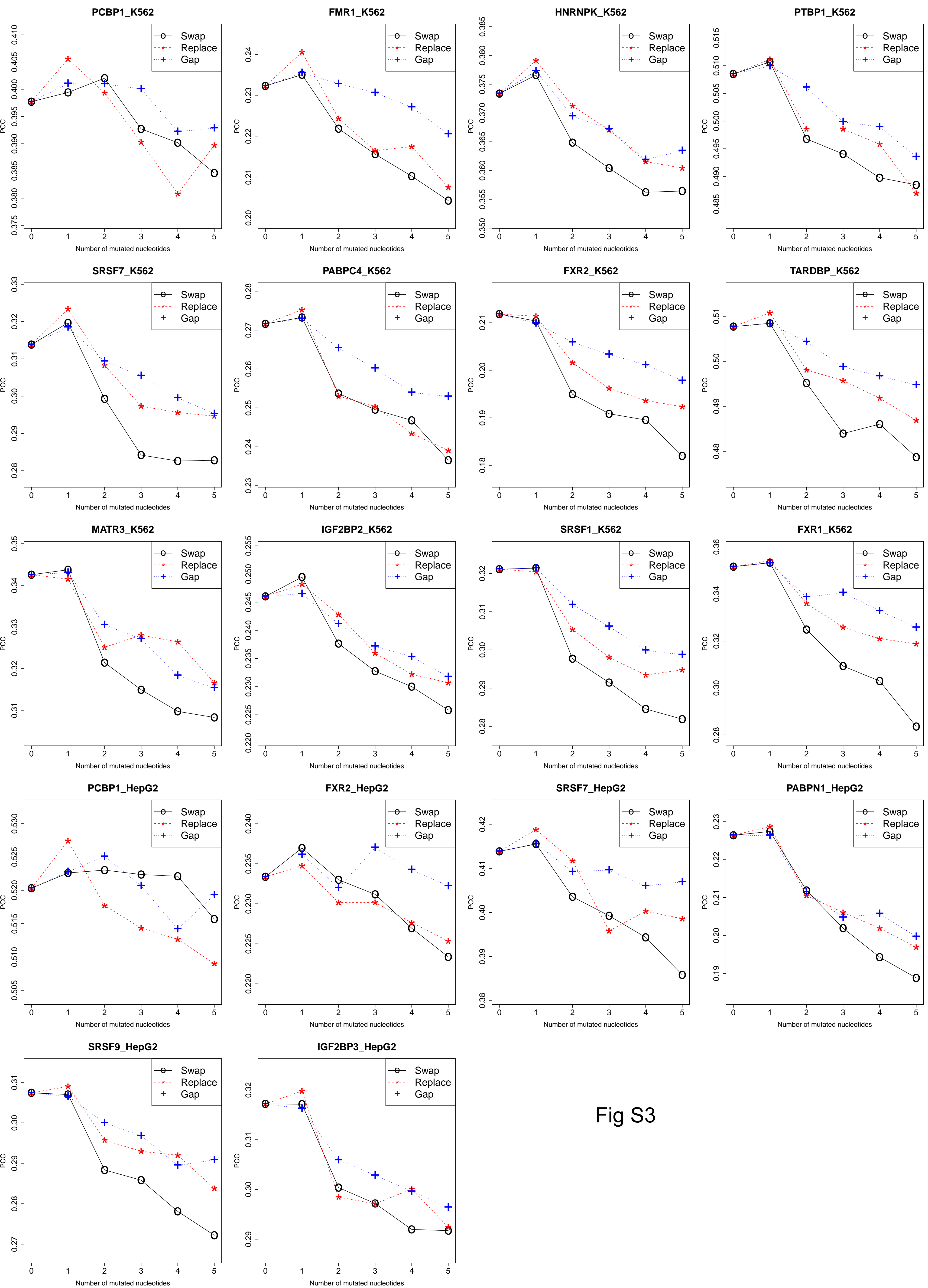

Fig S3

Supplement: S3 Fig — (PDF) [file pcbi.1009863.s008.pdf]

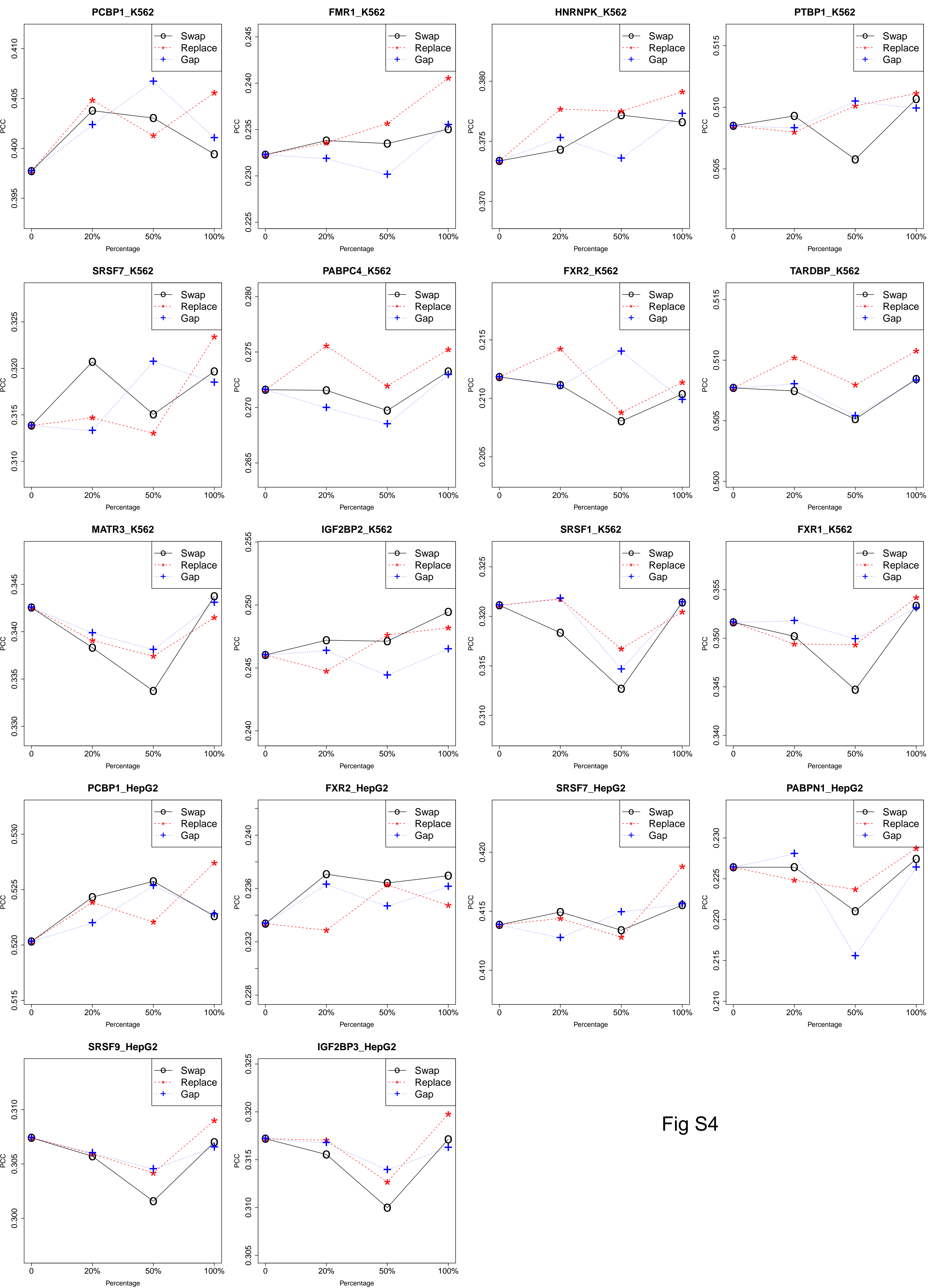

Fig S4

Supplement: S4 Fig — Only single nucleotide augmentation is applied and shown here. (PDF) [file pcbi.1009863.s009.pdf]
