# Supplementary material for: Inferring RNA-binding protein target preferences using adversarial domain adaptation
Source: PLoS Comput Biol. 2022 Feb 24;18(2):e1009863. doi: 10.1371/journal.pcbi.1009863 (PMC8870515; doi:10.1371/journal.pcbi.1009863)
Supplement: S5 Fig — For each RBP, the top 5 RNA fragments are visualized with attribution scores calculated following integrated gradients (IG) method. (PDF) [file pcbi.1009863.s010.pdf]

Fig S5

# Contents


| | | | |
|---|---|---|---|
| FMR1_K562 | 2 | FUS_HepG2 | 21 |
| FUS_K562 | 3 | FXR2_HepG2 | 22 |
| FXR1_K562 | 4 | HNRNPC_HepG2 | 23 |
| FXR2_K562 | 5 | HNRNPK_HepG2 | 24 |
| HNRNPA1_K562 | 6 | HNRNPL_HepG2 | 25 |
| HNRNPC_K562 | 7 | IGF2BP3_HepG2 | 26 |
| HNRNPK_K562 | 8 | MATR3_HepG2 | 27 |
| HNRNPL_K562 | 9 | PABPN1_HepG2 | 28 |
| IGF2BP2_K562 | 10 | PCBP1_HepG2 | 29 |
| KHDRBS1_K562 | 11 | PCBP2_HepG2 | 30 |
| MATR3_K562 | 12 | PTBP1_HepG2 | 31 |
| PABPC4_K562 | 13 | RBM5_HepG2 | 32 |
| PCBP1_K562 | 14 | SFPQ_HepG2 | 33 |
| PTBP1_K562 | 15 | SRSF1_HepG2 | 34 |
| SRSF1_K562 | 16 | SRSF7_HepG2 | 35 |
| SRSF7_K562 | 17 | SRSF9_HepG2 | 36 |
| TARDBP_K562 | 18 | TIA1_HepG2 | 37 |
| TIA1_K562 | 19 | U2AF2_HepG2 | 38 |
| U2AF2_K562 | 20 | | |

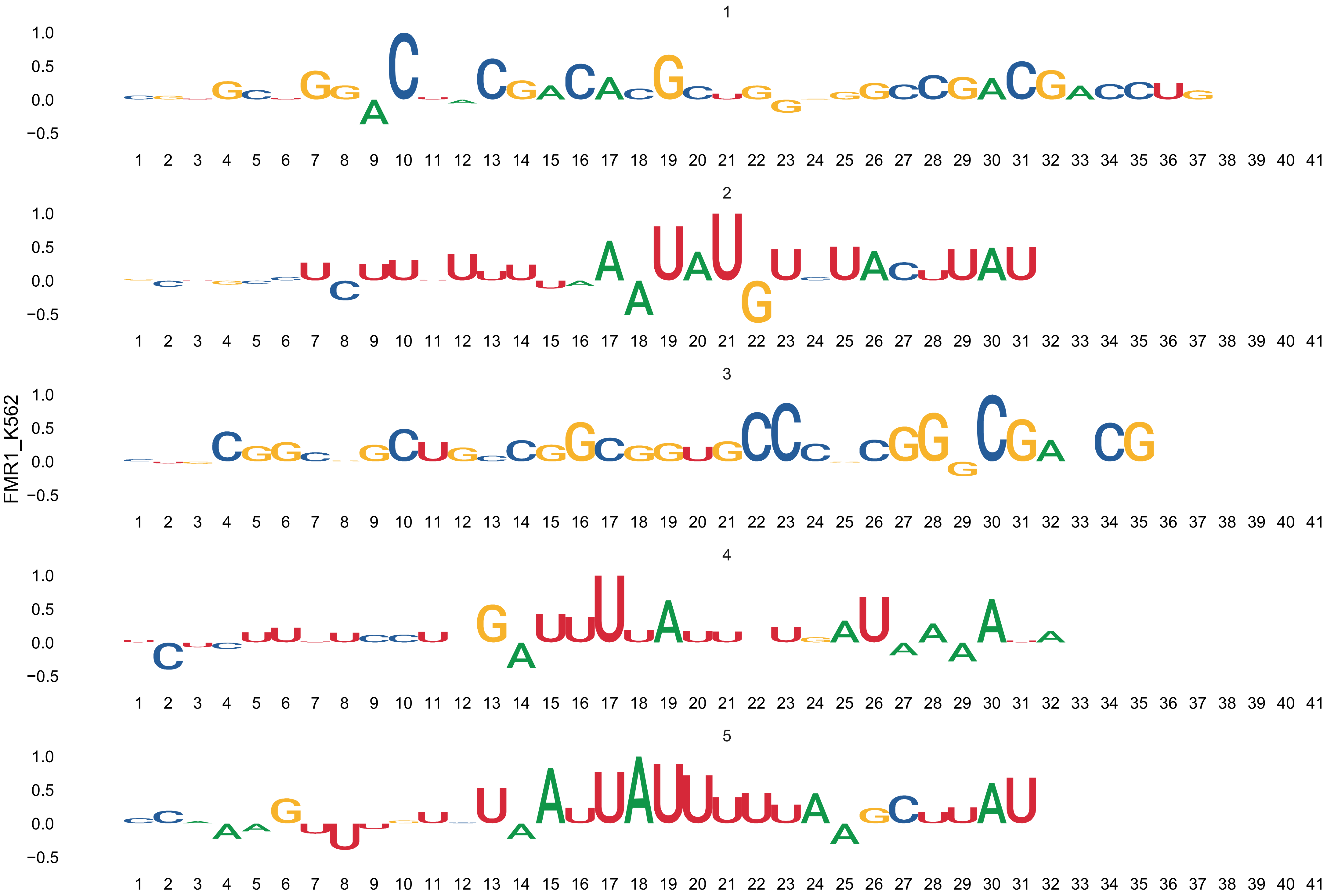
FMR1_K562
1
2
3
4
5
1.0
0.5
0.0
−0.5
1 2 3 4 5 6 7 8 9 10 11 12 13 14 15 16 17 18 19 20 21 22 23 24 25 26 27 28 29 30 31 32 33 34 35 36 37 38 39 40 41

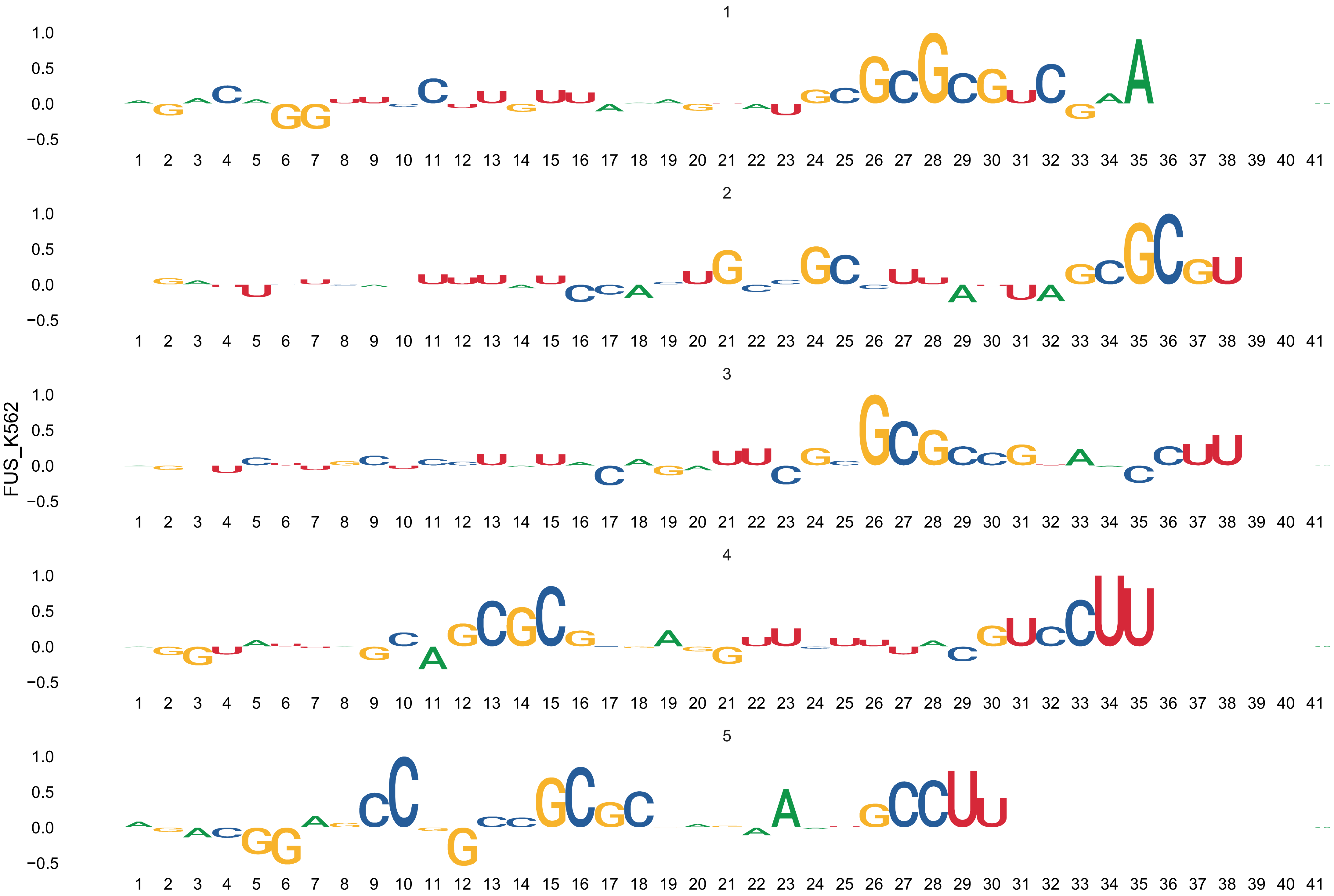
FUS_K562
1
2
3
4
5
1.0
0.5
0.0
−0.5
1 2 3 4 5 6 7 8 9 10 11 12 13 14 15 16 17 18 19 20 21 22 23 24 25 26 27 28 29 30 31 32 33 34 35 36 37 38 39 40 41

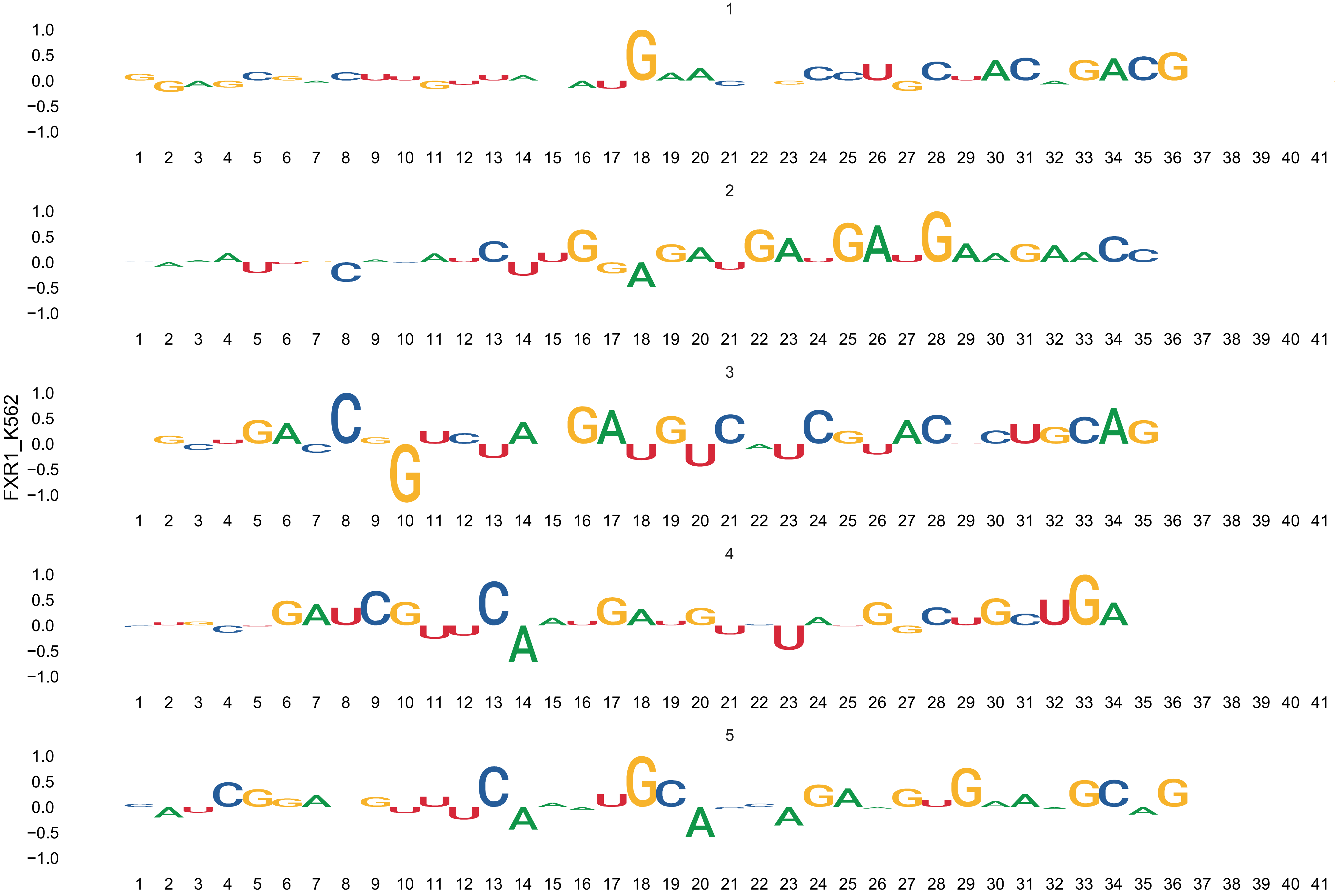
FXR1_K562
1
2
3
4
5
1.0
0.5
0.0
−0.5
−1.0

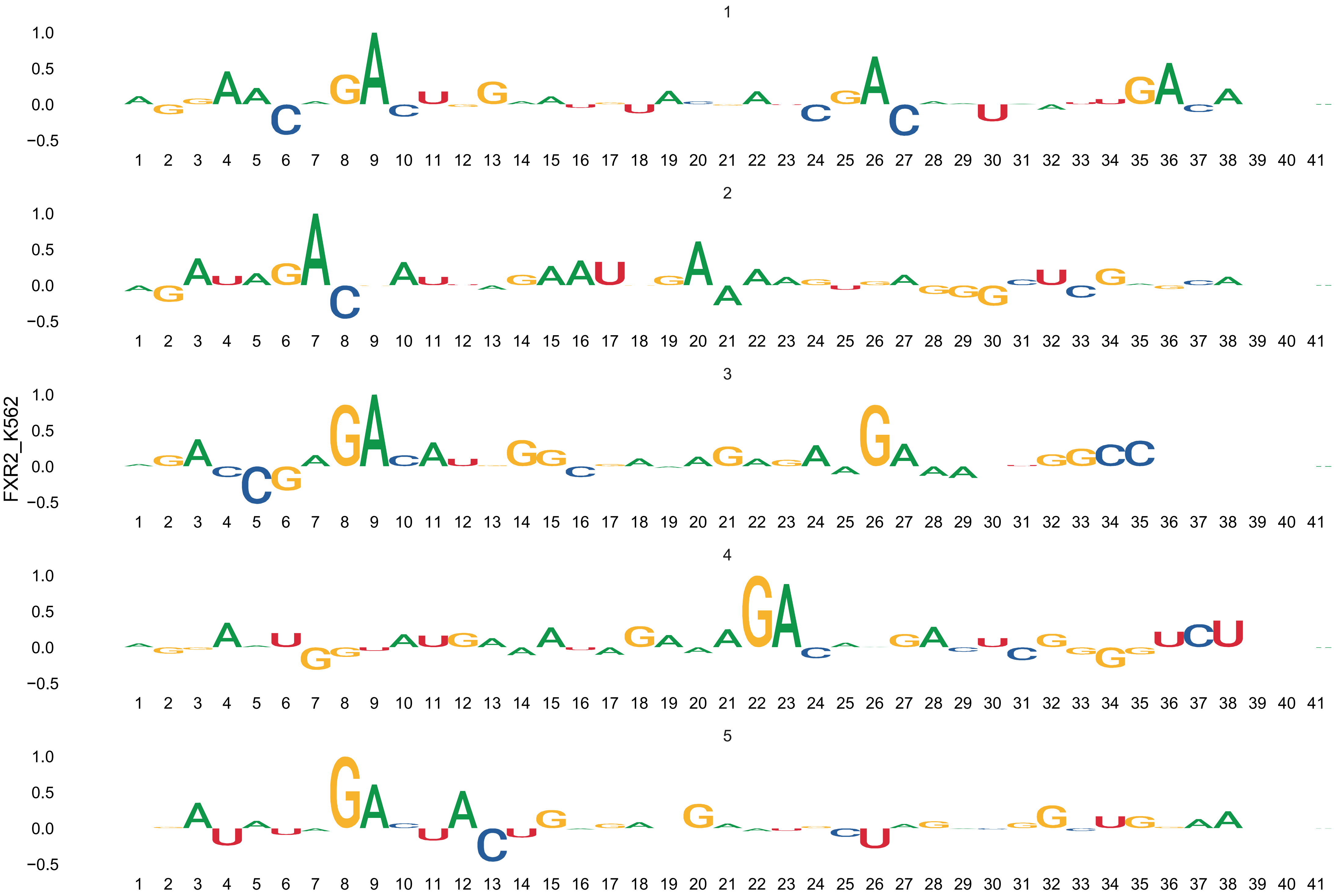
FXR2_K562
1
2
3
4
5
1.0
0.5
0.0
−0.5

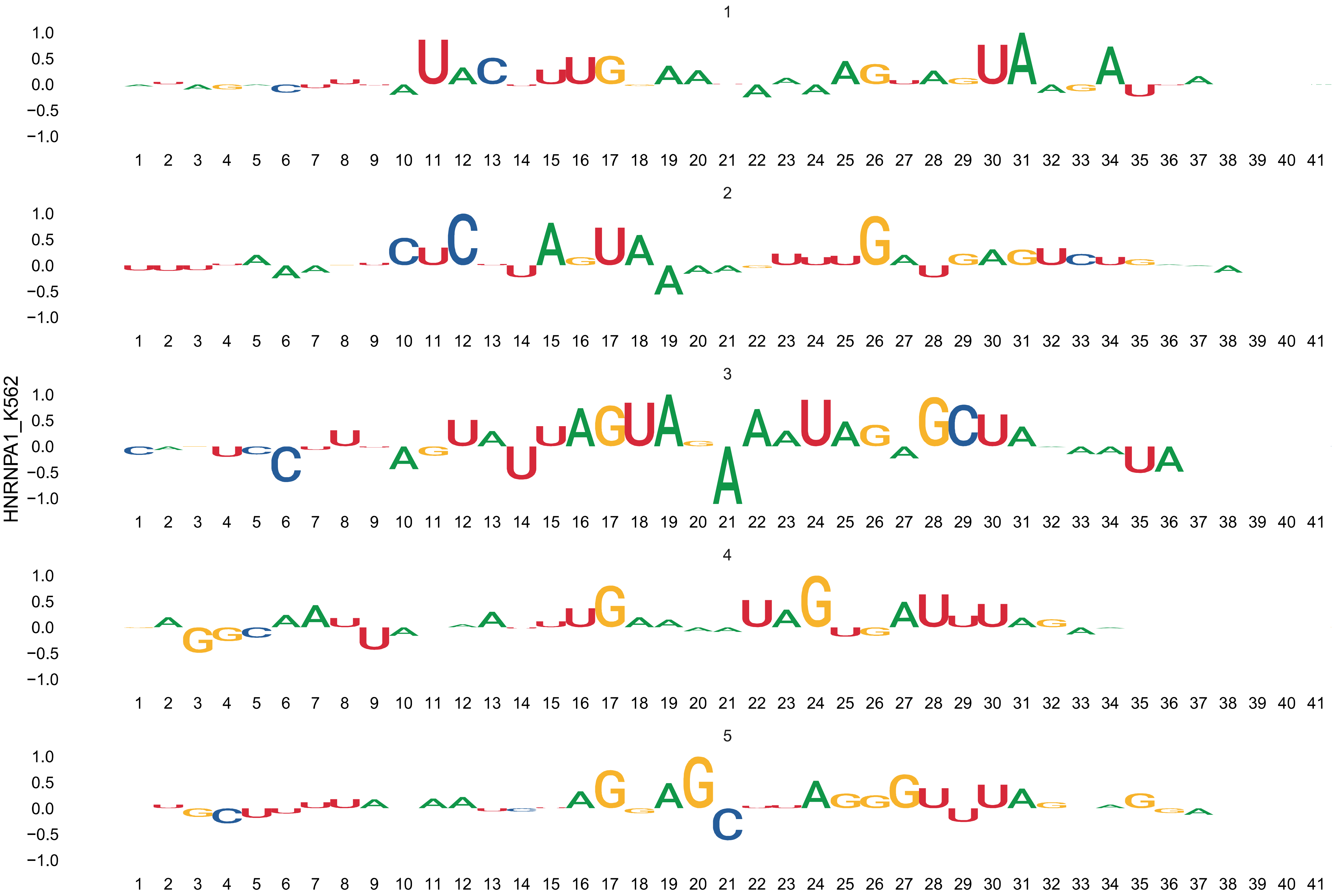
HNRNPA1_K562
1
2
3
4
5
1.0
0.5
0.0
−0.5
−1.0
1 2 3 4 5 6 7 8 9 10 11 12 13 14 15 16 17 18 19 20 21 22 23 24 25 26 27 28 29 30 31 32 33 34 35 36 37 38 39 40 41

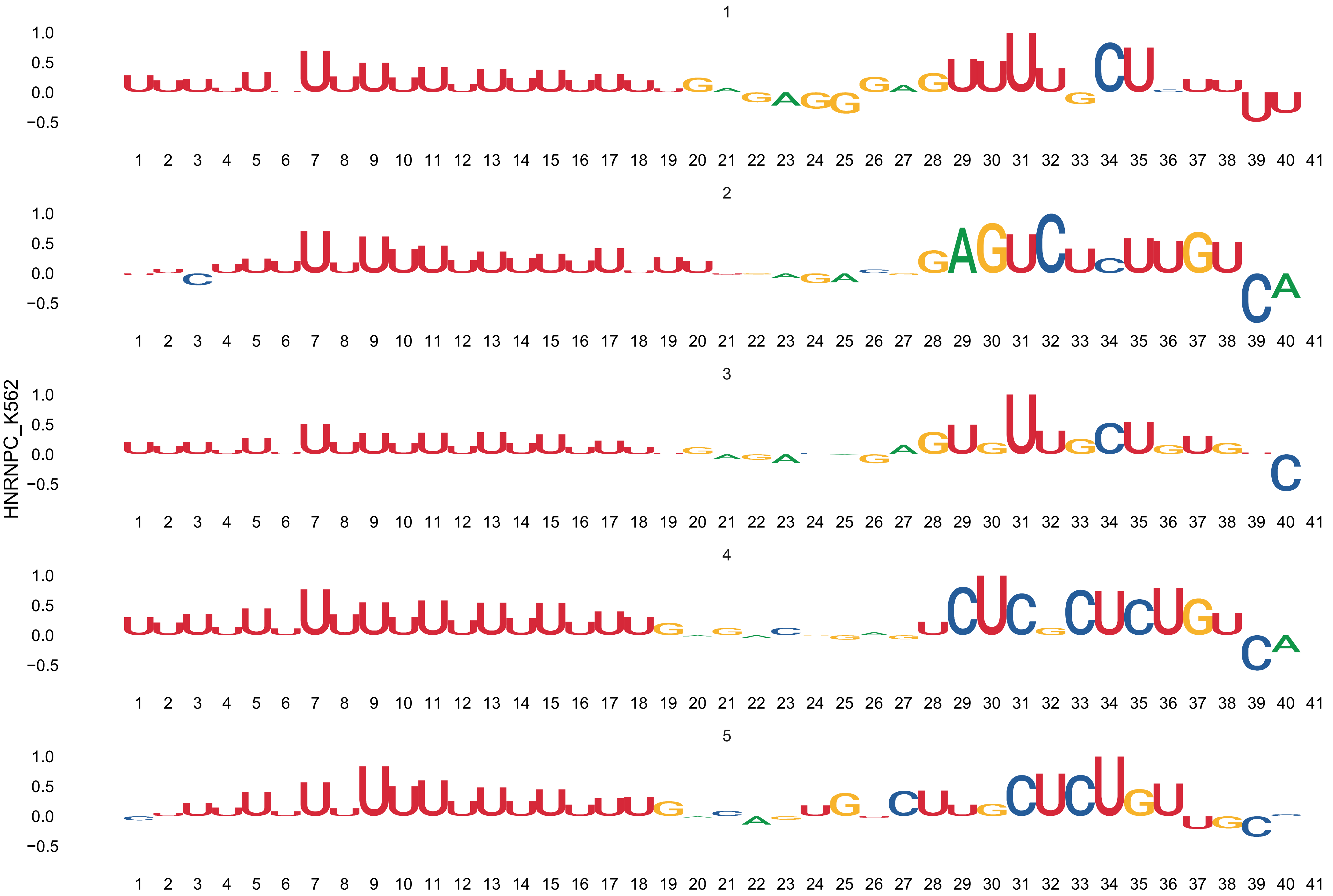
HNRNPC_K562
1
2
3
4
5
1.0
0.5
0.0
−0.5
1 2 3 4 5 6 7 8 9 10 11 12 13 14 15 16 17 18 19 20 21 22 23 24 25 26 27 28 29 30 31 32 33 34 35 36 37 38 39 40 41

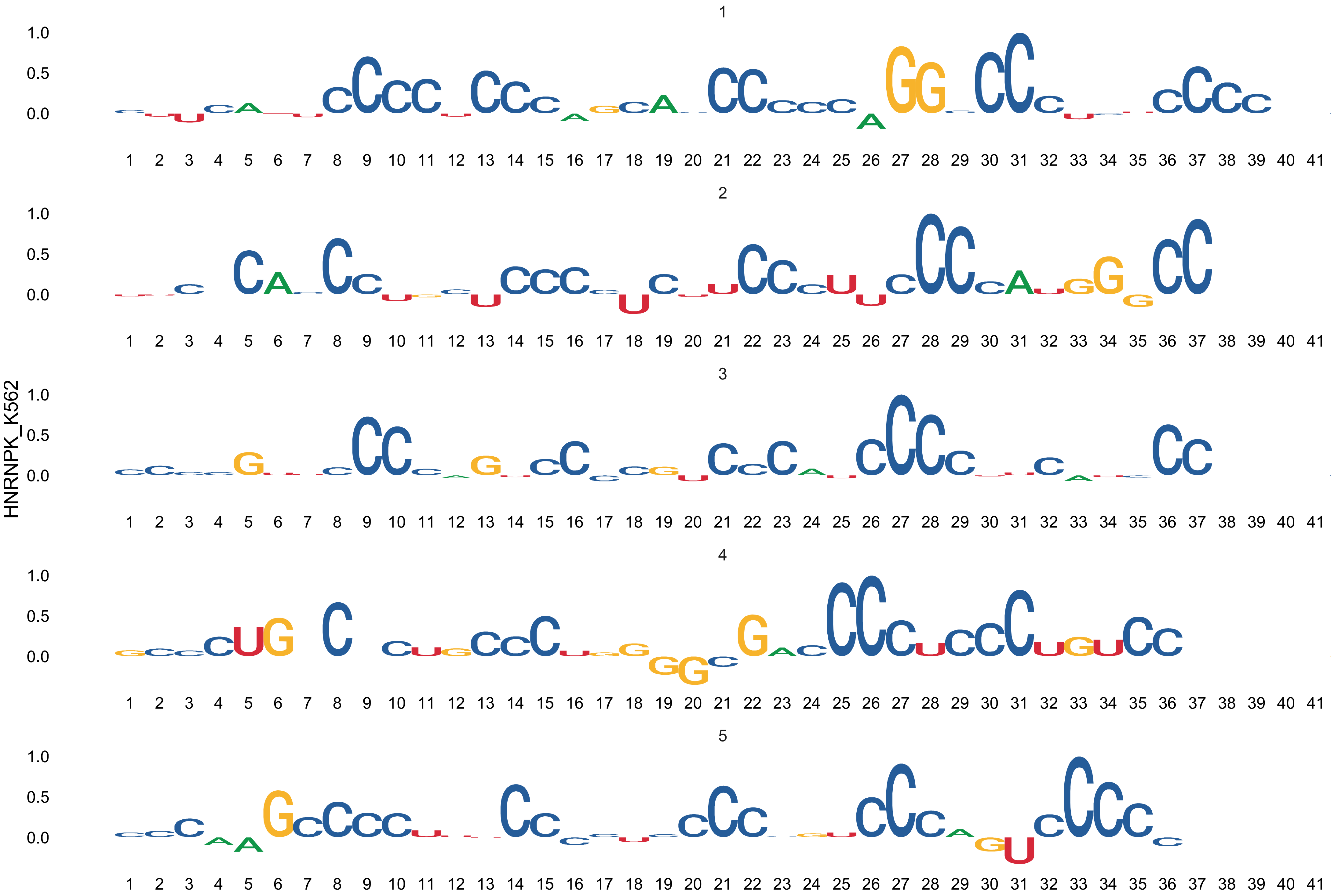
HNRNPK_K562
1
2
3
4
5
1.0
0.5
0.0
1 2 3 4 5 6 7 8 9 10 11 12 13 14 15 16 17 18 19 20 21 22 23 24 25 26 27 28 29 30 31 32 33 34 35 36 37 38 39 40 41

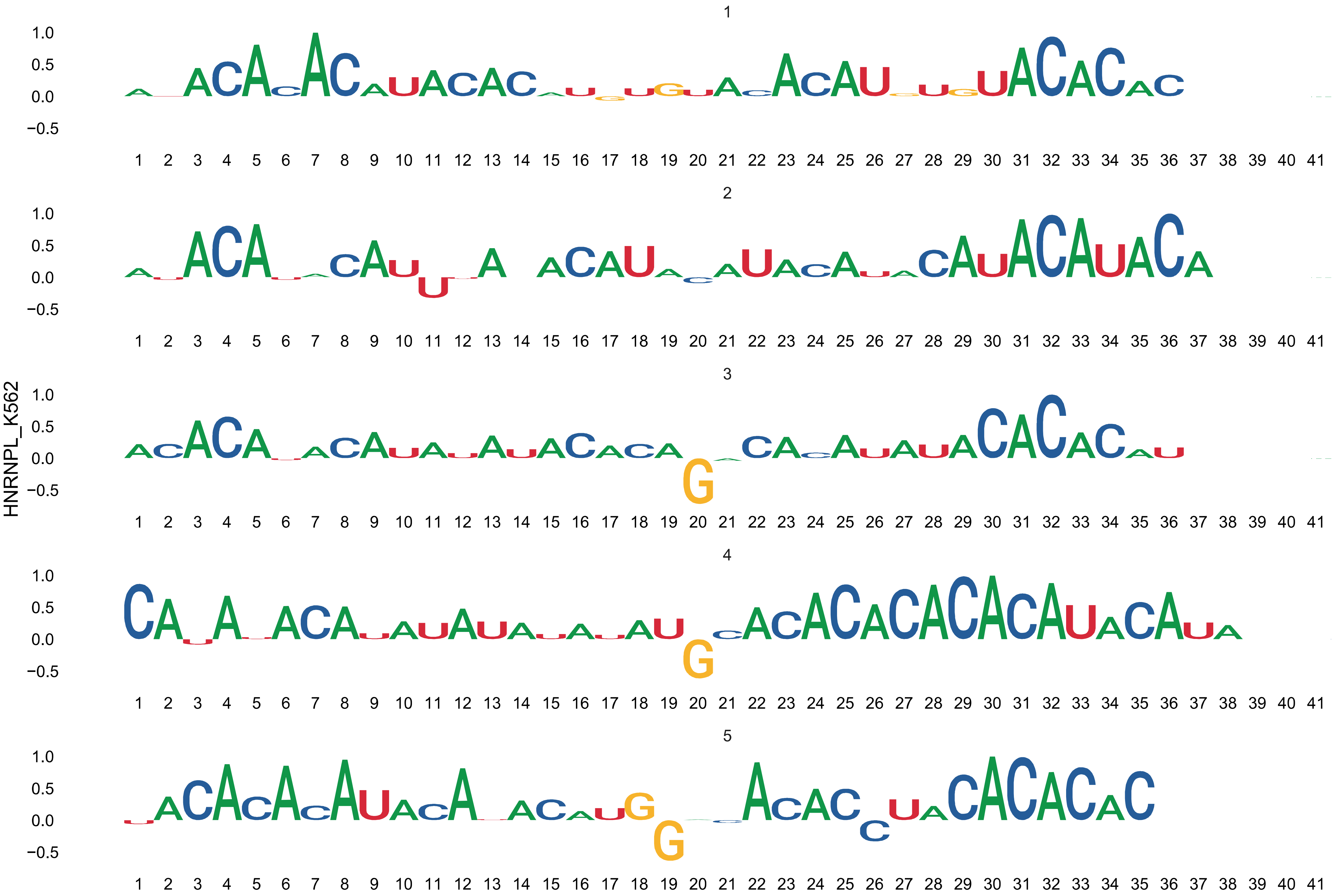

HNRNPL_K562
1
2
3
4
5
1.0
0.5
0.0
−0.5
1 2 3 4 5 6 7 8 9 10 11 12 13 14 15 16 17 18 19 20 21 22 23 24 25 26 27 28 29 30 31 32 33 34 35 36 37 38 39 40 41

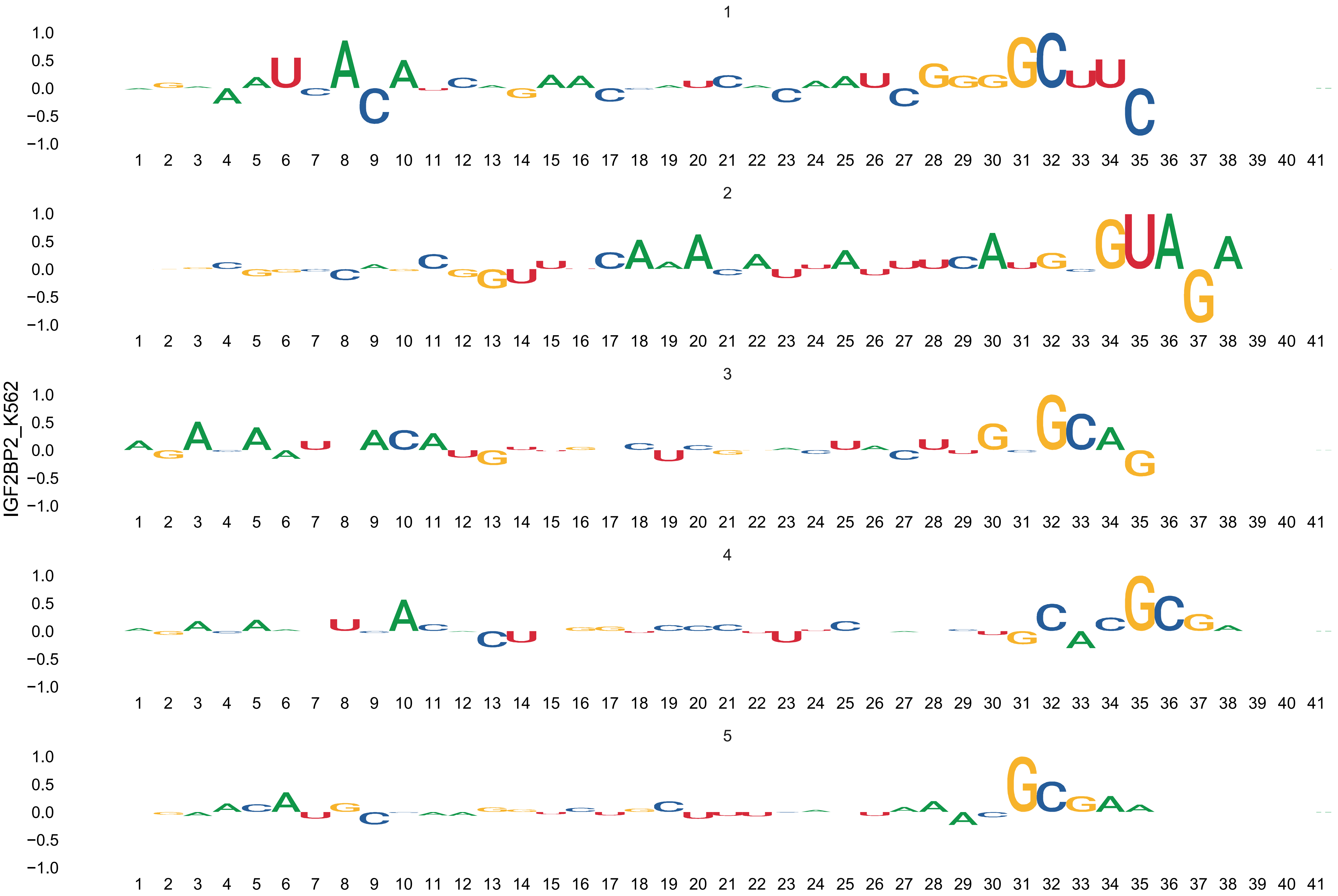
1
2
3
4
5
IGF2BP2_K562

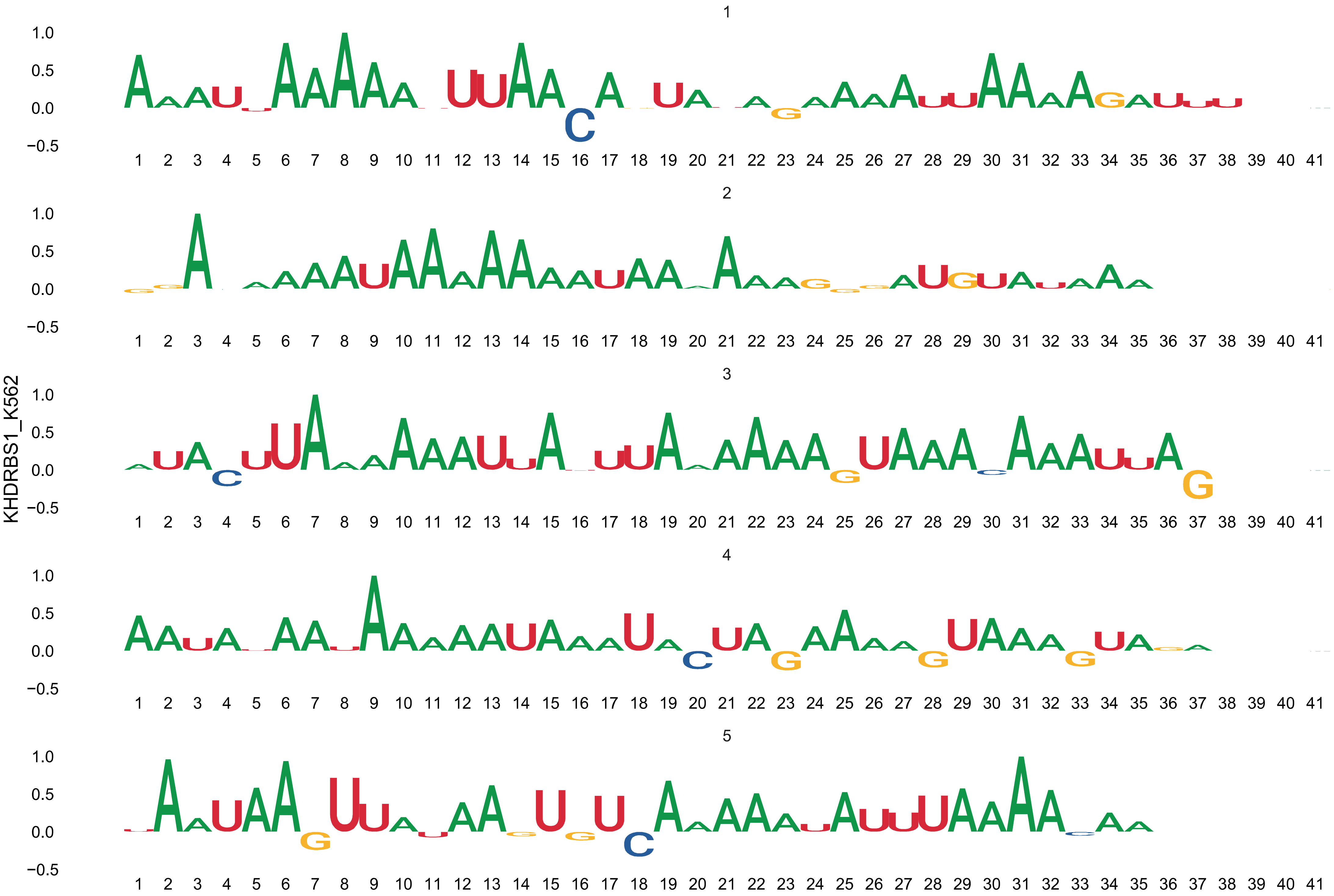
KHDRBS1_K562
1
2
3
4
5
1.0
0.5
0.0
−0.5
1 2 3 4 5 6 7 8 9 10 11 12 13 14 15 16 17 18 19 20 21 22 23 24 25 26 27 28 29 30 31 32 33 34 35 36 37 38 39 40 41

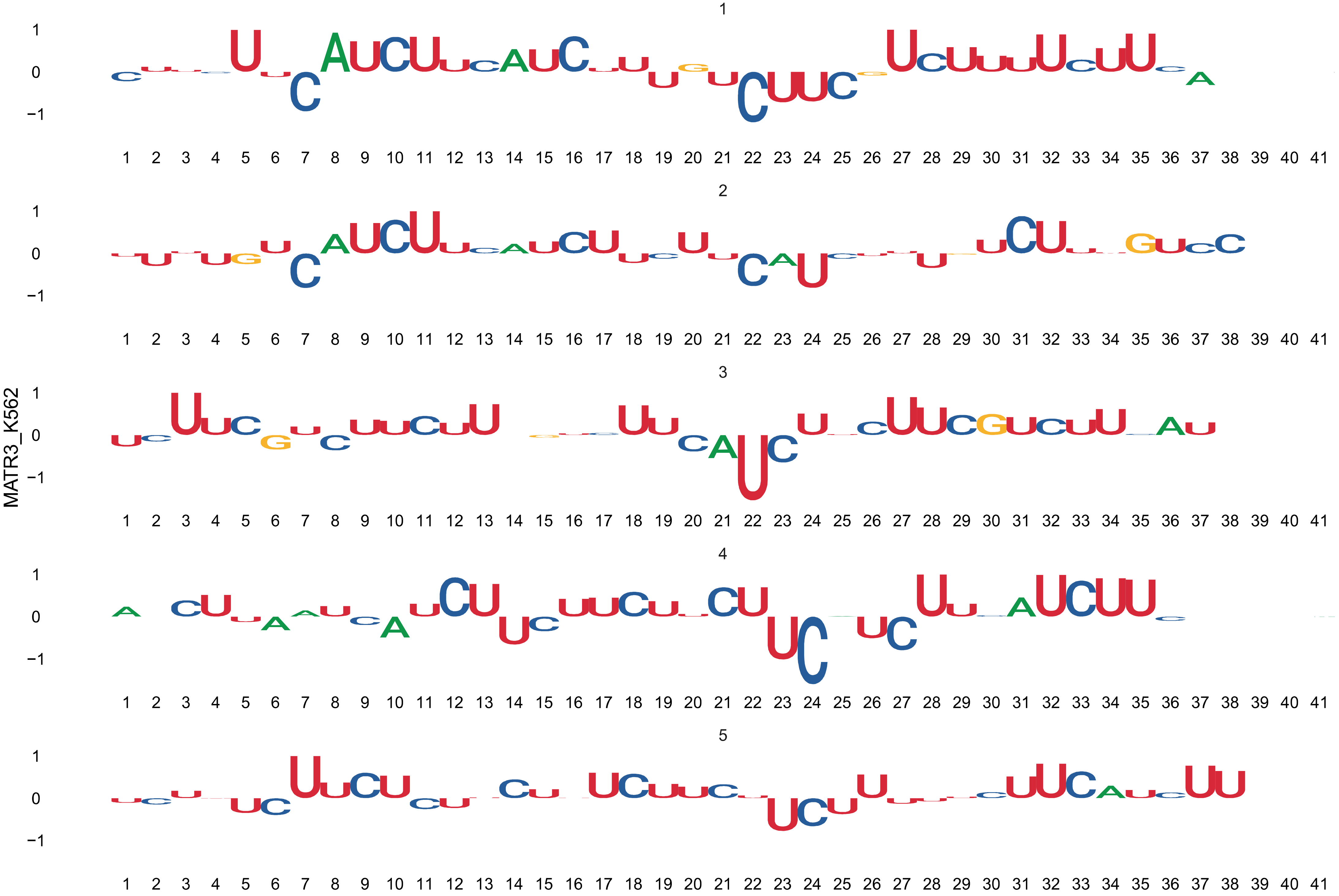
MATR3_K562
1
2
3
4
5

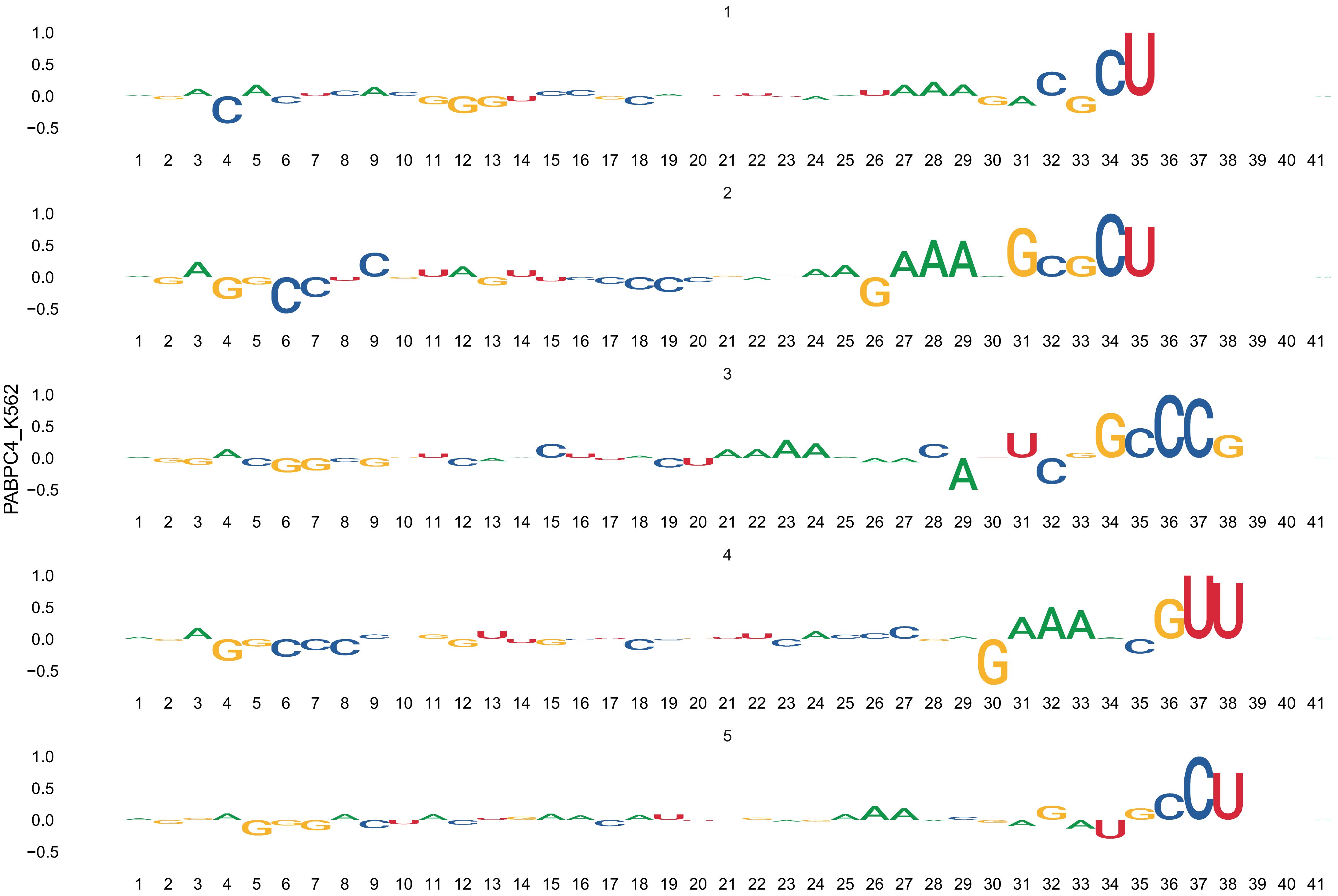
PABPC4_K562
1
2
3
4
5
1.0
0.5
0.0
−0.5
1 2 3 4 5 6 7 8 9 10 11 12 13 14 15 16 17 18 19 20 21 22 23 24 25 26 27 28 29 30 31 32 33 34 35 36 37 38 39 40 41

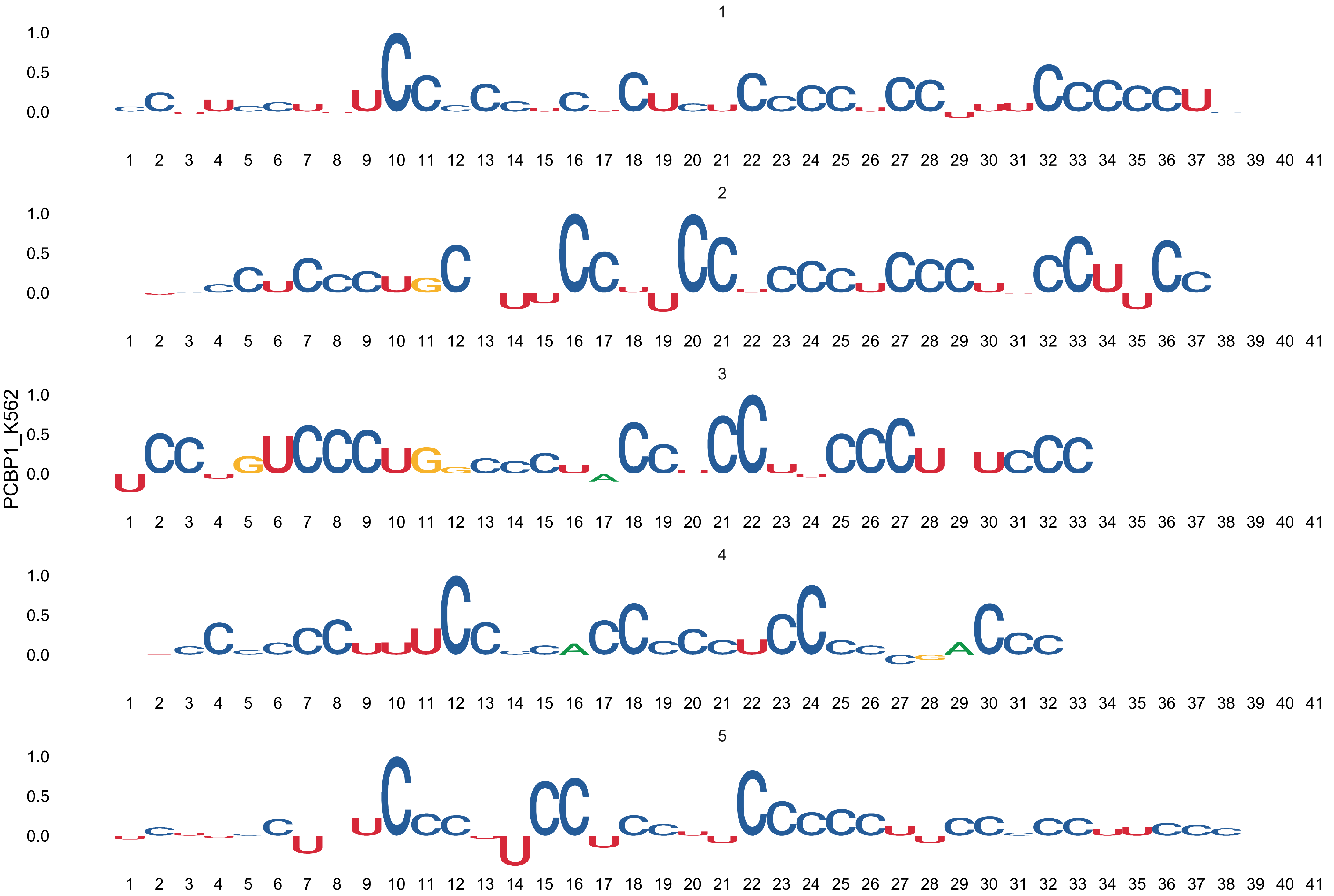
PCBP1_K562
1
2
3
4
5
1.0
0.5
0.0
1 2 3 4 5 6 7 8 9 10 11 12 13 14 15 16 17 18 19 20 21 22 23 24 25 26 27 28 29 30 31 32 33 34 35 36 37 38 39 40 41

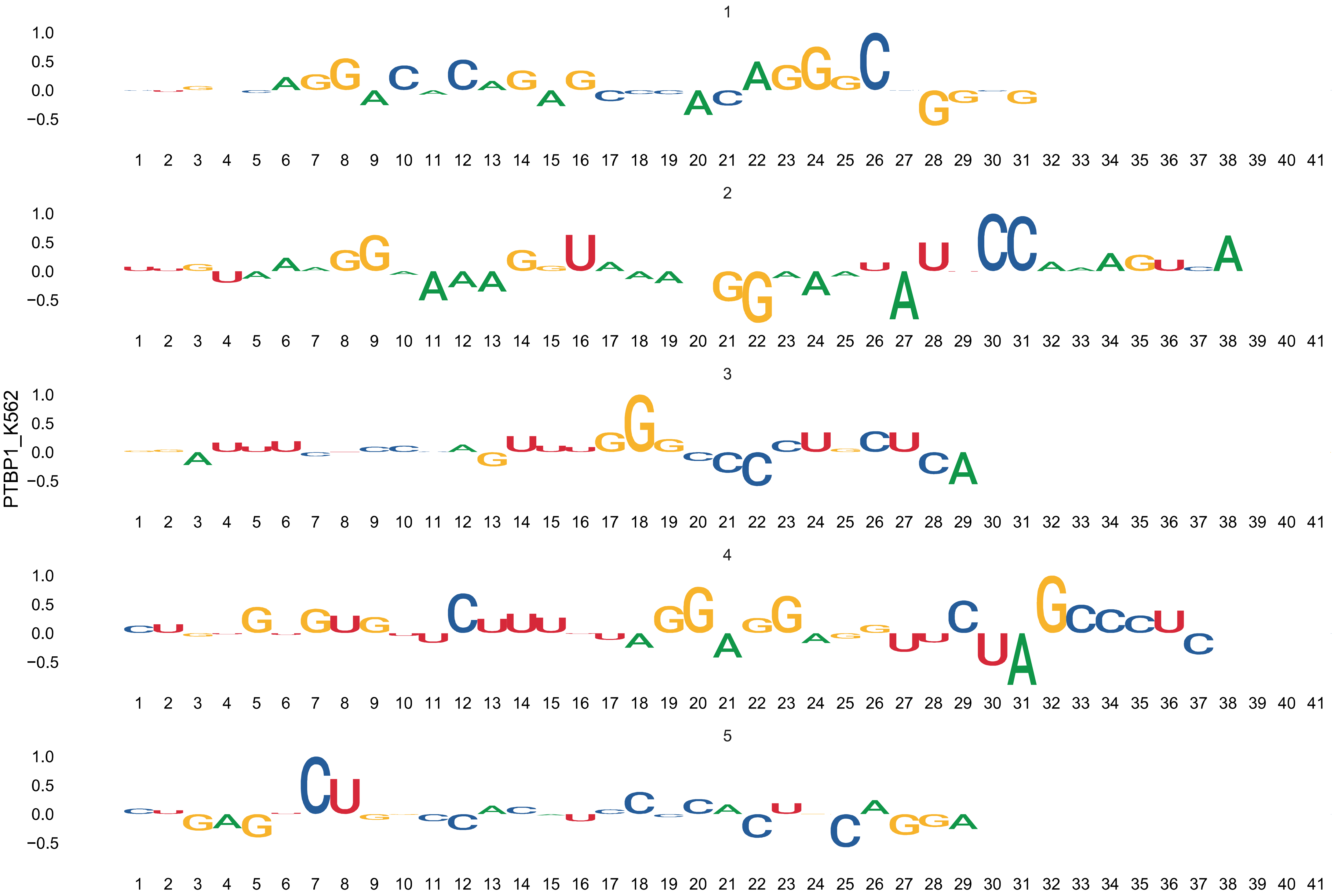
PTBP1_K562
1
2
3
4
5
1.0
0.5
0.0
−0.5
1 2 3 4 5 6 7 8 9 10 11 12 13 14 15 16 17 18 19 20 21 22 23 24 25 26 27 28 29 30 31 32 33 34 35 36 37 38 39 40 41

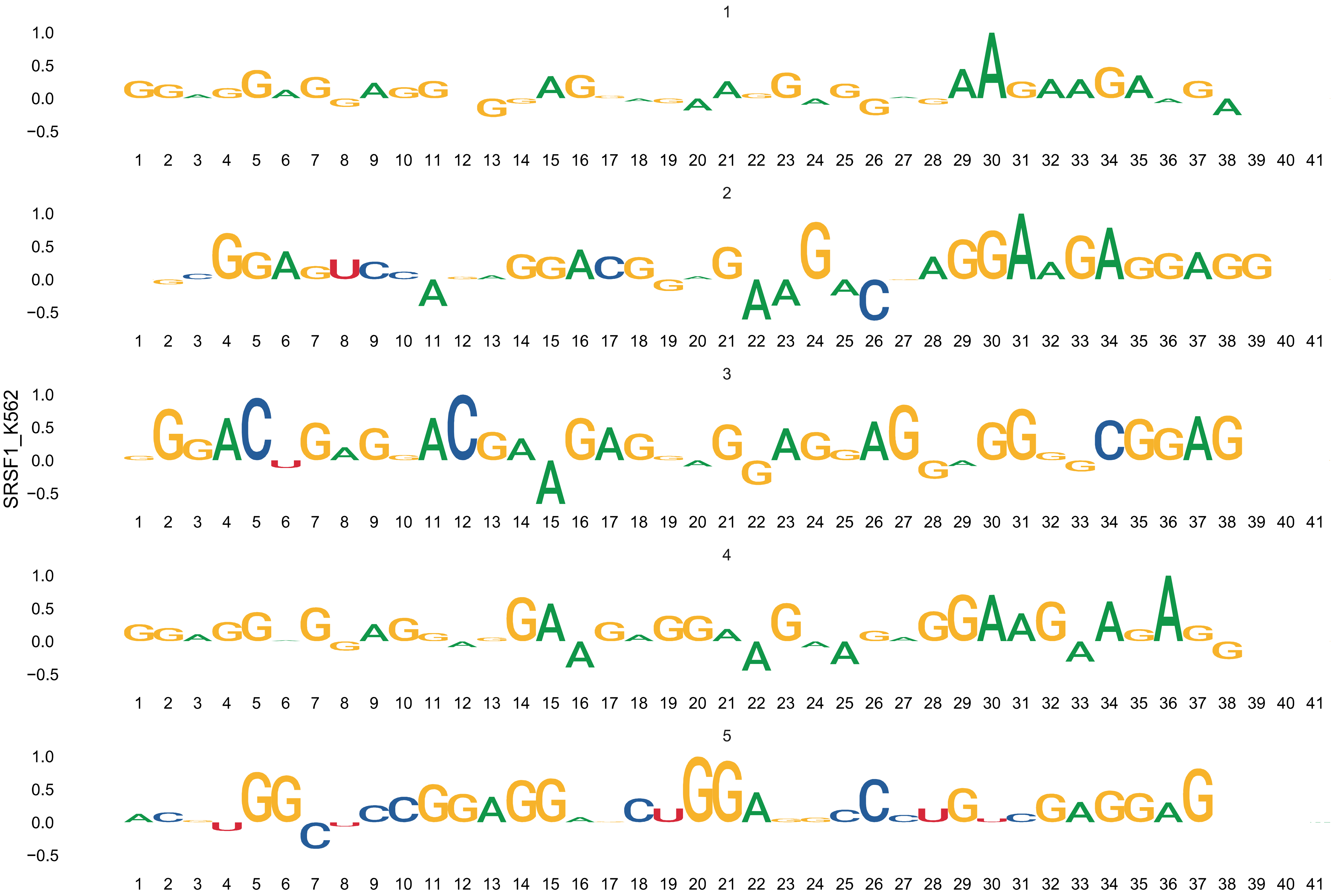
SRSF1_K562
1
2
3
4
5

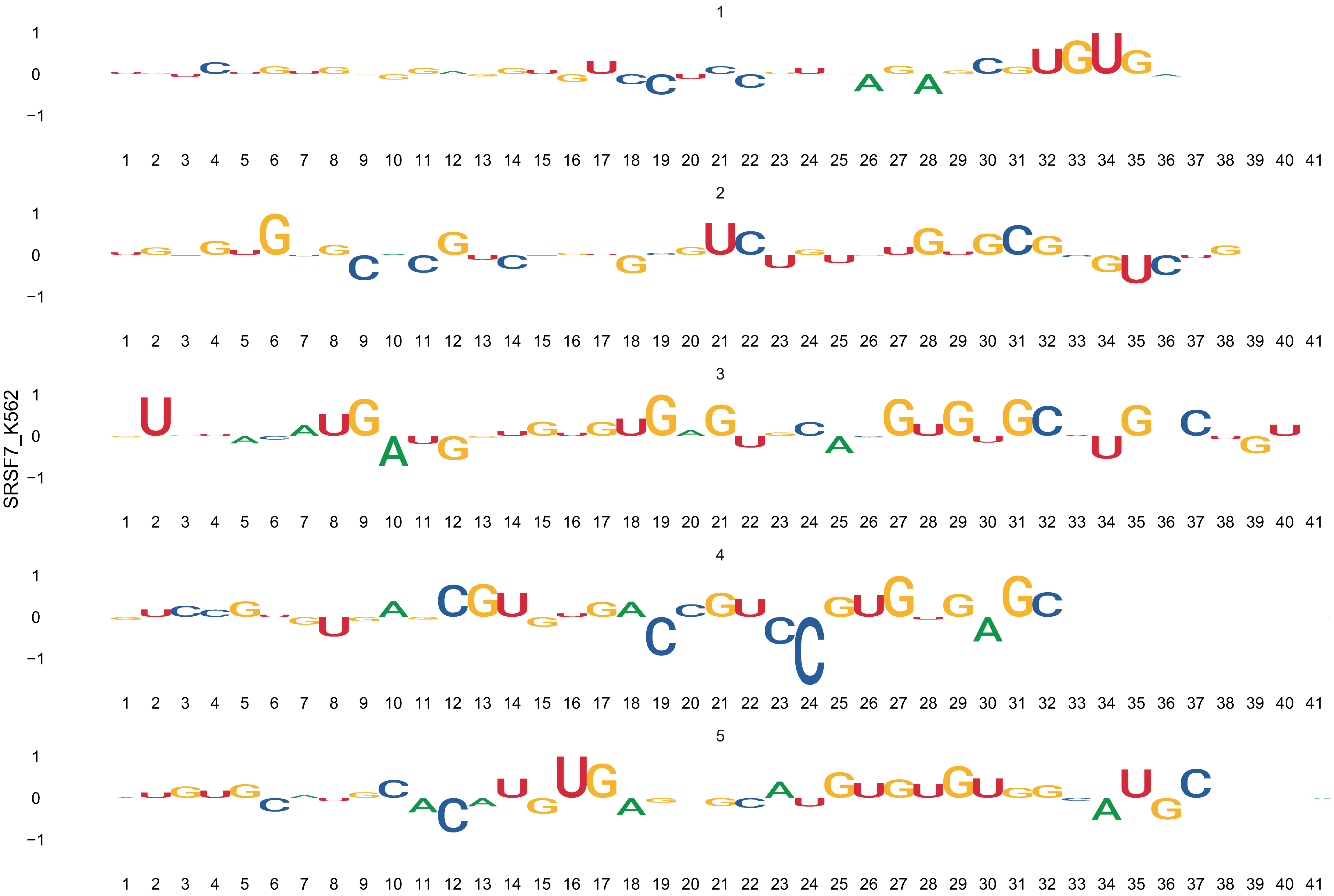
SRSF7_K562
1
2
3
4
5

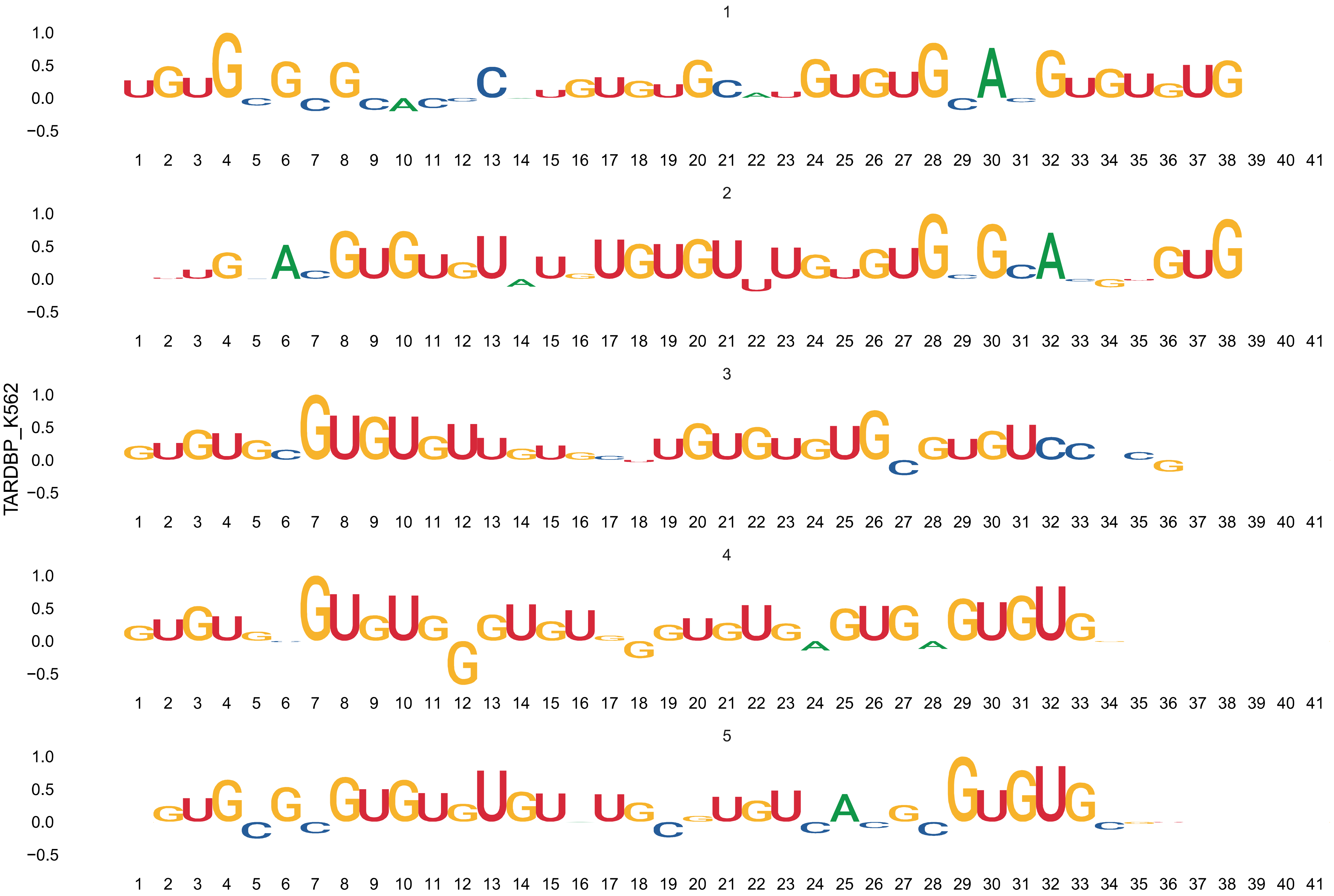
TARDBP_K562
1
2
3
4
5
1.0
0.5
0.0
−0.5
1 2 3 4 5 6 7 8 9 10 11 12 13 14 15 16 17 18 19 20 21 22 23 24 25 26 27 28 29 30 31 32 33 34 35 36 37 38 39 40 41

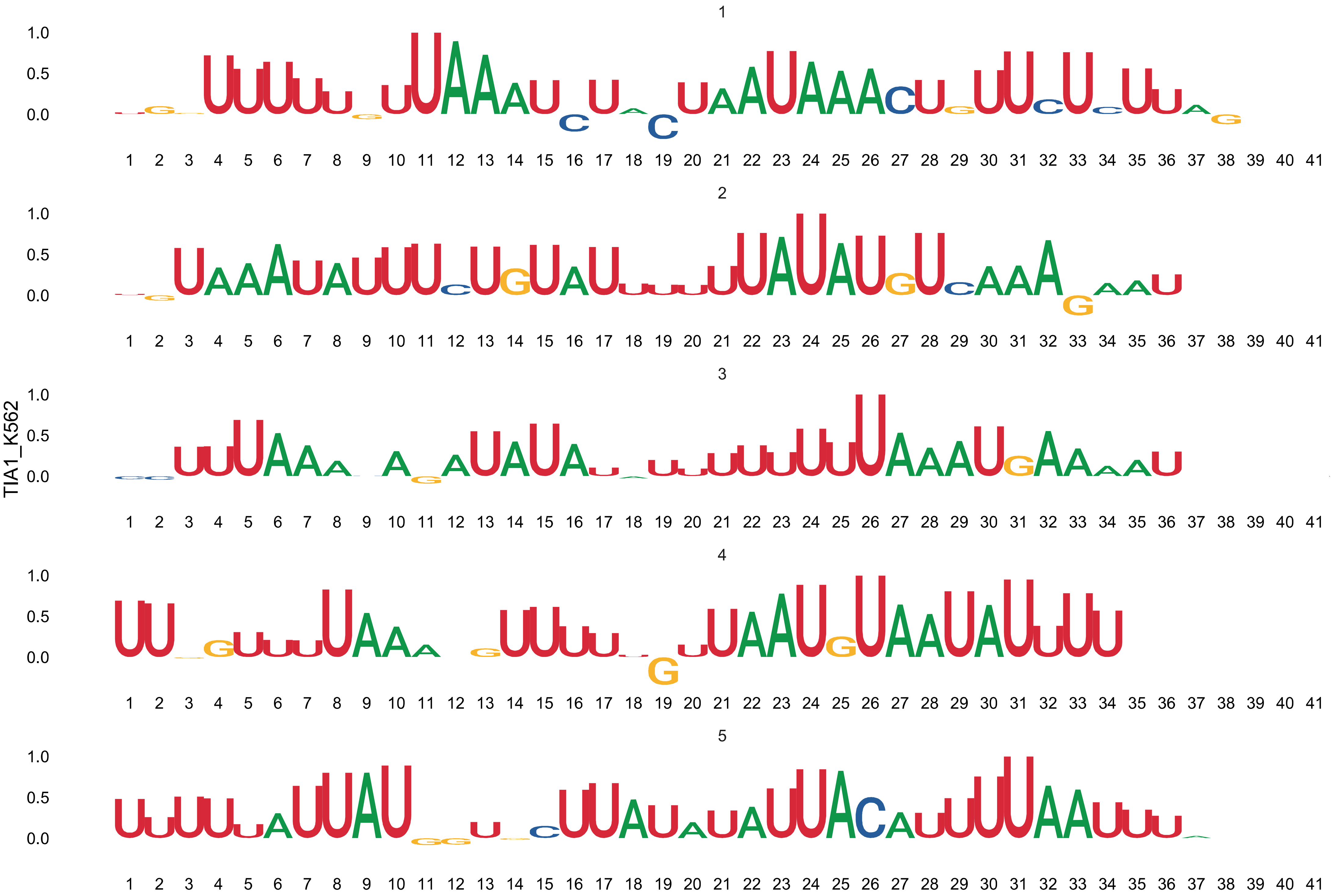
TIA1_K562
1
2
3
4
5
1.0
0.5
0.0
1 2 3 4 5 6 7 8 9 10 11 12 13 14 15 16 17 18 19 20 21 22 23 24 25 26 27 28 29 30 31 32 33 34 35 36 37 38 39 40 41

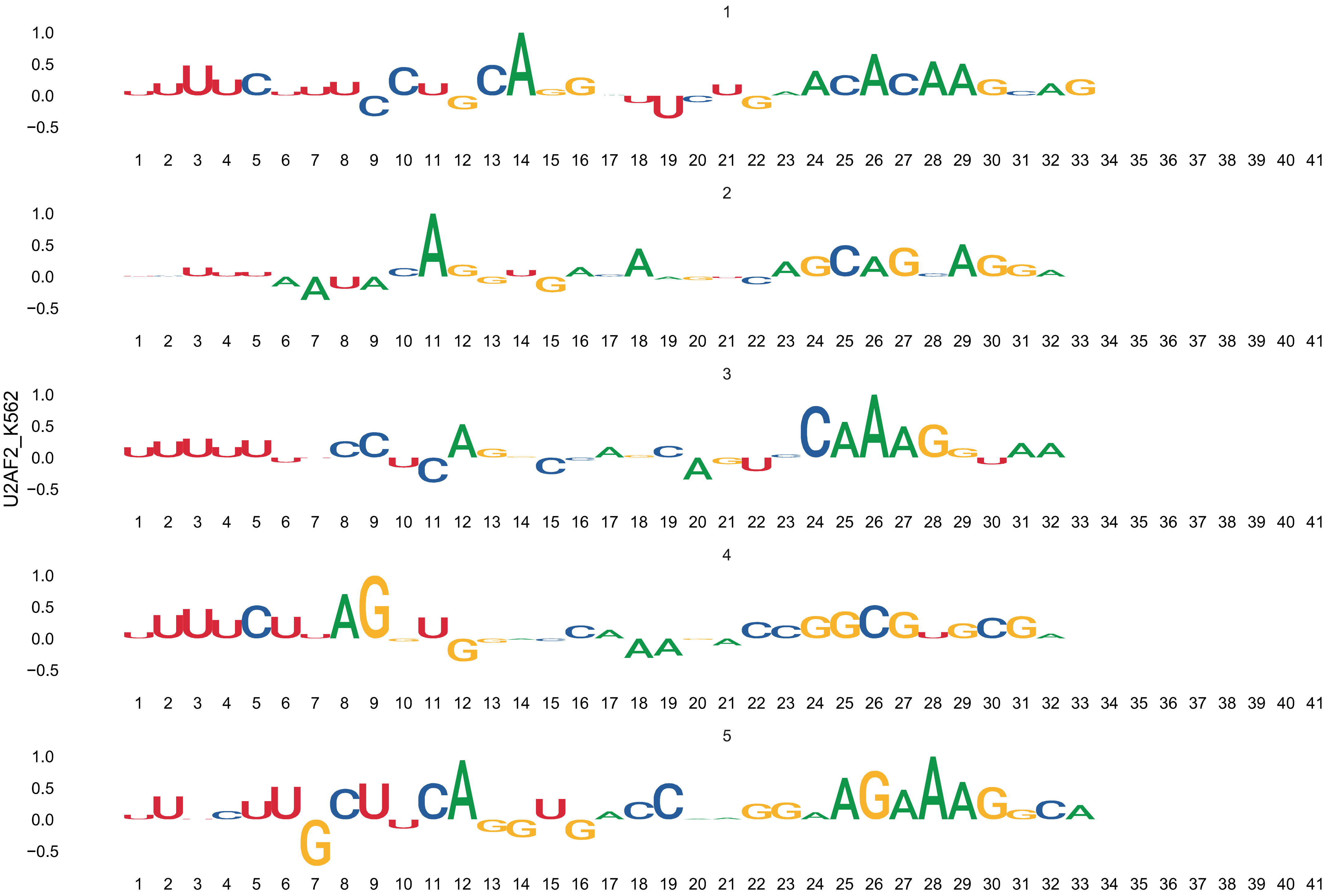
U2AF2_K562
1
2
3
4
5
1.0
0.5
0.0
−0.5
1 2 3 4 5 6 7 8 9 10 11 12 13 14 15 16 17 18 19 20 21 22 23 24 25 26 27 28 29 30 31 32 33 34 35 36 37 38 39 40 41

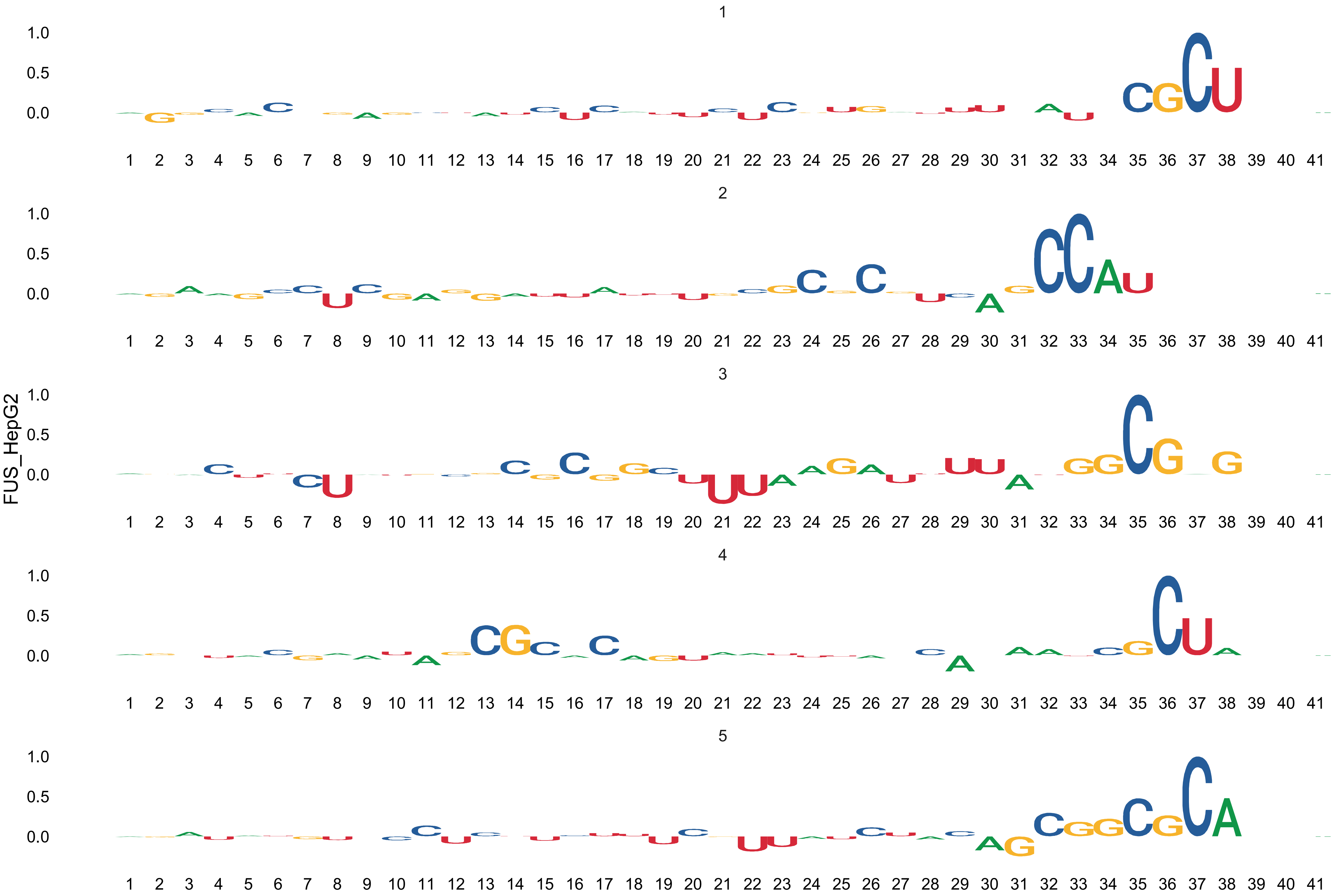
FUS_HepG2
1
2
3
4
5
1.0
0.5
0.0
1 2 3 4 5 6 7 8 9 10 11 12 13 14 15 16 17 18 19 20 21 22 23 24 25 26 27 28 29 30 31 32 33 34 35 36 37 38 39 40 41

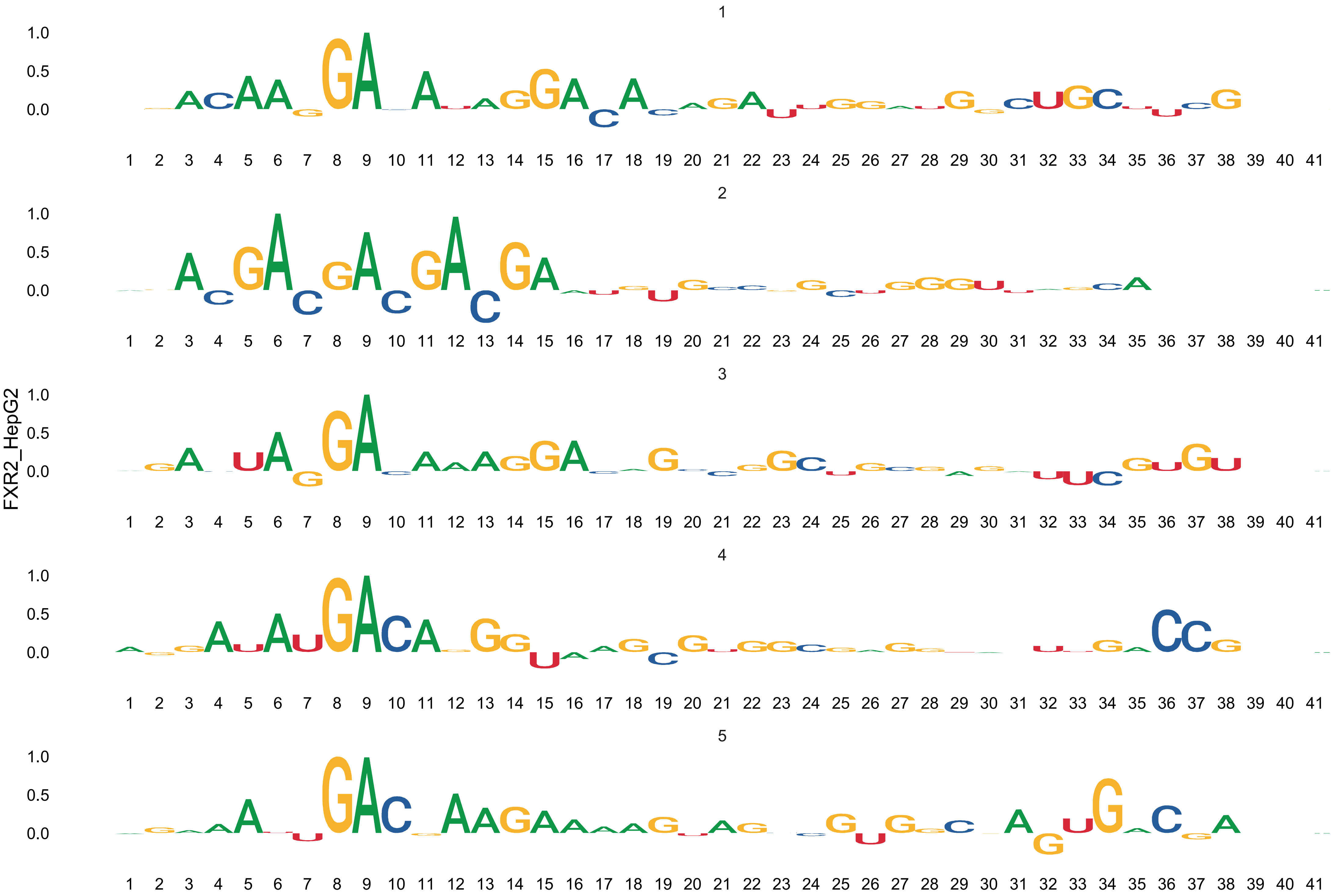

FXR2_HepG2
1
2
3
4
5
1.0
0.5
0.0
1 2 3 4 5 6 7 8 9 10 11 12 13 14 15 16 17 18 19 20 21 22 23 24 25 26 27 28 29 30 31 32 33 34 35 36 37 38 39 40 41

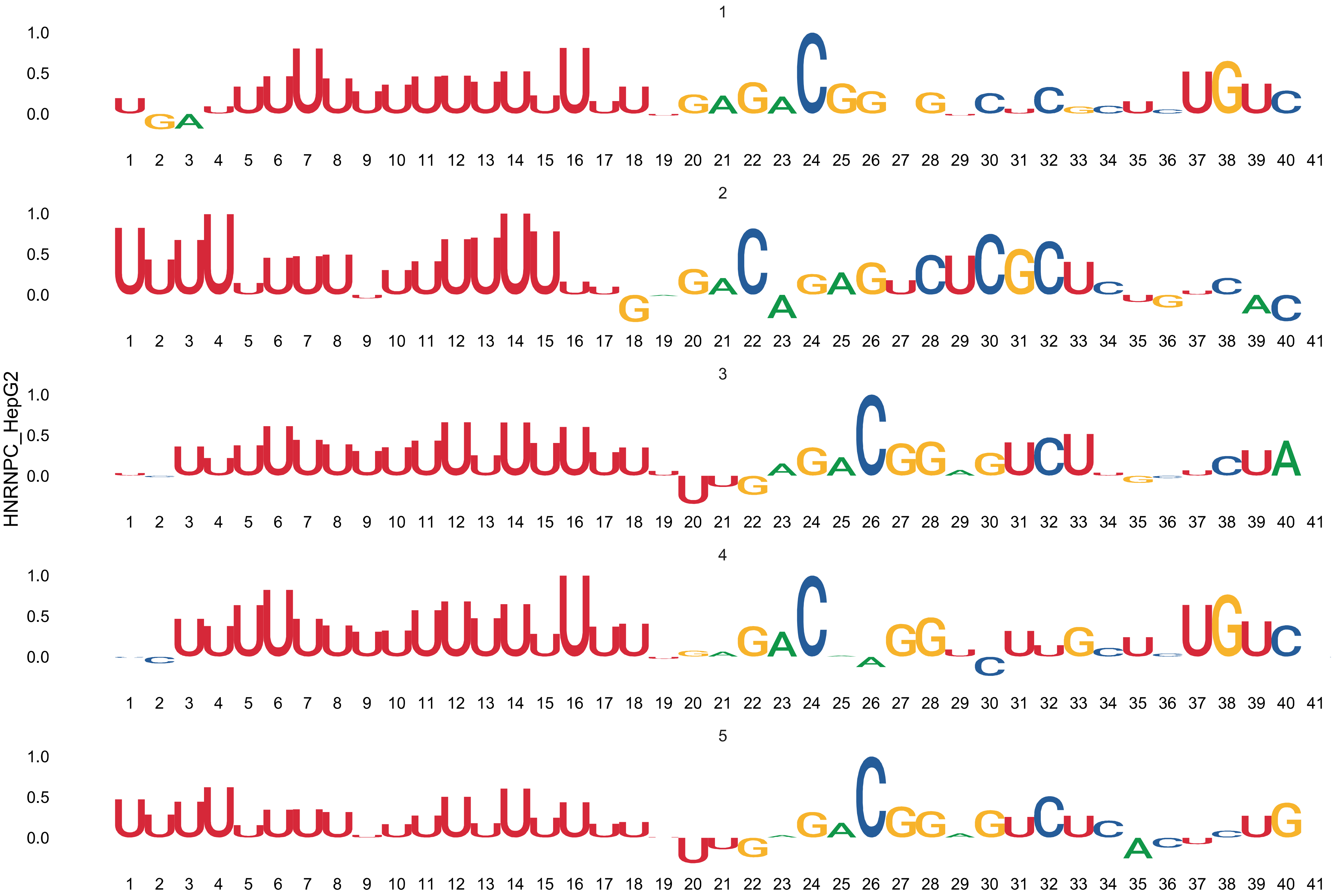
HNRNPC_HepG2
1
2
3
4
5
1.0
0.5
0.0
1 2 3 4 5 6 7 8 9 10 11 12 13 14 15 16 17 18 19 20 21 22 23 24 25 26 27 28 29 30 31 32 33 34 35 36 37 38 39 40 41

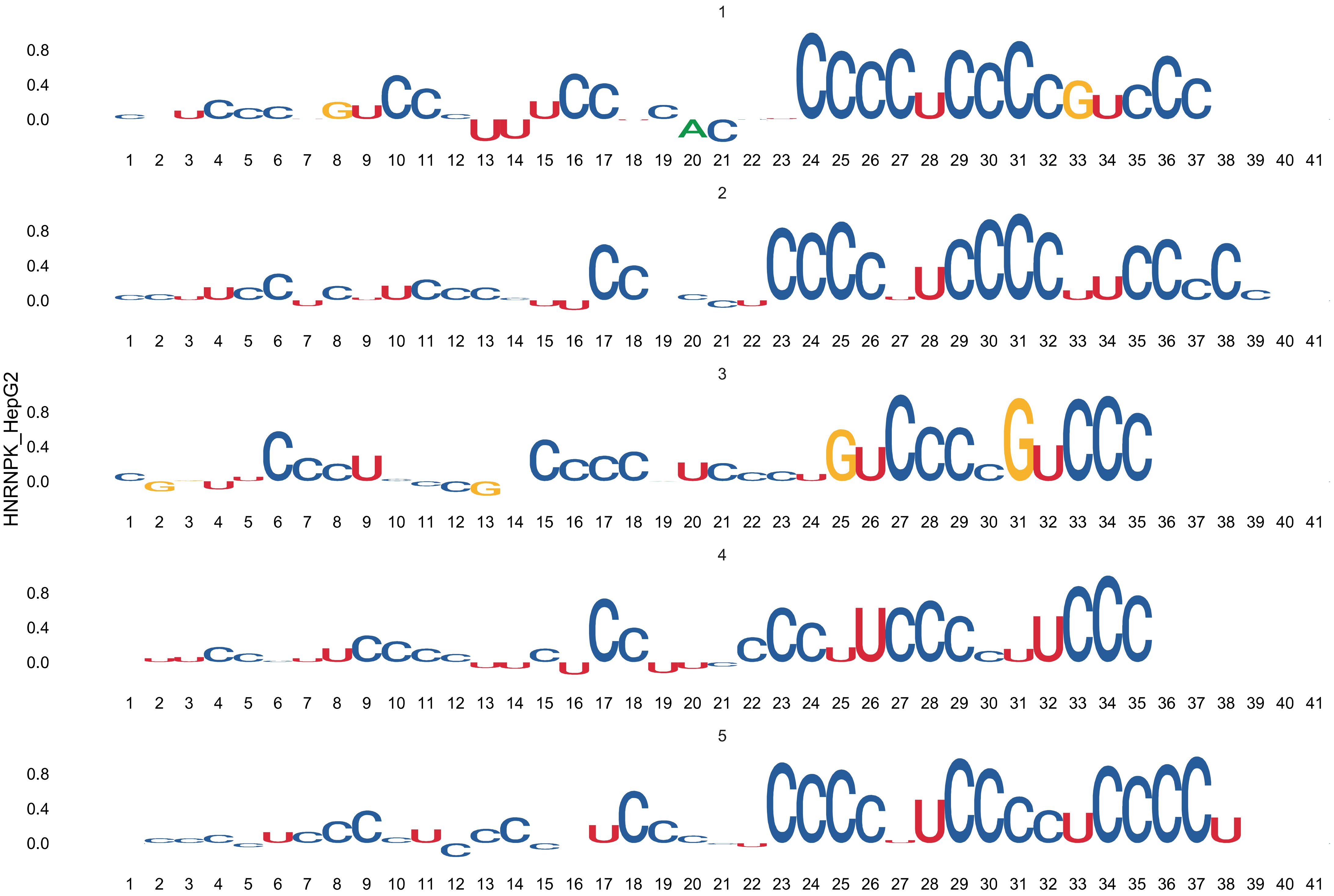
HNRNPK_HepG2
1
2
3
4
5
0.8
0.4
0.0

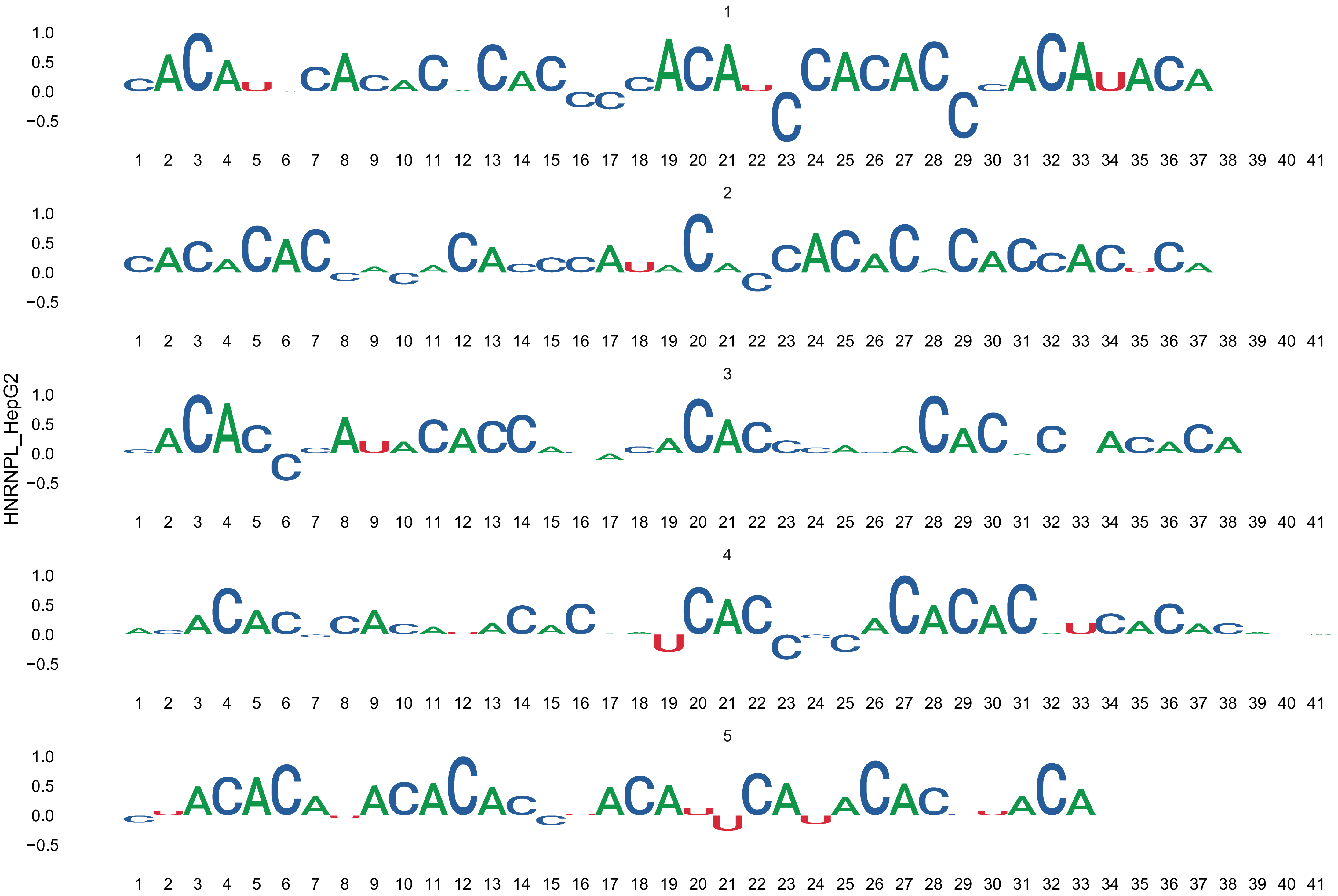
HNRNPL_HepG2
1
2
3
4
5
1.0
0.5
0.0
−0.5
1 2 3 4 5 6 7 8 9 10 11 12 13 14 15 16 17 18 19 20 21 22 23 24 25 26 27 28 29 30 31 32 33 34 35 36 37 38 39 40 41

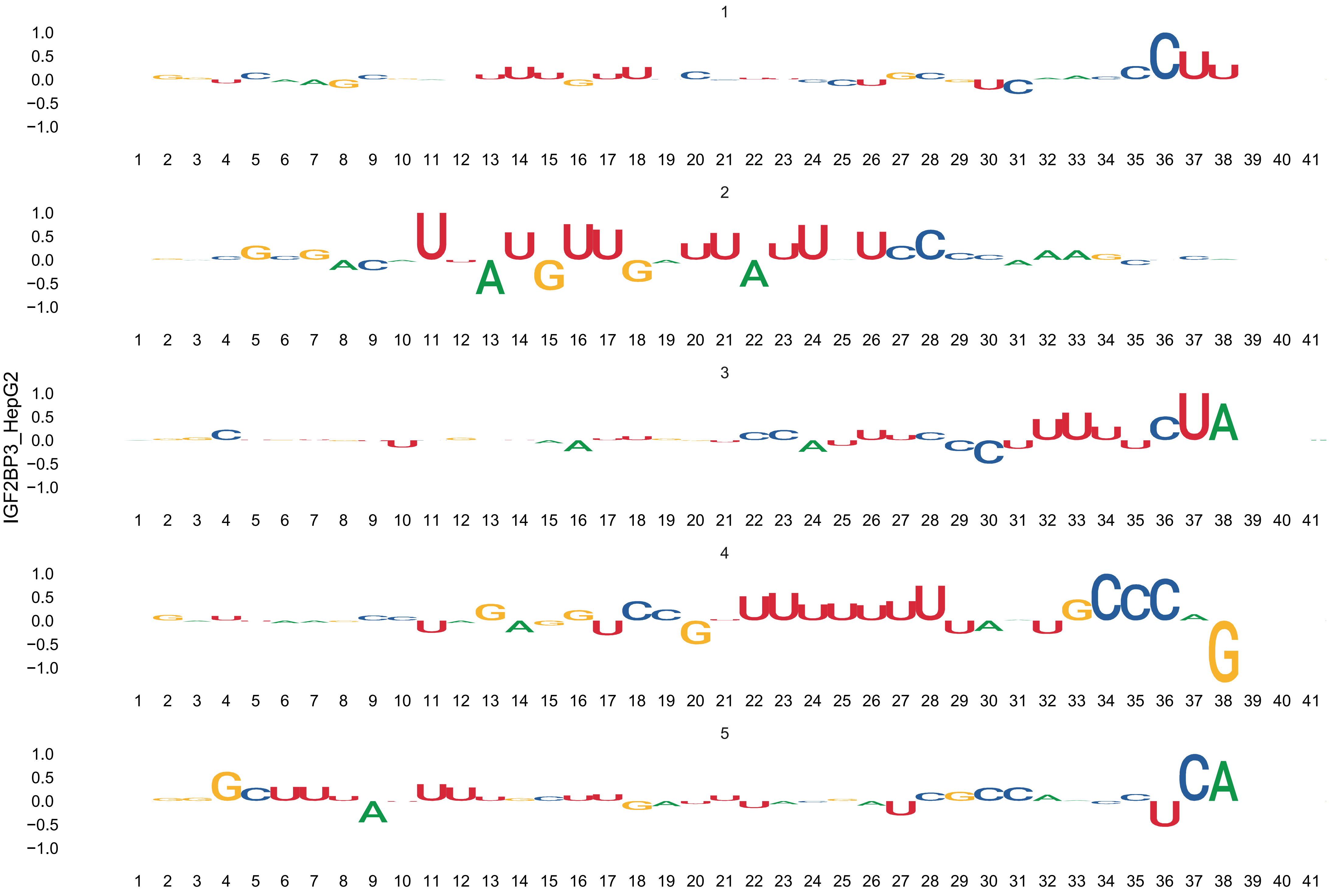
IGF2BP3_HepG2
1
2
3
4
5
1.0
0.5
0.0
−0.5
−1.0
1 2 3 4 5 6 7 8 9 10 11 12 13 14 15 16 17 18 19 20 21 22 23 24 25 26 27 28 29 30 31 32 33 34 35 36 37 38 39 40 41

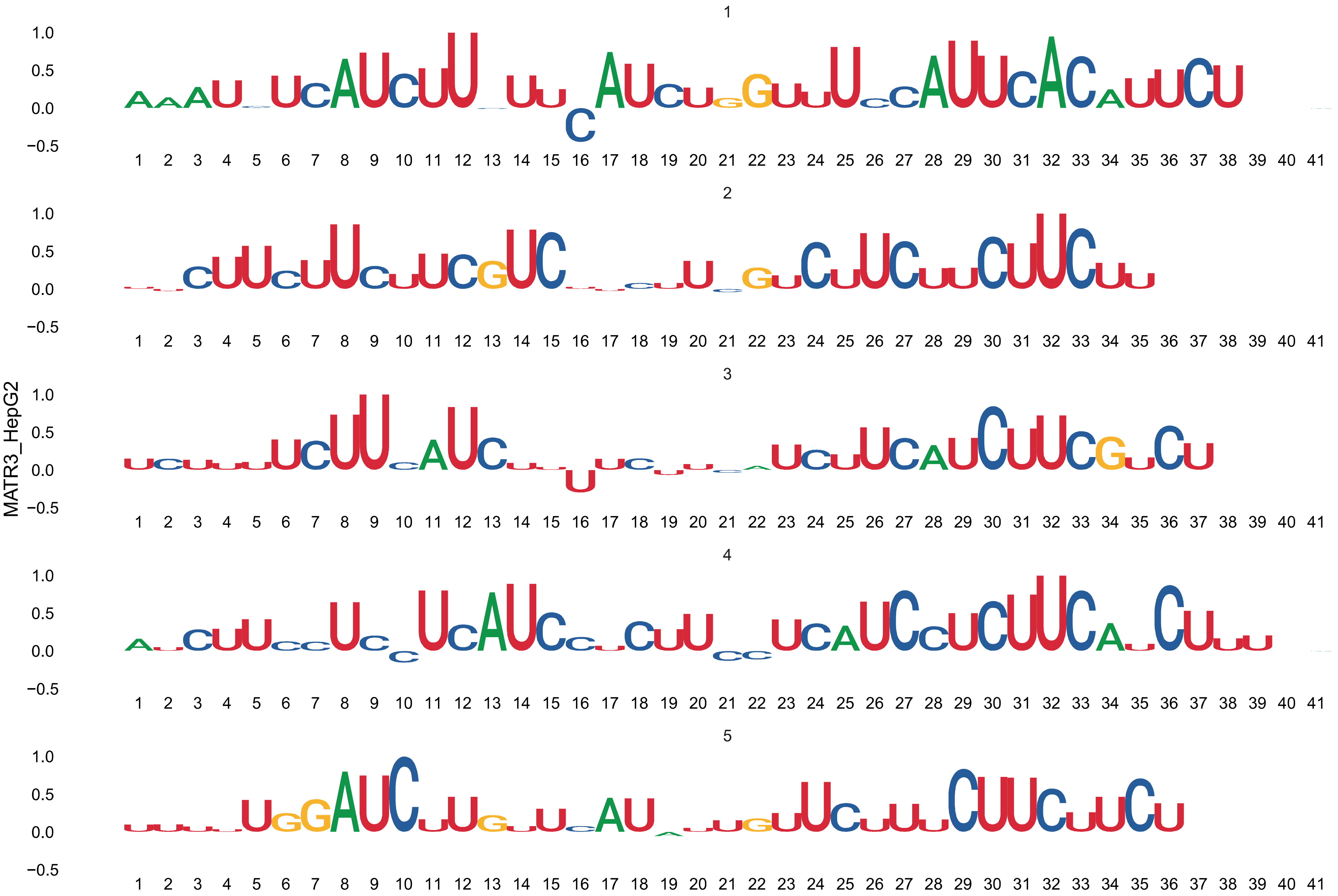
MATR3_HepG2
1
2
3
4
5

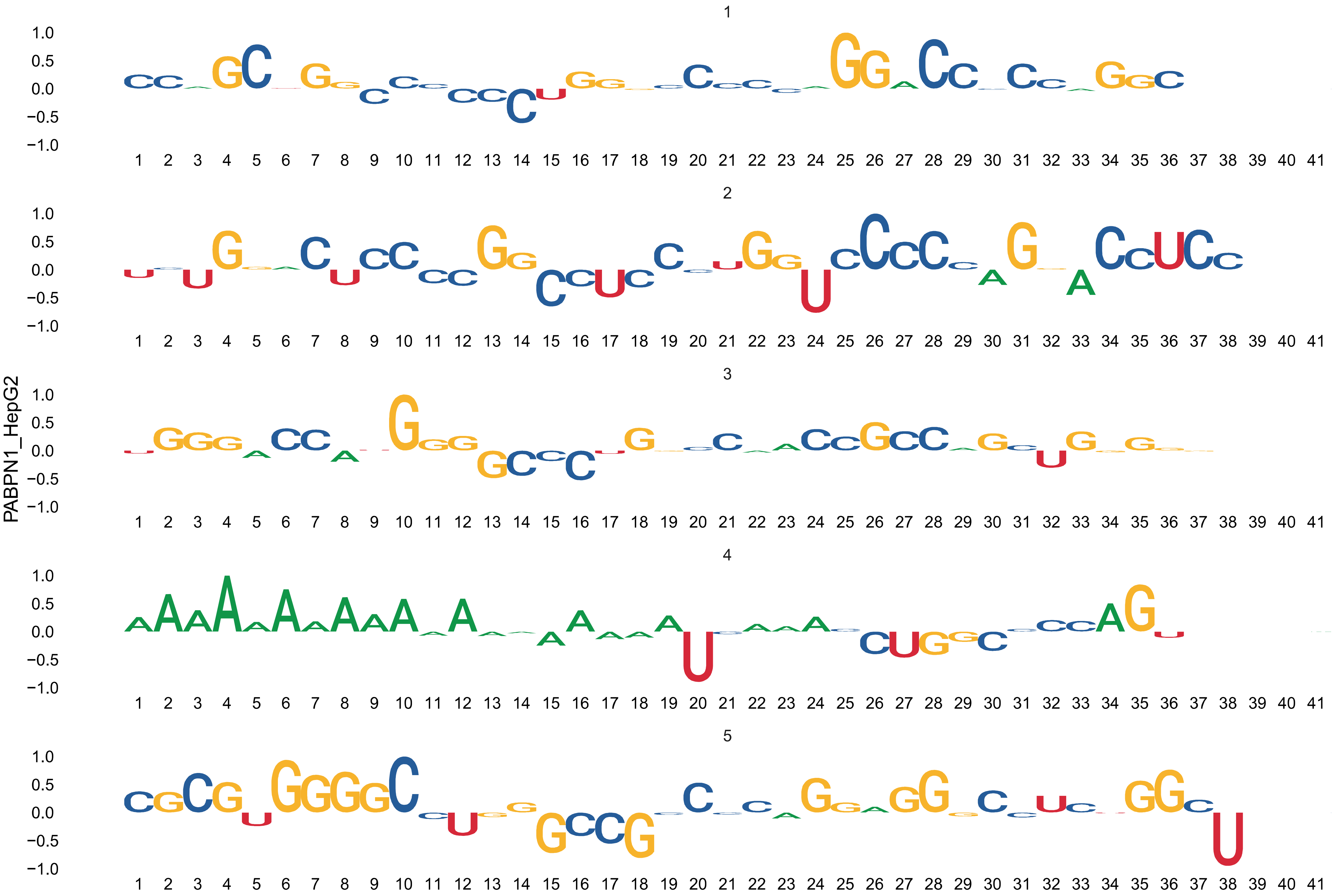
PABPN1_HepG2
1
2
3
4
5
1.0
0.5
0.0
−0.5
−1.0
1 2 3 4 5 6 7 8 9 10 11 12 13 14 15 16 17 18 19 20 21 22 23 24 25 26 27 28 29 30 31 32 33 34 35 36 37 38 39 40 41

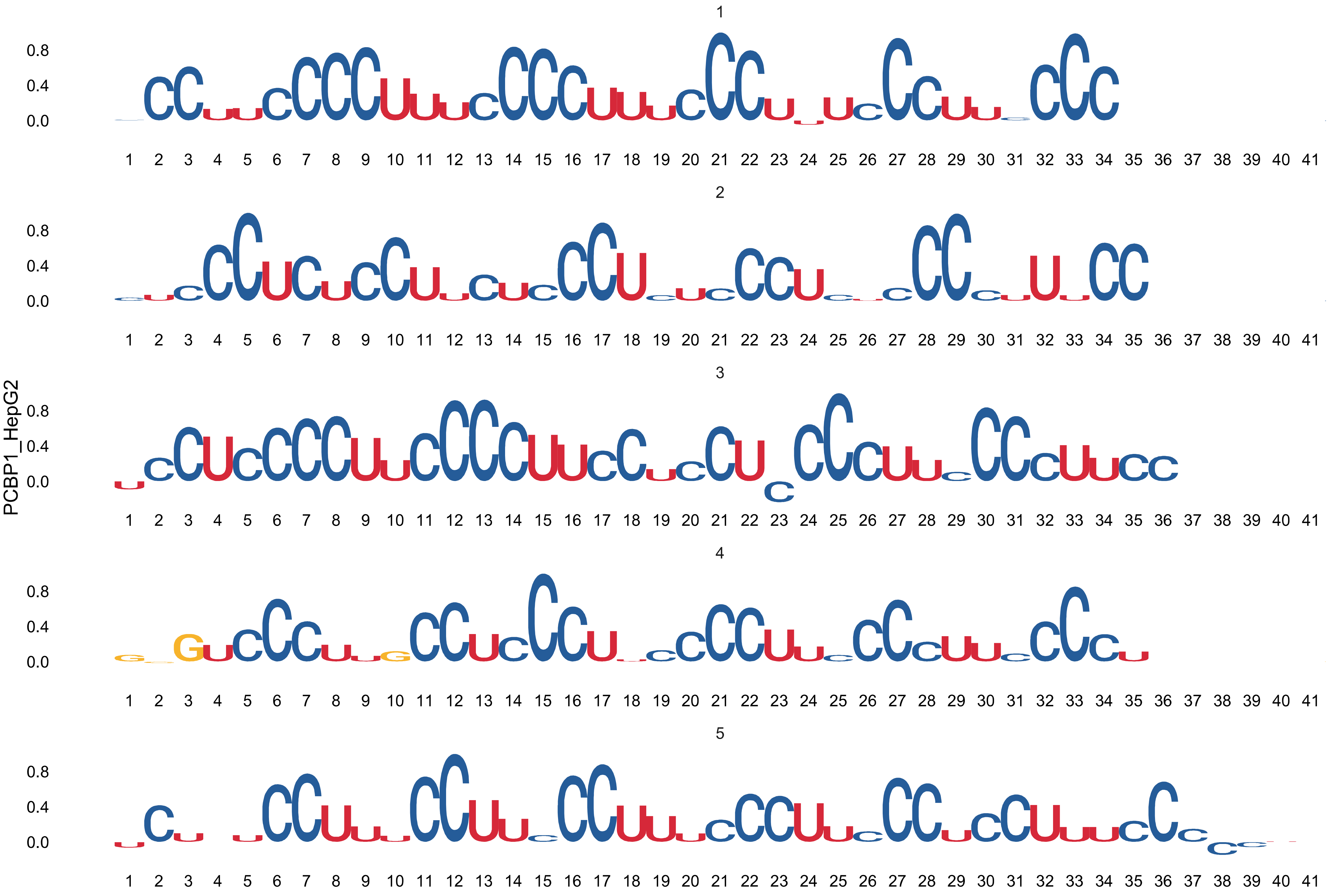
PCBP1_HepG2
1
2
3
4
5
0.8
0.4
0.0
1 2 3 4 5 6 7 8 9 10 11 12 13 14 15 16 17 18 19 20 21 22 23 24 25 26 27 28 29 30 31 32 33 34 35 36 37 38 39 40 41

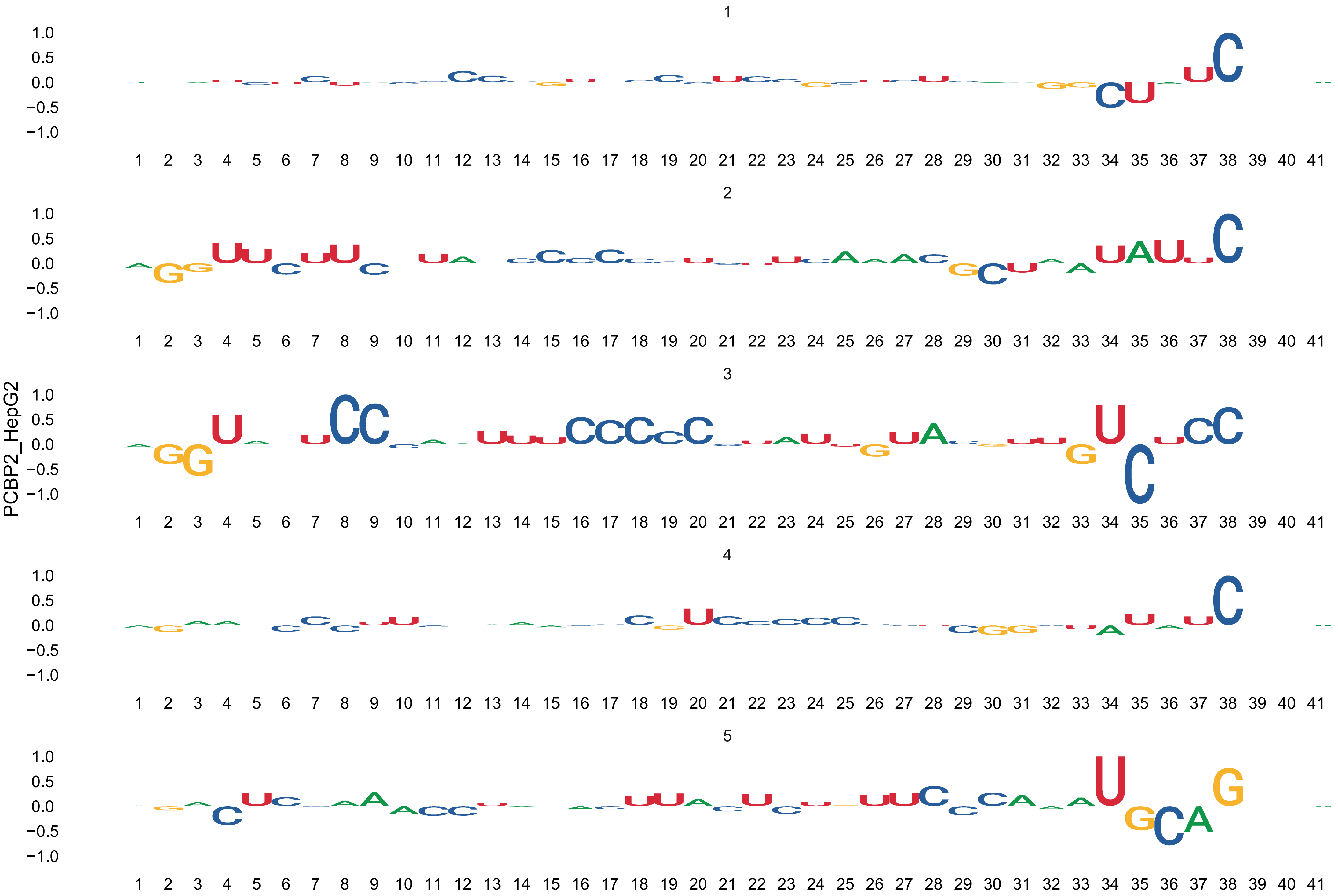
PCBP2_HepG2
1
2
3
4
5
1.0
0.5
0.0
−0.5
−1.0
1 2 3 4 5 6 7 8 9 10 11 12 13 14 15 16 17 18 19 20 21 22 23 24 25 26 27 28 29 30 31 32 33 34 35 36 37 38 39 40 41

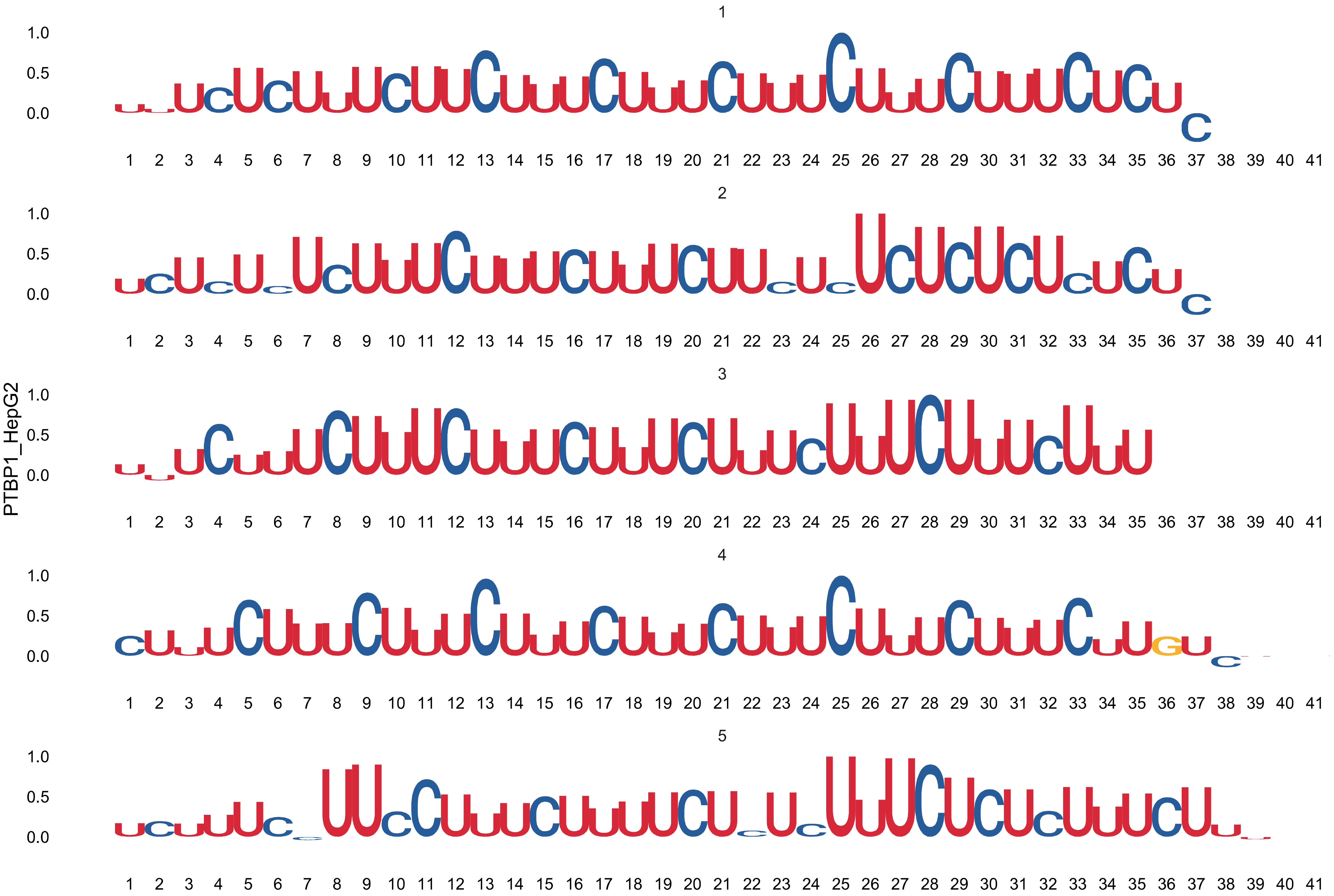
PTBP1_HepG2
1
2
3
4
5
1.0
0.5
0.0
1 2 3 4 5 6 7 8 9 10 11 12 13 14 15 16 17 18 19 20 21 22 23 24 25 26 27 28 29 30 31 32 33 34 35 36 37 38 39 40 41

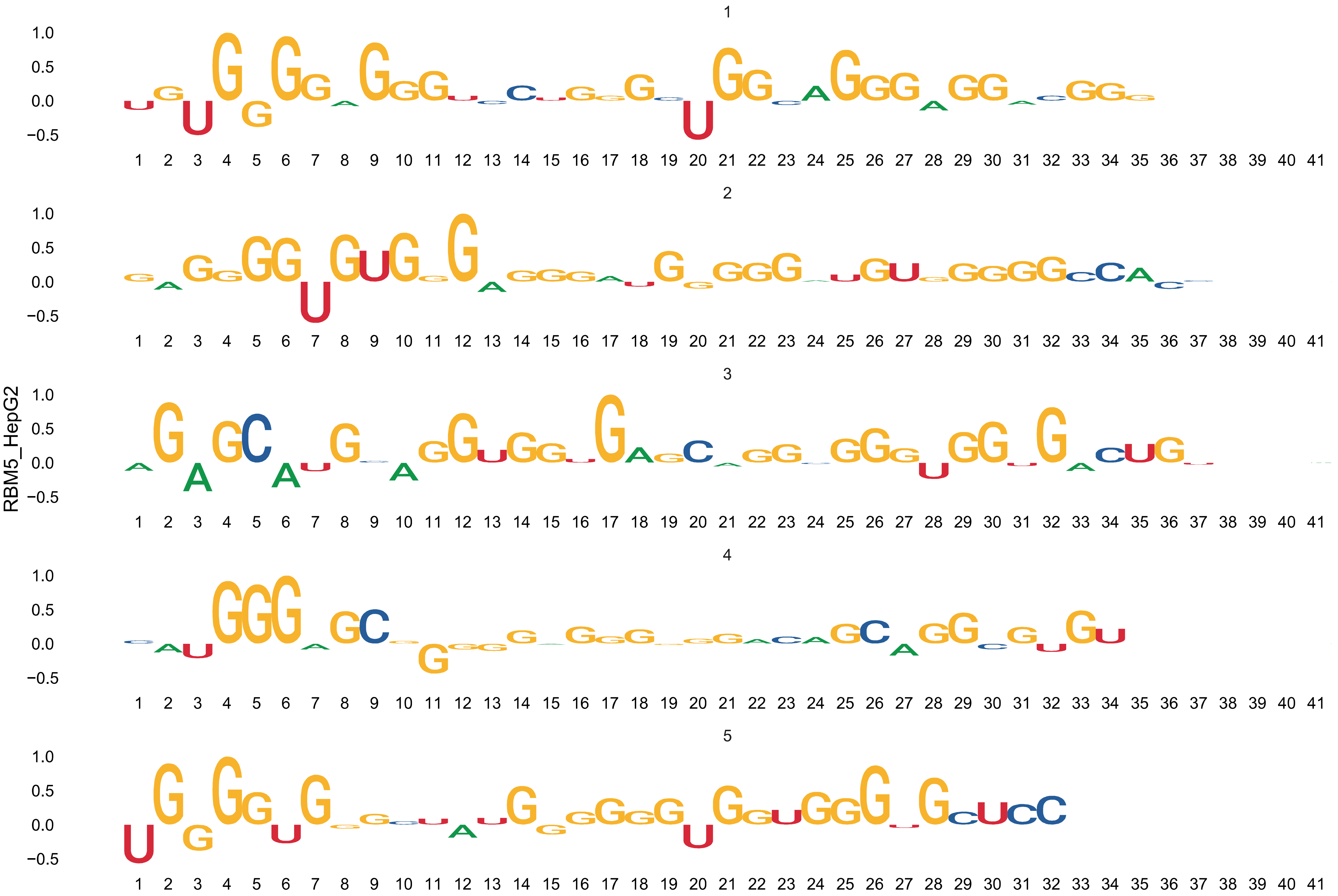
RBM5_HepG2
1
2
3
4
5
1.0
0.5
0.0
−0.5
1 2 3 4 5 6 7 8 9 10 11 12 13 14 15 16 17 18 19 20 21 22 23 24 25 26 27 28 29 30 31 32 33 34 35 36 37 38 39 40 41

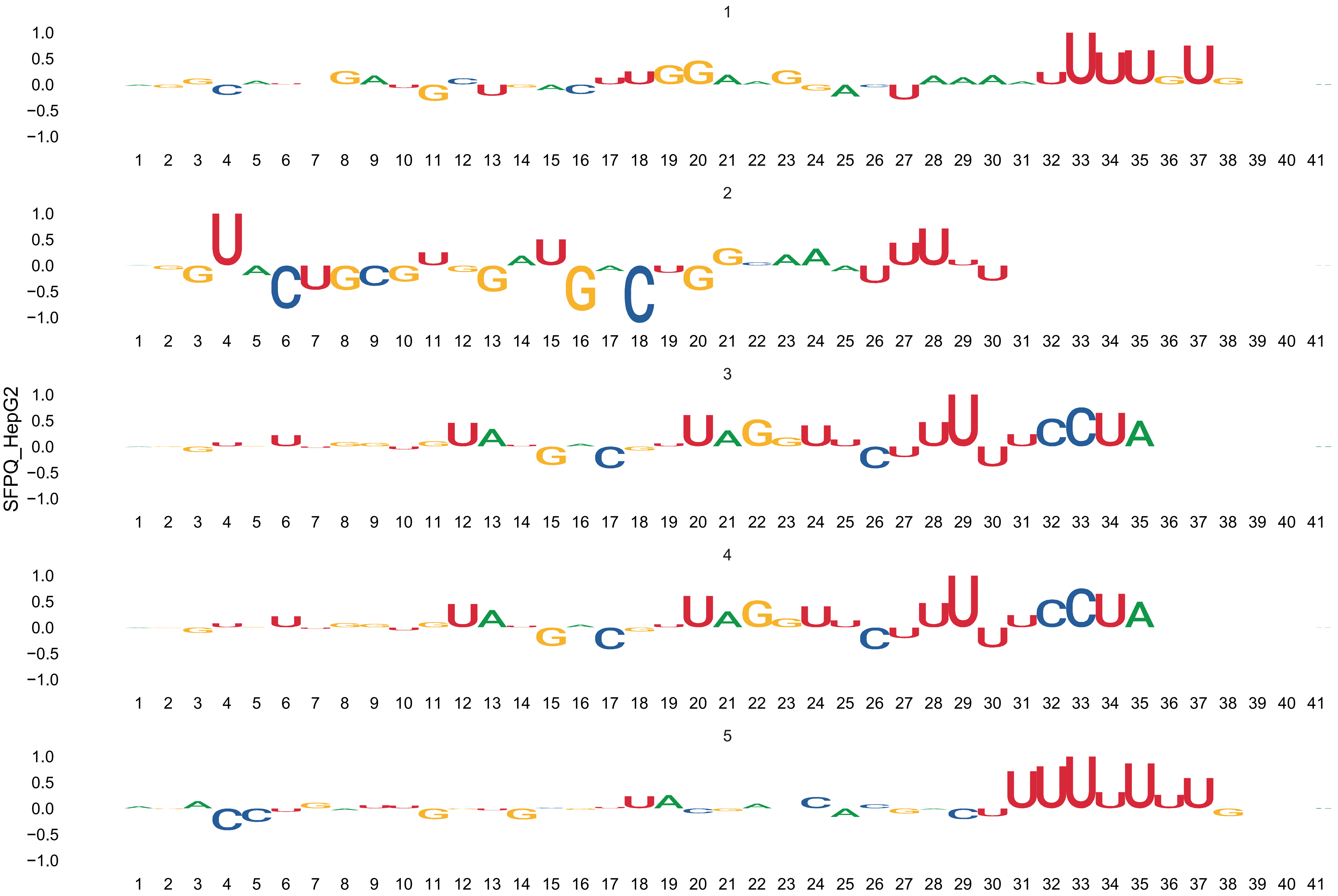

SFPQ_HepG2
1
2
3
4
5
1.0
0.5
0.0
−0.5
−1.0
1 2 3 4 5 6 7 8 9 10 11 12 13 14 15 16 17 18 19 20 21 22 23 24 25 26 27 28 29 30 31 32 33 34 35 36 37 38 39 40 41

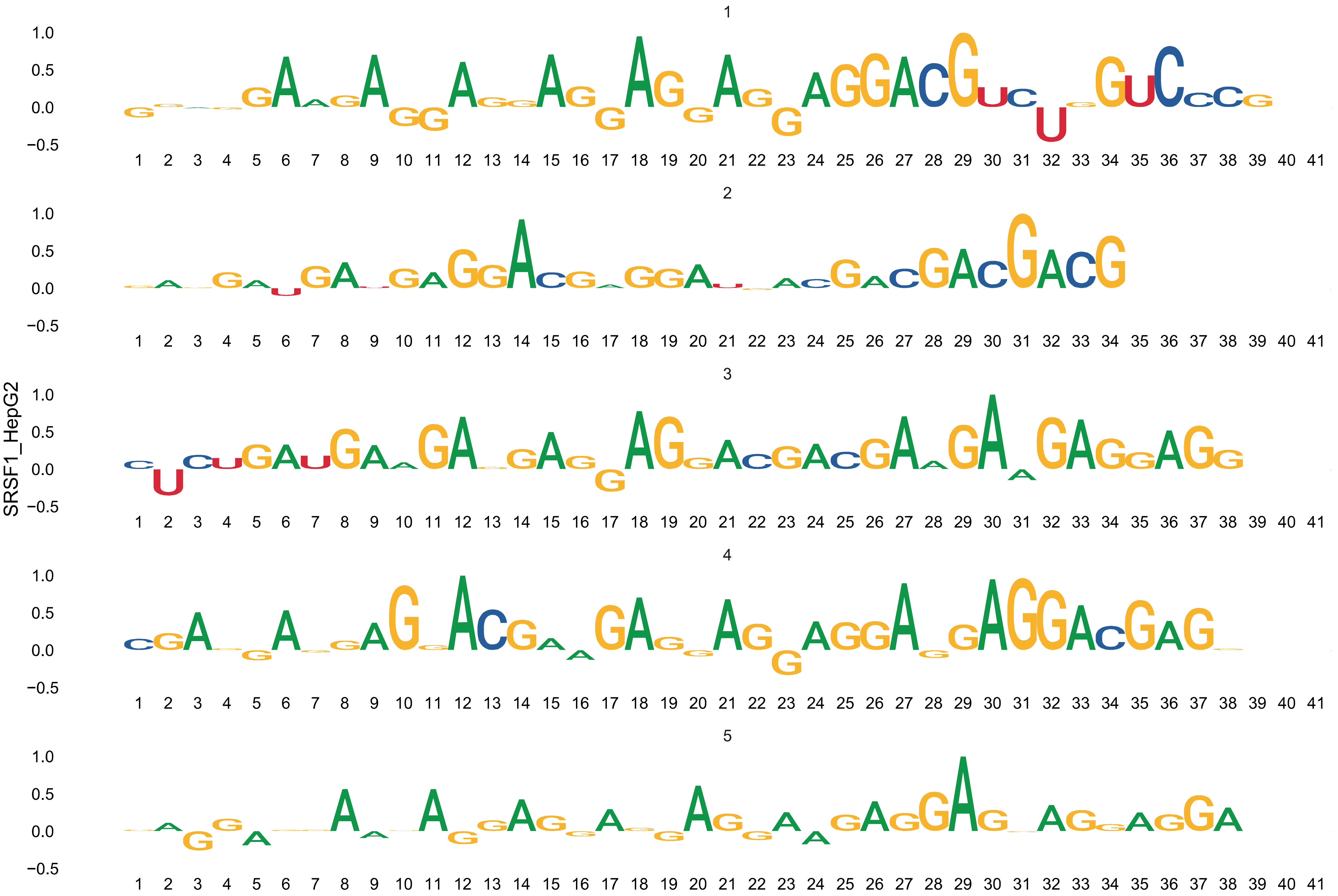
SRSF1_HepG2
1
2
3
4
5
1.0
0.5
0.0
−0.5
1 2 3 4 5 6 7 8 9 10 11 12 13 14 15 16 17 18 19 20 21 22 23 24 25 26 27 28 29 30 31 32 33 34 35 36 37 38 39 40 41

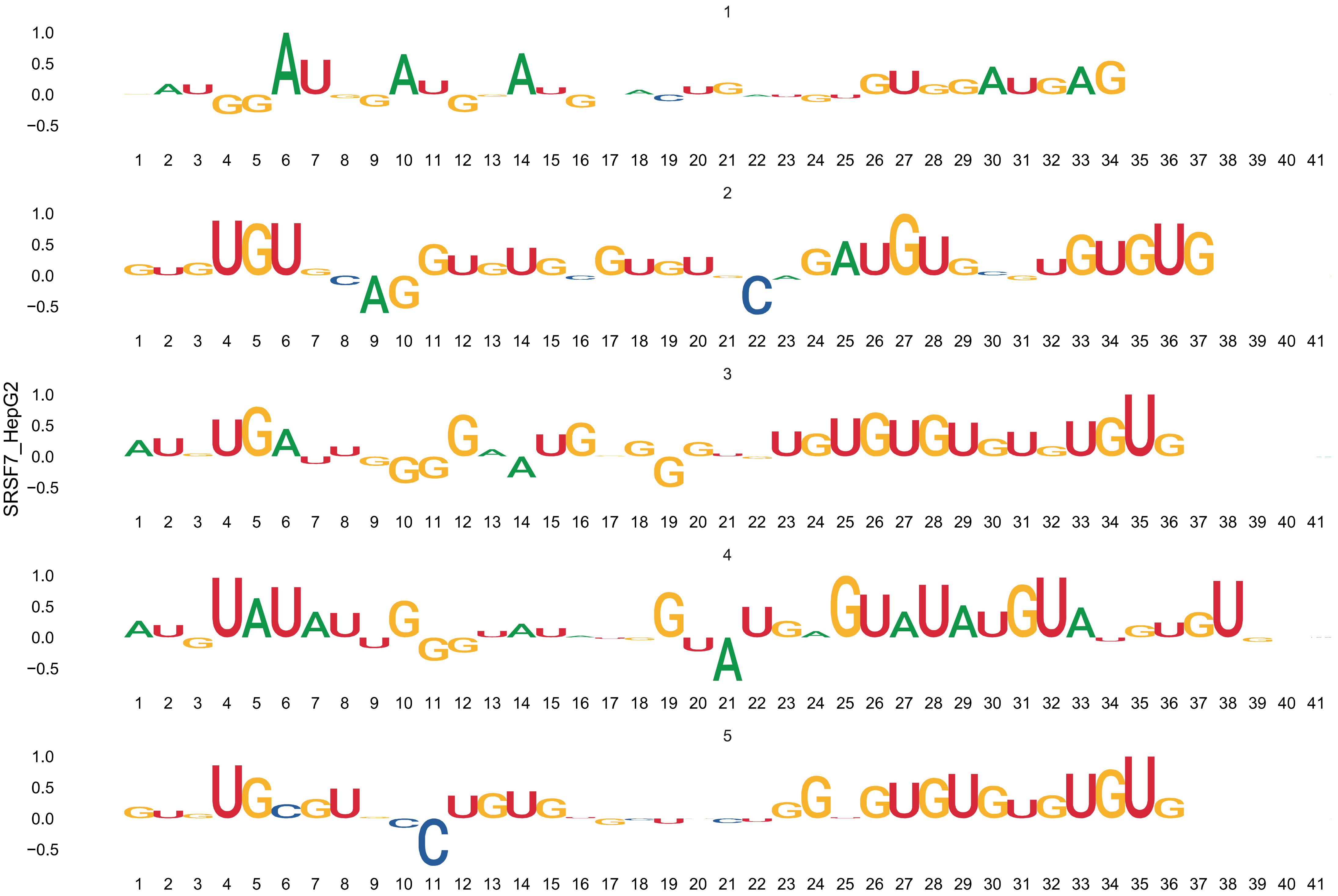
SRSF7_HepG2
1
2
3
4
5

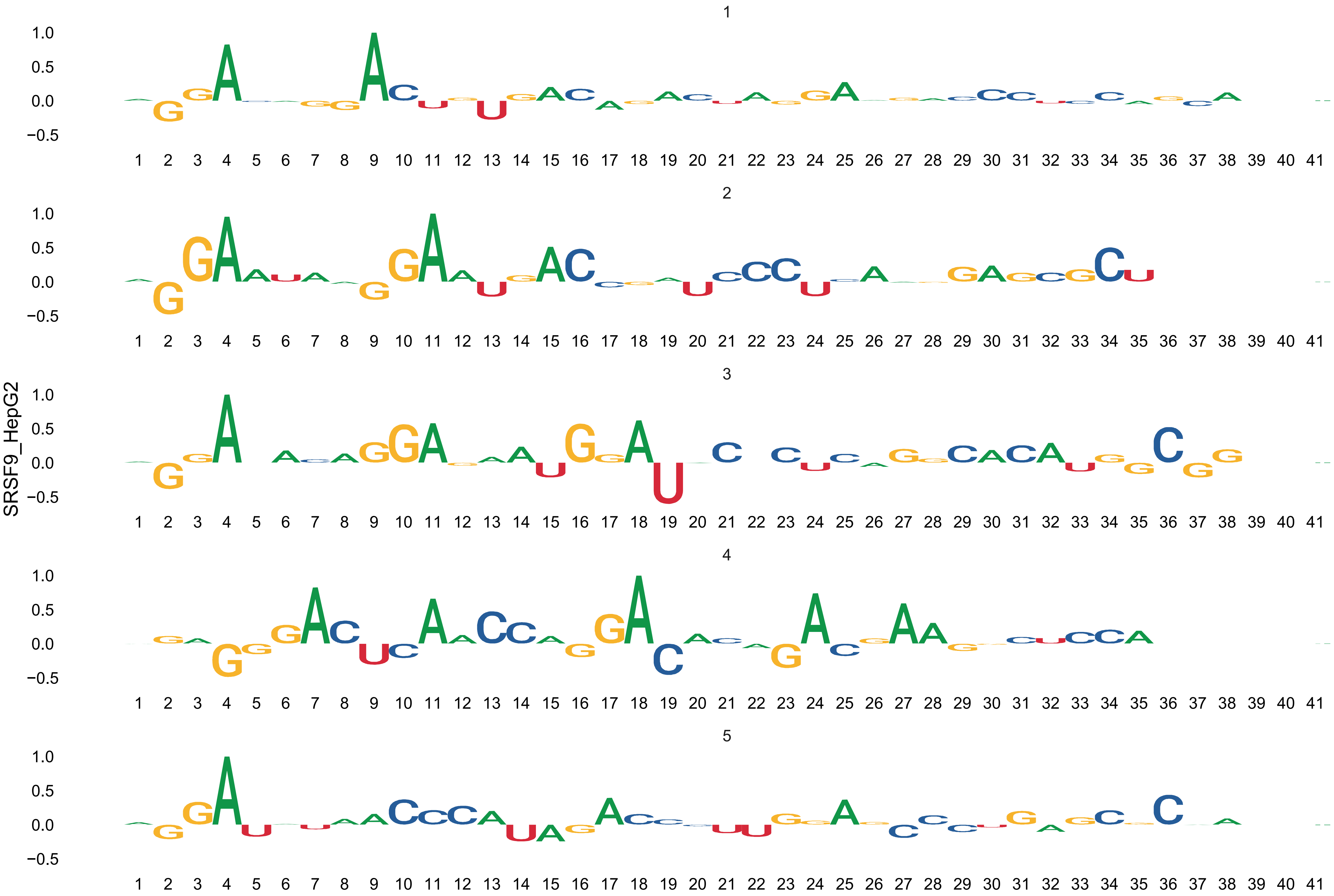
SRSF9_HepG2
1
2
3
4
5
1.0
0.5
0.0
−0.5
1 2 3 4 5 6 7 8 9 10 11 12 13 14 15 16 17 18 19 20 21 22 23 24 25 26 27 28 29 30 31 32 33 34 35 36 37 38 39 40 41

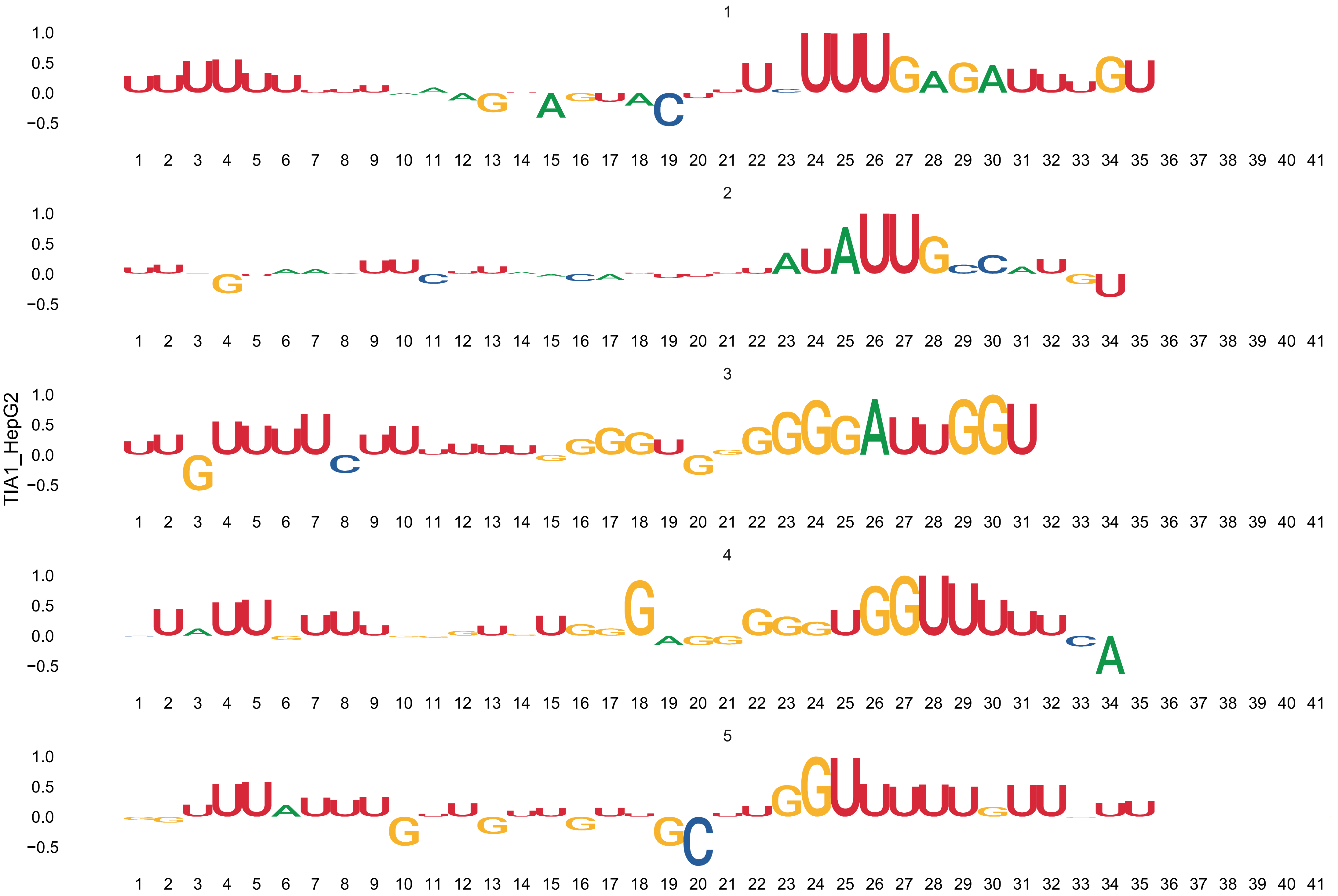
TIA1_HepG2
1
2
3
4
5
1.0
0.5
0.0
−0.5
1 2 3 4 5 6 7 8 9 10 11 12 13 14 15 16 17 18 19 20 21 22 23 24 25 26 27 28 29 30 31 32 33 34 35 36 37 38 39 40 41

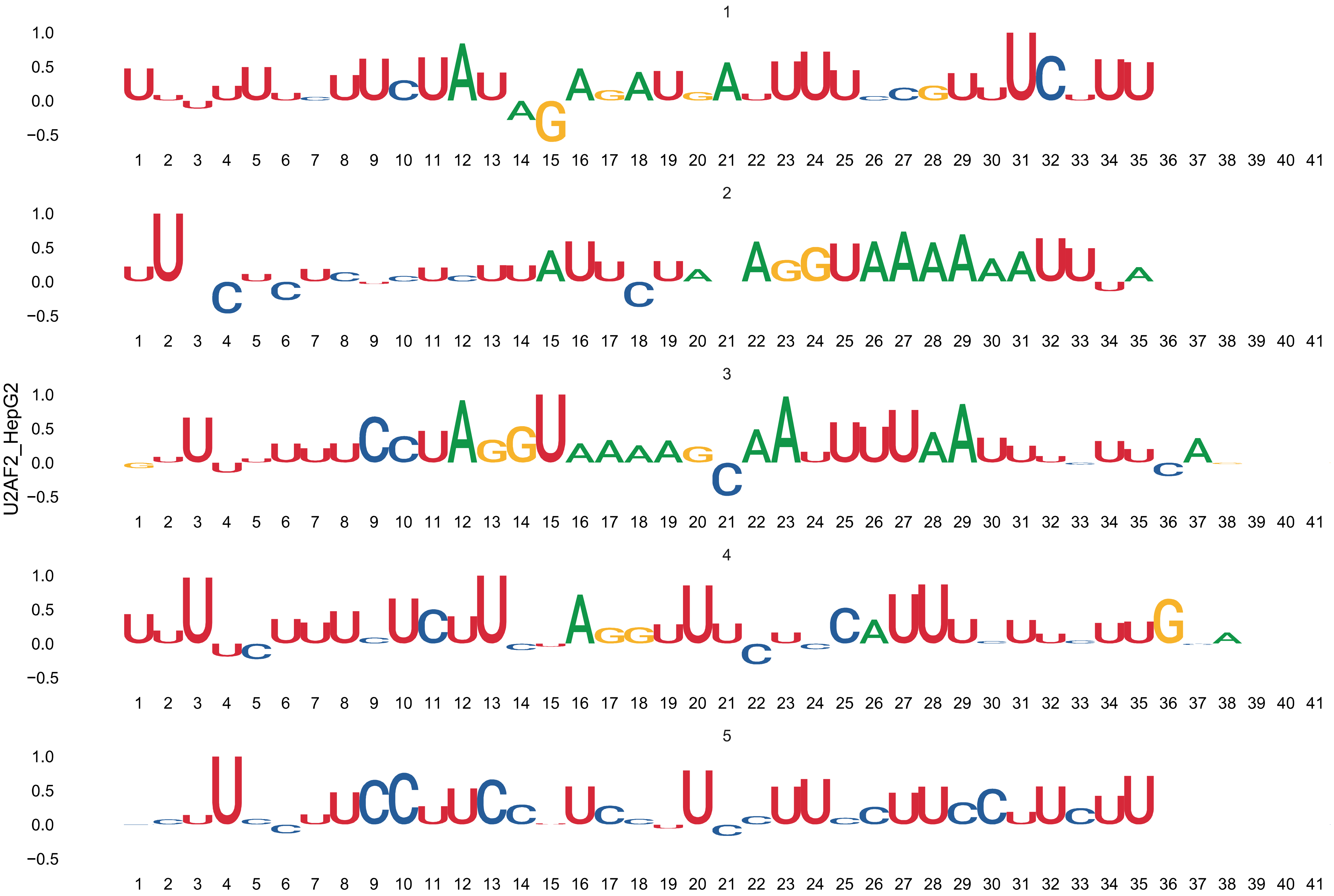
U2AF2_HepG2
1
2
3
4
5
1.0
0.5
0.0
−0.5
