# Supplementary material for: Inferring RNA-binding protein target preferences using adversarial domain adaptation
Source: PLoS Comput Biol. 2022 Feb 24;18(2):e1009863. doi: 10.1371/journal.pcbi.1009863 (PMC8870515; doi:10.1371/journal.pcbi.1009863)
Supplement: S6 Fig — For each RBP, we extracted 5-mers with the highest average attribution scores and derived two consensus motifs. (PDF) [file pcbi.1009863.s011.pdf]

Fig S6

Contents

FMR1_K562	2	FUS_HepG2	21
FUS_K562	3	FXR2_HepG2	22
FXR1_K562	4	HNRNPC_HepG2	23
FXR2_K562	5	HNRNPK_HepG2	24
HNRNPA1_K562	6	HNRNPL_HepG2	25
HNRNPC_K562	7	IGF2BP3_HepG2	26
HNRNPK_K562	8	MATR3_HepG2	27
HNRNPL_K562	9	PABPN1_HepG2	28
IGF2BP2_K562	10	PCBP1_HepG2	29
KHDRBS1_K562	11	PCBP2_HepG2	30
MATR3_K562	12	PTBP1_HepG2	31
PABPC4_K562	13	RBM5_HepG2	32
PCBP1_K562	14	SFPQ_HepG2	33
PTBP1_K562	15	SRSF1_HepG2	34
SRSF1_K562	16	SRSF7_HepG2	35
SRSF7_K562	17	SRSF9_HepG2	36
TARDBP_K562	18	TIA1_HepG2	37
TIA1_K562	19	U2AF2_HepG2	38
U2AF2_K562	20		

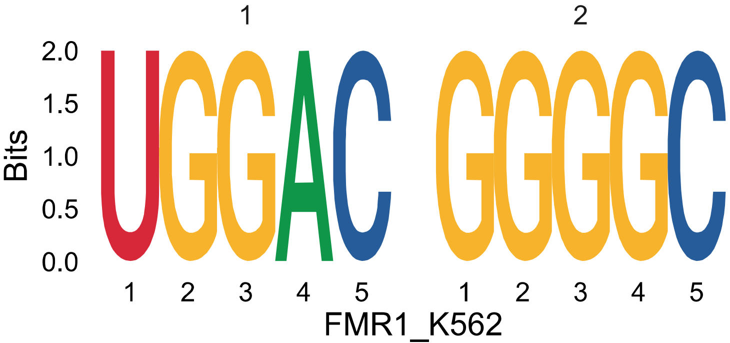

1
2
2.0
1.5
1.0
0.5
0.0
Bits
U G G A C
G G G G C
1 2 3 4 5
1 2 3 4 5
FMR1_K562

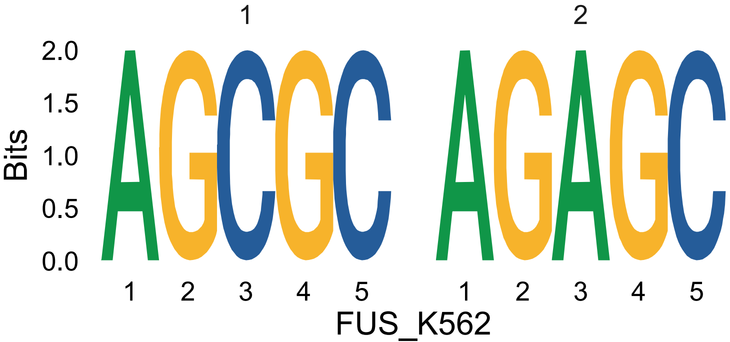

1
2
Bits
2.0
1.5
1.0
0.5
0.0
AGCGC
AGAGC
1 2 3 4 5
1 2 3 4 5
FUS_K562

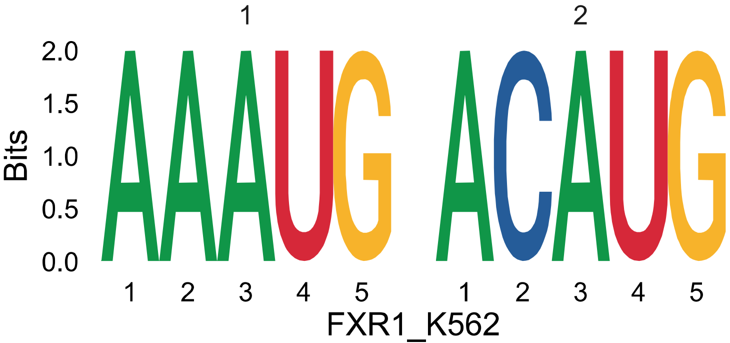
1
2
Bits
2.0
1.5
1.0
0.5
0.0
AAAUG
ACAUG
1 2 3 4 5
1 2 3 4 5
FXR1_K562

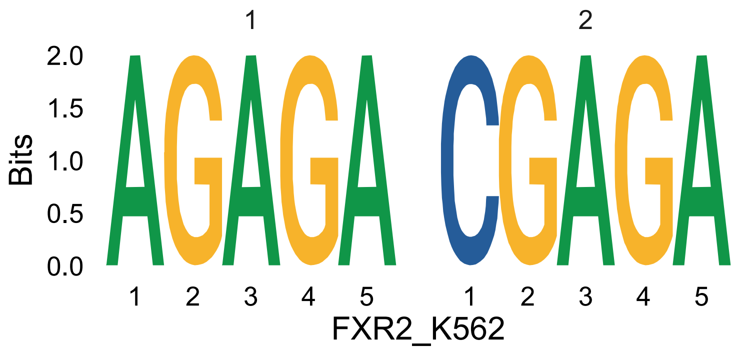
1
2
2.0
1.5
1.0
0.5
0.0
Bits
AGAGA
CGAGA
1 2 3 4 5
1 2 3 4 5
FXR2_K562

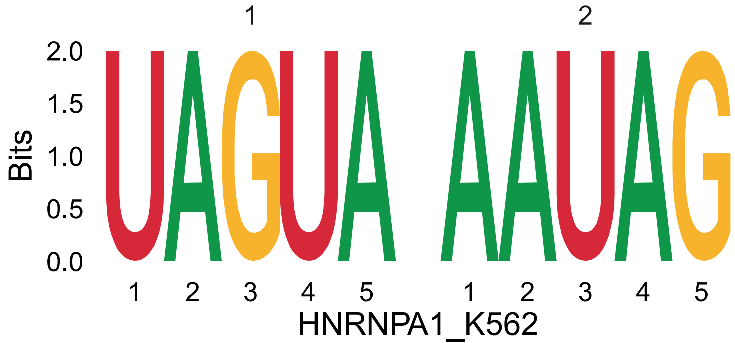
1
2
2.0
1.5
1.0
0.5
0.0
Bits
UAGUA
AAUAG
1 2 3 4 5
1 2 3 4 5
HNRNPA1_K562

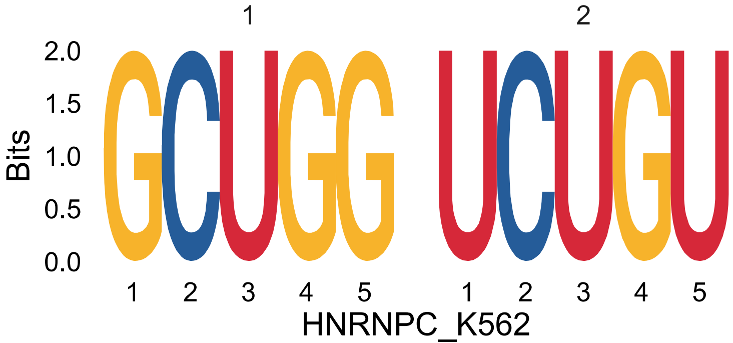

1
2
2.0
1.5
1.0
0.5
0.0
Bits
GCUGG
UCUGU
1 2 3 4 5
1 2 3 4 5
HNRNPC_K562

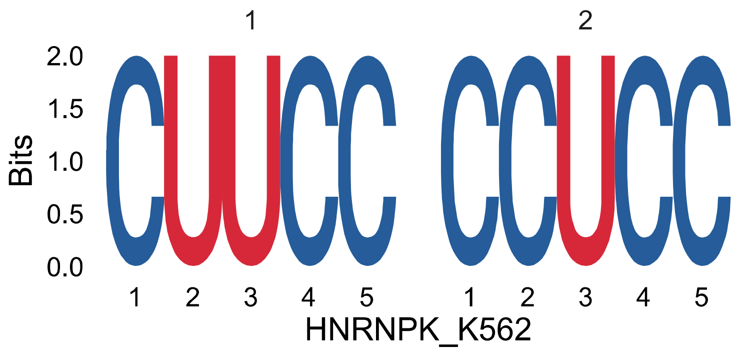
1
2
2.0
1.5
1.0
0.5
0.0
Bits
CUUCC
CCUCC
1 2 3 4 5
1 2 3 4 5
HNRNPK_K562

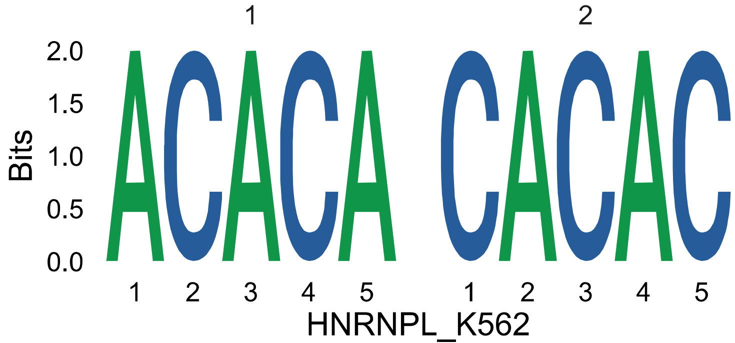

1
2
2.0
1.5
1.0
0.5
0.0
Bits
1 2 3 4 5
1 2 3 4 5
HNRNPL_K562

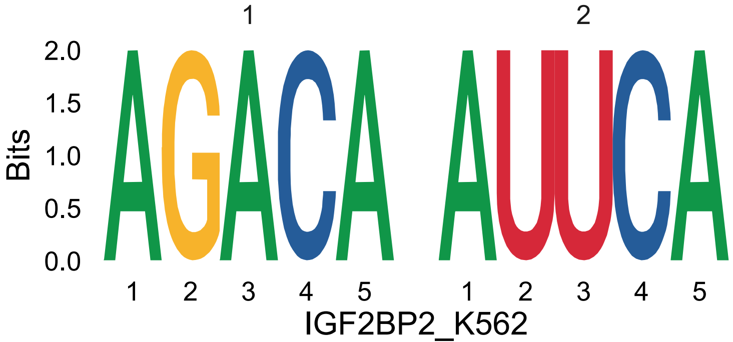
1
2
2.0
1.5
1.0
0.5
0.0
Bits
1 2 3 4 5
1 2 3 4 5
IGF2BP2_K562

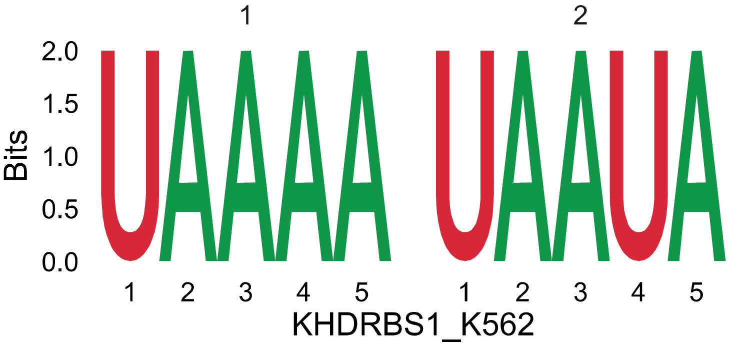

1
2
2.0
1.5
1.0
0.5
0.0
Bits
1 2 3 4 5
1 2 3 4 5
KHDRBS1_K562

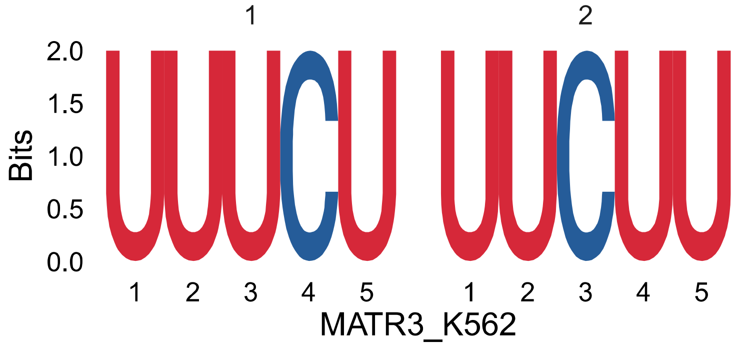

1
2
2.0
1.5
1.0
0.5
0.0
Bits
1 2 3 4 5
1 2 3 4 5
MATR3_K562

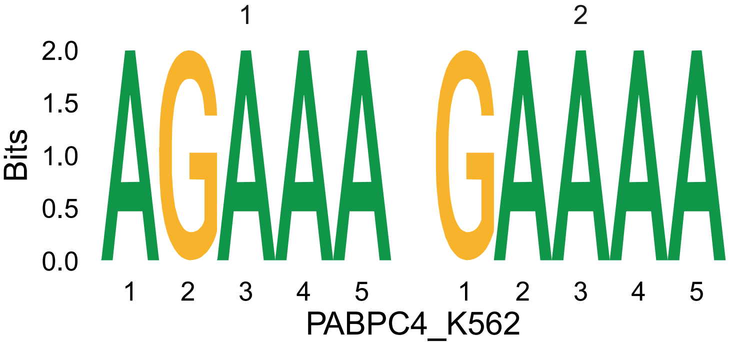
1
2
2.0
1.5
1.0
0.5
0.0
Bits
1 2 3 4 5
1 2 3 4 5
PABPC4_K562

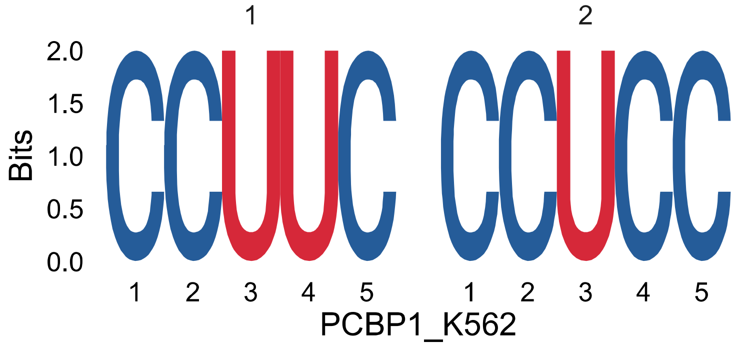

1
2
2.0
1.5
1.0
0.5
0.0
Bits
C C U U C
C C U C C
1 2 3 4 5
1 2 3 4 5
PCBP1_K562

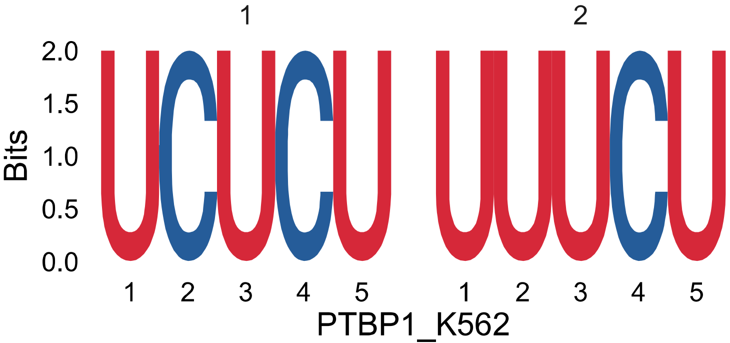

1
2
Bits
2.0
1.5
1.0
0.5
0.0
UCUCU
UUUCU
1 2 3 4 5
1 2 3 4 5
PTBP1_K562

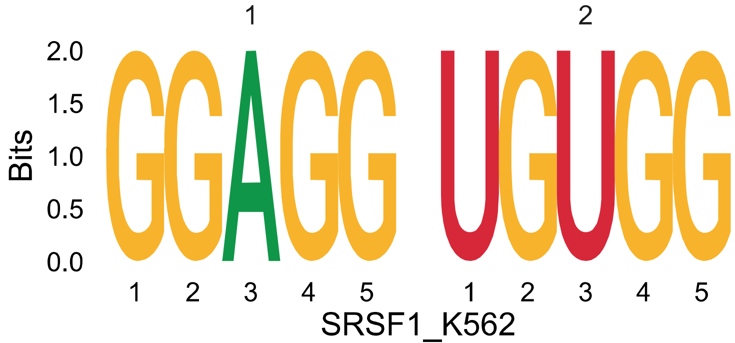

1
2
Bits
2.0
1.5
1.0
0.5
0.0
GGAGG
UGUGG
1 2 3 4 5
1 2 3 4 5
SRSF1_K562

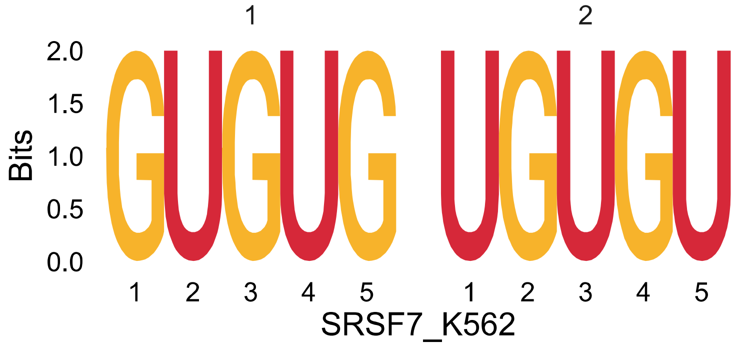
1
2
2.0
1.5
1.0
0.5
0.0
Bits
GUGUG
UGUGU
1 2 3 4 5
1 2 3 4 5
SRSF7_K562

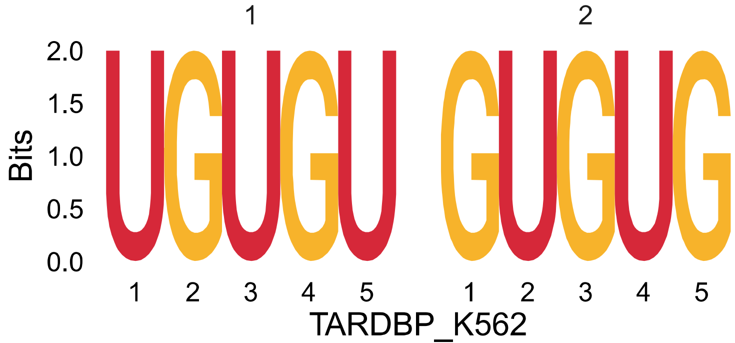
1
2
2.0
1.5
1.0
0.5
0.0
Bits
UGUGU
GUGUG
1 2 3 4 5
1 2 3 4 5
TARDBP_K562

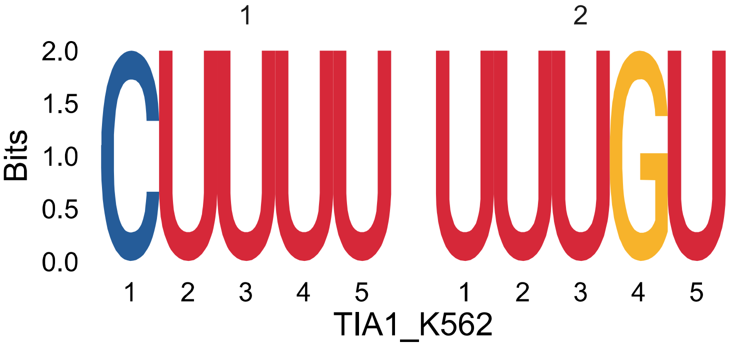
1
2
2.0
1.5
1.0
0.5
0.0
Bits
1 2 3 4 5
1 2 3 4 5
TIA1_K562

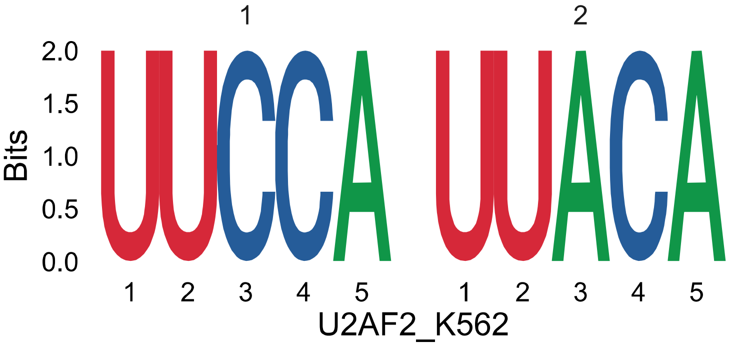
1
2
2.0
1.5
1.0
0.5
0.0
Bits
UUCCA
UUACA
1 2 3 4 5
1 2 3 4 5
U2AF2_K562

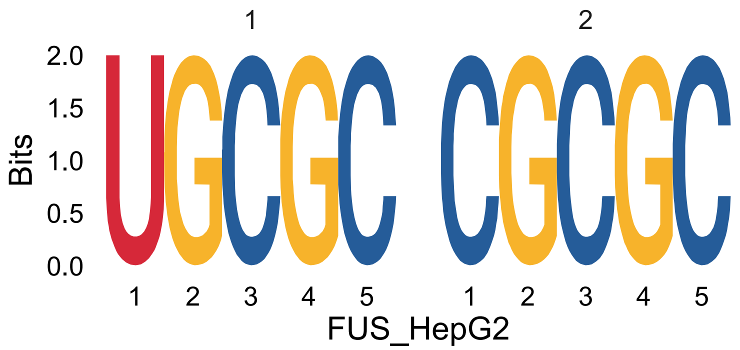

1
2
Bits
2.0
1.5
1.0
0.5
0.0
UGCGC
CGCGC
1 2 3 4 5
1 2 3 4 5
FUS_HepG2

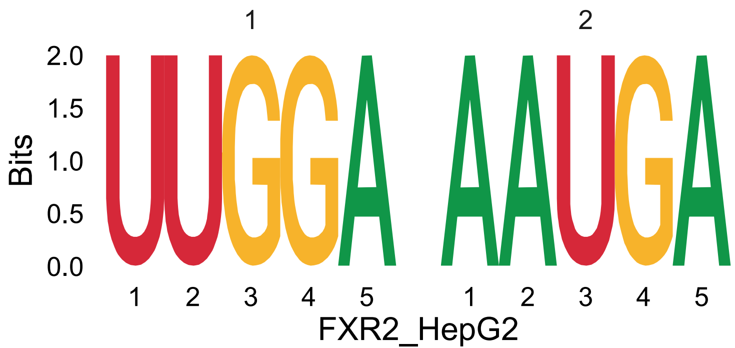

1
2
2.0
1.5
1.0
0.5
0.0
Bits
UUGGA AAUGA
1 2 3 4 5 1 2 3 4 5
FXR2_HepG2

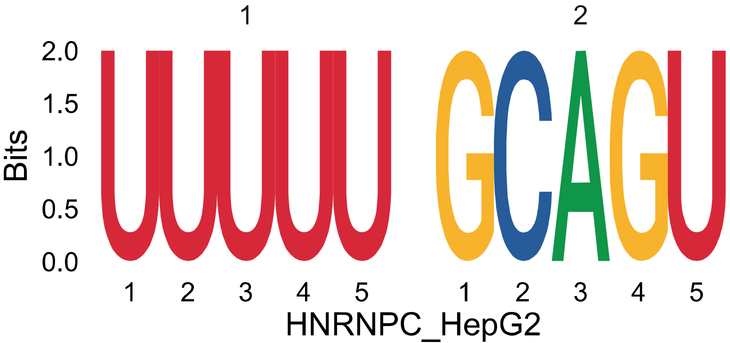
1
2
Bits
2.0
1.5
1.0
0.5
0.0
UUUUU
GCAGU
1 2 3 4 5
1 2 3 4 5
HNRNPC_HepG2

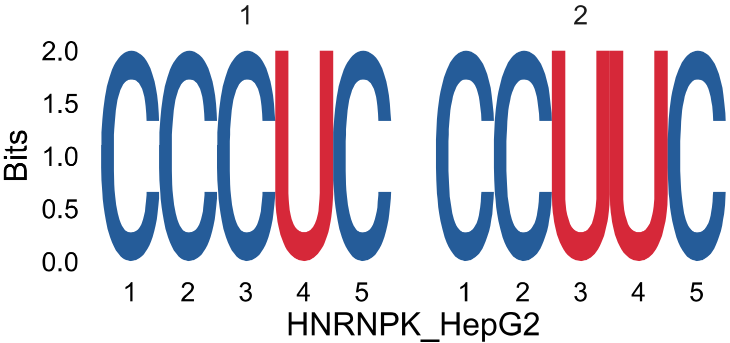
1
2
2.0
1.5
1.0
0.5
0.0
Bits
C C C U C
C C U U C
1 2 3 4 5
1 2 3 4 5
HNRNPK_HepG2

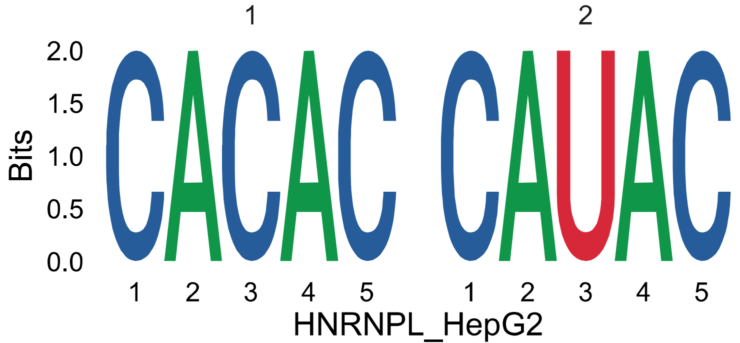
1
2
2.0
1.5
1.0
0.5
0.0
Bits
CACAC
CAUAC
1 2 3 4 5
1 2 3 4 5
HNRNPL_HepG2

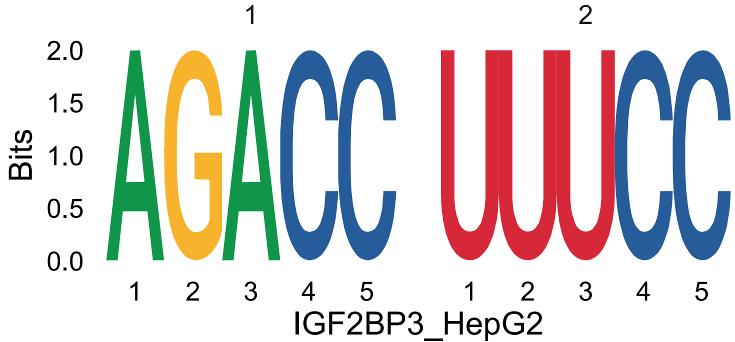

1
2
2.0
1.5
1.0
0.5
0.0
Bits
AGACC
UUUCC
1 2 3 4 5
1 2 3 4 5
IGF2BP3_HepG2

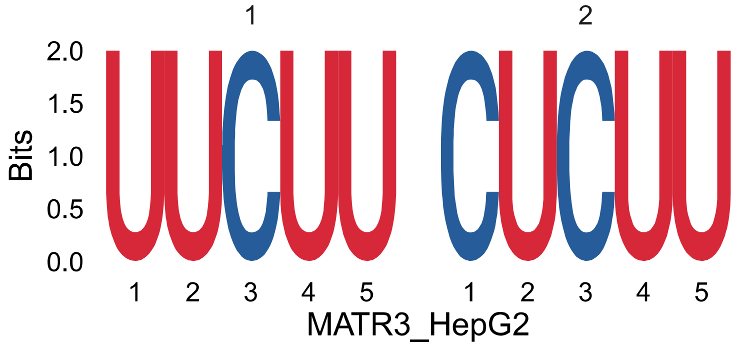
1
2
2.0
1.5
1.0
0.5
0.0
Bits
1 2 3 4 5
1 2 3 4 5
MATR3_HepG2

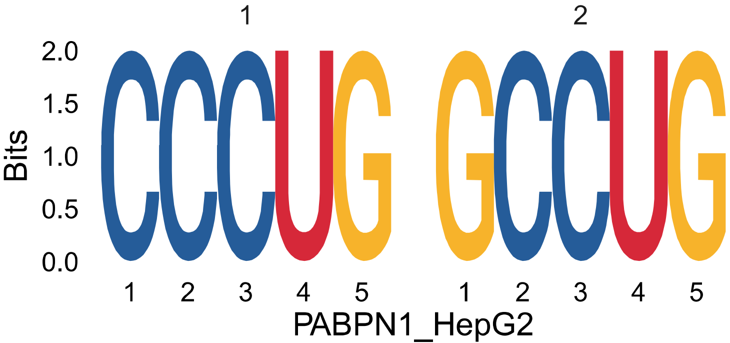

1
2
Bits
2.0
1.5
1.0
0.5
0.0
CCCUG
GCCUG
1 2 3 4 5
1 2 3 4 5
PABPN1_HepG2

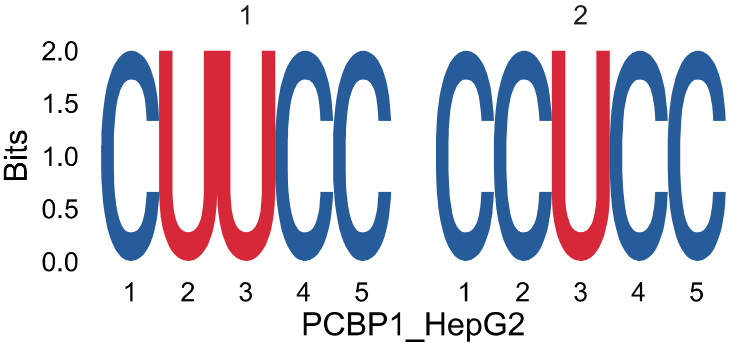
1
2
Bits
2.0
1.5
1.0
0.5
0.0
1 2 3 4 5
1 2 3 4 5
PCBP1_HepG2

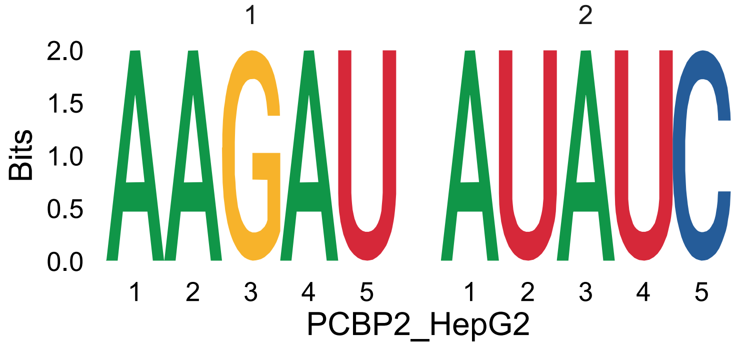

1
2
2.0
1.5
1.0
0.5
0.0
Bits
AAGAU AUAUC
1 2 3 4 5 1 2 3 4 5
PCBP2_HepG2

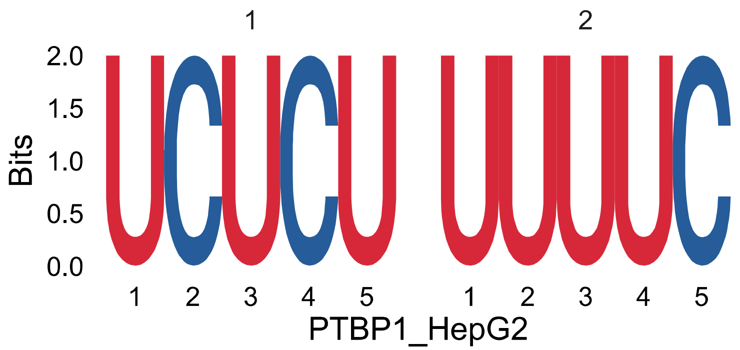
1
2
2.0
1.5
1.0
0.5
0.0
Bits
U C U C U
U U U U C
1 2 3 4 5
1 2 3 4 5
PTBP1_HepG2

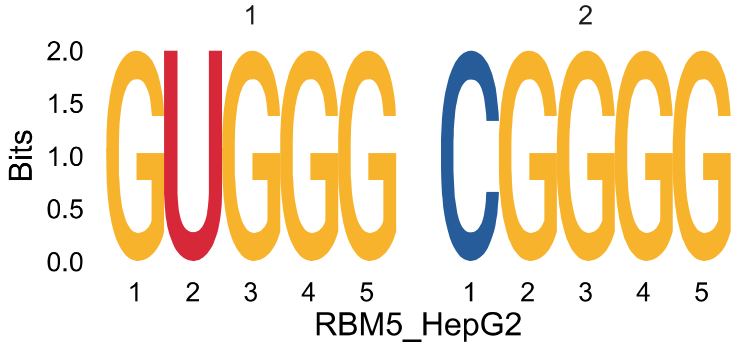
1
2
2.0
1.5
1.0
0.5
0.0
Bits
1 2 3 4 5
1 2 3 4 5
RBM5_HepG2

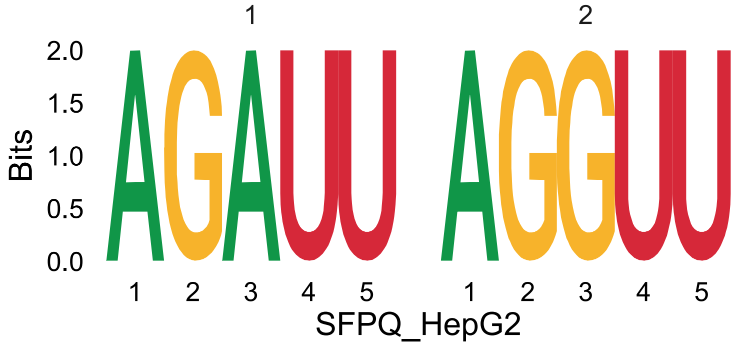
1
2
2.0
1.5
1.0
0.5
0.0
Bits
1
2
3
4
5
1
2
3
4
5
SFPQ_HepG2

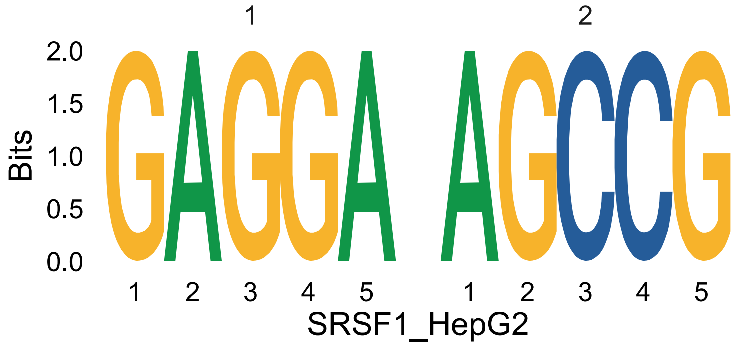
1
2
2.0
1.5
1.0
0.5
0.0
Bits
1 2 3 4 5
1 2 3 4 5
SRSF1_HepG2

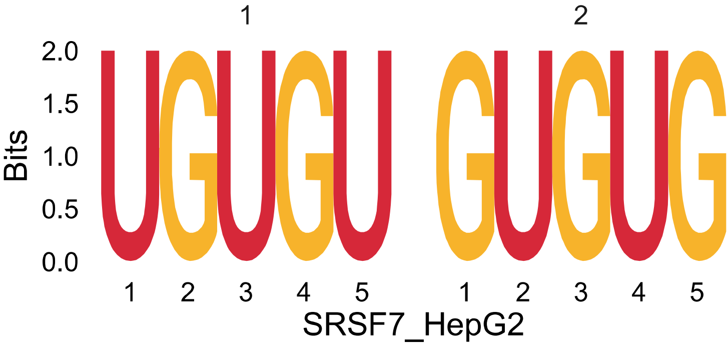

1
2
2.0
1.5
1.0
0.5
0.0
Bits
1
2
3
4
5
1
2
3
4
5
SRSF7_HepG2

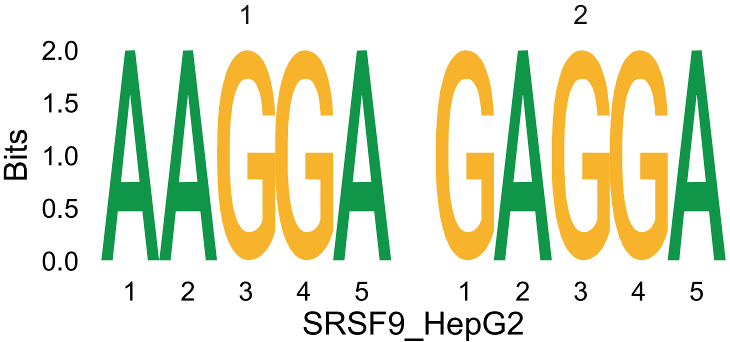
1
2
Bits
2.0
1.5
1.0
0.5
0.0
1
2
3
4
5
1
2
3
4
5
SRSF9_HepG2

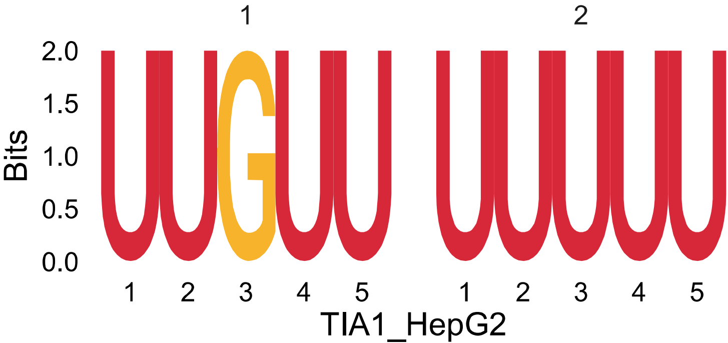

1
2
2.0
1.5
1.0
0.5
0.0
Bits
1 2 3 4 5
1 2 3 4 5
TIA1_HepG2

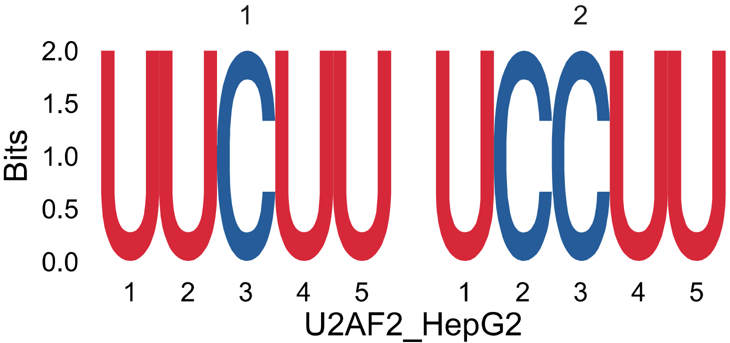
1
2
2.0
1.5
1.0
0.5
0.0
Bits
1 2 3 4 5
1 2 3 4 5
U2AF2_HepG2
